# Determination of Genes Related to Uveitis by Utilization of the Random Walk with Restart Algorithm on a Protein–Protein Interaction Network

**DOI:** 10.3390/ijms18051045

**Published:** 2017-05-13

**Authors:** Shiheng Lu, Yan Yan, Zhen Li, Lei Chen, Jing Yang, Yuhang Zhang, Shaopeng Wang, Lin Liu

**Affiliations:** 1 Department of Ophthalmology, Ren Ji Hospital, School of Medicine, Shanghai Jiao Tong University, Shanghai 200127, China; ludice@163.com (S.L.); hz2004yan@163.com (Y.Y.); lizhen1981_1@126.com (Z.L.); 2 College of Information Engineering, Shanghai Maritime University, Shanghai 201306, China; chen_lei1@163.com; 3 School of Life Sciences, Shanghai University, Shanghai 200444, China; mercuryyangjing@sina.com (J.Y.); wsptfb@163.com (S.W.); 4 Institute of Health Sciences, Shanghai Institutes for Biological Sciences, Chinese Academy of Sciences, Shanghai 200031, China; zhangyh825@163.com

**Keywords:** uveitis, protein–protein interaction, random walk with restart algorithm

## Abstract

Uveitis, defined as inflammation of the uveal tract, may cause blindness in both young and middle-aged people. Approximately 10–15% of blindness in the West is caused by uveitis. Therefore, a comprehensive investigation to determine the disease pathogenesis is urgent, as it will thus be possible to design effective treatments. Identification of the disease genes that cause uveitis is an important requirement to achieve this goal. To begin to answer this question, in this study, a computational method was proposed to identify novel uveitis-related genes. This method was executed on a large protein–protein interaction network and employed a popular ranking algorithm, the Random Walk with Restart (RWR) algorithm. To improve the utility of the method, a permutation test and a procedure for selecting core genes were added, which helped to exclude false discoveries and select the most important candidate genes. The five-fold cross-validation was adopted to evaluate the method, yielding the average F1-measure of 0.189. In addition, we compared our method with a classic GBA-based method to further indicate its utility. Based on our method, 56 putative genes were chosen for further assessment. We have determined that several of these genes (e.g., *CCL4*, *Jun*, and *MMP9*) are likely to be important for the pathogenesis of uveitis.

## 1. Introduction

Uveitis is defined as an inflammation of the uveal tract, which is composed of the ciliary body, iris and choroid [1,2]. Uveitis is one of the leading causes of permanent and irreversible blindness in young and middle-aged people and accounts for 10–15% of blindness in the Western world [1,2,3]. Uveitis can be caused by infectious and non-infectious factors; the latter include Vogt–Koyanagi–Harada (VKH) syndrome, Behcet’s disease (BD), acute anterior uveitis (AAU), birdshot chorioretinopathy (BCR) and some types of cancers. VKH is an autoimmune disease characterized by systemic disorders including poliosis, vitiligo, alopecia, auditory signs and disorders of the central nervous system [4,5]. BD is a chronic multi-systemic inflammatory disease characterized by nongranulomatous uveitis, oral ulcers and skin lesions [2,6]. AAU is the most common non-infectious cause of uveitis and is characterized by self-limiting and recurrent inflammation involving the ciliary and iris body [7]. BCR is a chronic, bilateral, and posterior uveitis that has an almost 100% genetic association with HLA-A29 [8]. Uveitis or uveitis masquerade syndrome could also be induced by some intraocular tumors, such as retinoblastoma and intraocular lymphoma, or their therapeutic approaches [9,10,11,12,13,14,15].

It has been reported that complex genetic mechanisms coupled with an aberrant immune response may be involved in the development of uveitis. In some cases, the pathogenesis of uveitis seemly has a different cause than those described above, such as sarcoidosis [16]. Mutations in different genes and gene families have been discovered in patients. In this study, we focused on the most important causes of uveitis and research for the putative genes involved in these processes. Human leukocyte antigens (*HLA*s) are the major molecules that are important for the development of uveitis, including uveitis associated with VKH (*HLA-DR4*, *DRB1*/*DQA1*), BD (*HLA-B51*), AAU (*HLA-B27*) and BCR (*HLA-A29*). In addition, genome-wide association studies revealed that abnormities of many non-HLA genes such as the interleukin (IL) family and the Signal transducer and activator of transcription 4 (*STAT4*) also participate in the progression of uveitis [17,18,19]. IL23R is associated with both VKH and AAU [20]. Furthermore, copy number variations (CNVs) of Toll-like receptors (*TLR*s), a family of cellular receptors that function in innate immune response, are associated with BD, VKH and AAU. These genes include *TLR*s 1–3, *TLR*s 5–7, and *TLR*s 9–10 [21]. SNPs of TLR4 were also shown to be involved in the development of BD [22]. In addition, it has been demonstrated that there is increased expression of *T-bet* and *IFN-γ*, two genes involved in the Th1 cell pathway, in uveitis patients [23]. The activator of *STAT4* affects *IL-17* production and is a shared risk factor for BD in different cohorts [17,24]. Finally, interleukins (notably *IL-2*, *IL12B*, *IL18* and *IL23R*) are important cytokines that play a pathogenic role in the process of uveitis [2,17,25]. In this study, we mainly focused on the genes that play an important role in the immune system, transcription, or cell adhesion.

Using traditional methods, it is quite difficult to collect these large-scale data and analyze genes synthetically. The microarray is a widely used tool for the identification of novel genes. Microarray analysis has been used to determine a number of genes that are associated with uveitis, including the *IL10* family and several other transcripts [16,26,27,28,29]. In recent years, computational analysis has been applied to identify virulence genes, but many of these genes were identified based on guilt by association (GBA) [30,31,32]. This approach assumes that the candidate genes, which are neighbors of disease genes, are more likely to be new virulence genes. Thus, the GBA-based methods only consider the neighbors of known disease genes to discover novel candidates. Therefore, these methods only examine part of the gene network. Random Walk with Restart (RWR) is another algorithm that identifies disease-related genes [33,34,35]. This algorithm utilizes a set of seed nodes that represent disease genes and performs random walking on the gene network. When the probabilities of all nodes are stable, the probability of a node gene correlating with disease is updated. The genes that correspond to nodes that have high probabilities may be potential novel candidate virulence genes. This method is useful for mining disease genes and to better explore the mechanism of disease. In addition, other studies have adopted the shortest path (SP) algorithm to identify novel disease genes [36,37,38,39,40,41]. By searching the shortest paths that connect any two validated disease genes, genes that are present in these paths could be extracted and considered as novel disease genes. An obvious advantage of the RWR or SP algorithms is that these algorithms utilize the entire gene network and consider more factors, therefore performing a more extensive and reliable analysis.

As discussed above, many genetic factors contribute to the pathogenesis of uveitis. In this study, we utilized computational analyses to build a genetic network based on previously known factors. A computational method was built to identify novel genes related to uveitis. First, a large network was constructed using human protein–protein interactions (PPIs). Next, the RWR algorithm was performed on the network using the validated uveitis-related genes as seed nodes, yielding several possible candidate genes. These candidate genes were filtered based on a set of criteria that were built by *p*-values and their associations with validated uveitis-related genes. To indicate the utility of the method, it was evaluated by five-fold cross-validation, resulting in the average F1-measure of 0.189. Furthermore, the proposed method was compared with a classic GBA-based method [30,31,32] to further prove its effectiveness for identification of uveitis-related genes. Through our method, 56 novel candidate genes were identified and extensively analyzed.

## 2. Results and Discussion

### 2.1. Results of Testing Random Walk with Restart (RWR)-Based Method

Before the RWR-based method was used to identify novel uveitis-related genes, five-fold cross-validation was adopted to evaluate its utility. For each part, the results yielded by the method on the rest four parts were counted as recall, precision and F1-measure, which are listed in Table 1. It can be observed that the average of recall, precision and F1-measure was 0.287, 0.141 and 0.189, respectively. Although these measurements are not very high, the RWR-based method is still acceptable due to the difficulties for identification of novel genes with given functions. Besides, the utility of the RWR-based method would be further proved by comparing it with other methods, which is described in Section 2.5.

### 2.2. RWR Genes

Based on the uveitis-related genes, the RWR algorithm yielded a probability for each gene in the PPI network, which indicated the likelihood of the gene being important for uveitis. Then, genes were selected that had probabilities larger than 10^−5^. From our analysis, we obtained 3641 RWR genes, which are provided with their RWR probabilities in Supplementary Table S1.

### 2.3. Candidate Genes

According to the RWR-based method detailed in Section 3.3, RWR genes were filtered using a permutation test. For each RWR gene, a *p*-value was assigned to indicate whether the RWR gene is specific for uveitis. The *p*-value for each of the 3641 RWR genes is also provided in Supplementary Table S1. We found 1231 candidate genes that had a *p*-value < 0.05 (see the first 1231 genes in Supplementary Table S1).

**Figure 1:**
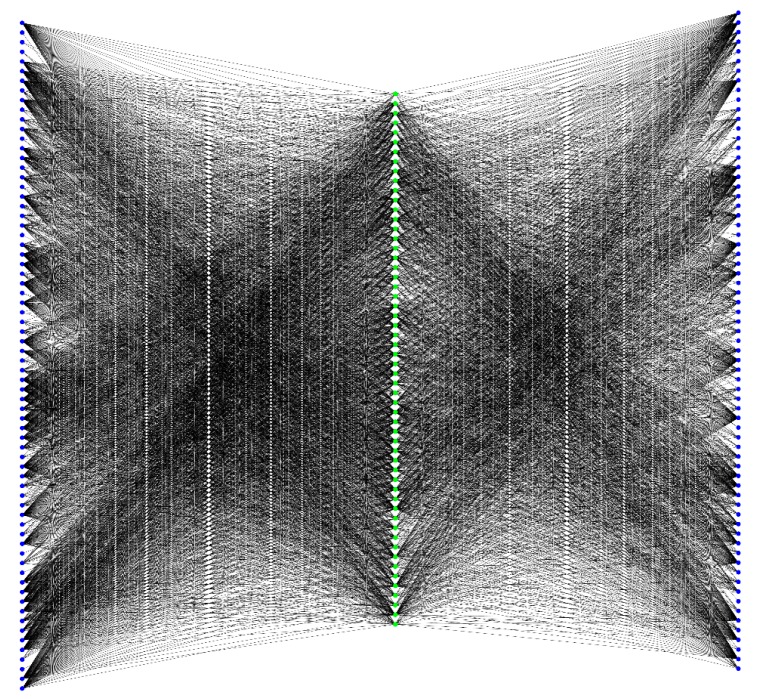
The sub-network of the large network containing Ensembl Identifications (IDs) of validated and putative uveitis-related genes. Blue nodes represent Ensembl IDs of validated uveitis-related genes. Green nodes represent Ensembl IDs of putative uveitis-related genes.

The 1231 candidate genes were then further analyzed using the criteria outlined in Section 3.3. For each candidate gene, MIS (cf. Equation (3)) and MFS (cf. Equation (5)) were calculated, and the values for each gene are available in Supplementary Table S1. The threshold for MIS was set at 900, while 0.8 was used as the threshold for MFS. Finally, we obtained 56 Ensembl IDs (listed in Table 2) corresponding to core candidate genes. These genes were deemed to be highly related to uveitis and could be considered novel candidate genes. As intuitionistic evidence, a sub-network was plotted in Figure 1, which contains the putative and validated genes. Each putative gene had strong associations with validated genes, implying that they had functions similar to those of the validated genes and may be novel uveitis-related genes with high probabilities.

### 2.4. Analysis of Novel Genes

In this study, the RWR-based method yielded fifty-six genes that were deemed to have a significant correlation with uveitis. Detailed information for these genes is provided in Table 2.

#### 2.4.1. Immune System Regulation Genes

*CCL4* (C-C motif chemokine ligand 4) belongs to the cytokine family and is involved in immunoregulation and inflammation. It has been reported that *CCL4* is associated with BD immunopathogenesis [42]. In the majority of VKH cases, the expression of another family member *CCL17* was lower in cerebrospinal fluid than in serum, which indicated its potential function in VKH [43]. *CCL17* also could be inhibited by overexpression of *SOCS1* in the retina to regulate the recruitment of inflammatory cells [44]. The cytokine *CCL20* was considered to be a specific biomarker of *HLA-B27*-associated uveitis [45]. Our study revealed that *CCL4*, *CCL17* and *CCL20* likely play essential roles in uveitis.

*CD40* ligand (also known as *CD154*) is a type II transmembrane glycoprotein that has structural homology to the proteins of the *TNF* (tumor necrosis factor) family [46,47,48,49]. The interaction between the *CD40* and *CD40 ligand* is important for both cellular and humoral immune responses [50]. The *CD40* and *CD40 ligand* interaction provides signals in T-cell priming and effecter functions [46,48,49,51,52,53], whereas monocyte and B-cell apoptosis could be inhibited by their interaction [54]. It has been demonstrated that the *CD40* ligand is associated with the immune-pathogenesis of several autoimmune diseases including AU (anterior uveitis) [54,55]. The *CD40* ligand is significantly expressed on T-cells in the peripheral blood of patients with AU [56]. The results of the RWR-based method revealed a MIS of 999 had a *p*-value < 0.001. Expression of *CD80* on dendritic cells (DCs) could be induced by activation of *NOD1* and *NOD2* and is involved in the pathogenesis of VKH syndrome [57]. In another report, it was found that BBR downregulated the expression of costimulatory molecules *CD40*, *CD80* and *CD86* on *DC*s [58]. The MIS and *p*-value of *CD80* were 999 and <0.001, respectively. We speculate that these molecules play key roles in uveitis, but their mechanism in uveitis must still be clarified.

*CSF2* (colony stimulating factor 2) is a cytokine that functions as a hematological cell growth factor by stimulating stem cells to produce granulocytes and monocytes [59]. Three signaling pathways can be activated by *CSF2*: the JAK2/STAT pathway, the MAP pathway and the PI3K pathway [60,61,62,63,64]. *CSF2* is a valuable prognostic indicator and a therapeutic target in tumors [59]. *CSF2* expression in uveitis is reported as rare. However, in this study, the MIS of *CSF2* was 992 with a *p*-value < 0.001. We speculate that *CSF2* might be a key factor in the pathogenesis of uveitis.

Interleukins and their receptors are inflammatory cytokines that play an important role in immune system response. Many interleukins and their receptors are involved in uveitis, as discussed above. Our data showed that *IL13*, *IL15*, *IL1A*, *IL1B*, *IL1RN*, *IL4*, *IL5*, *IL9*, *IL23A* and *IL2RA* had MISs larger than 900 with *p*-values <0.05. It has been observed that the expression of *IL1A* is decreased in patients with clinically active BD, while the expression of *IL1B* is increased in patients with active, inactive or ocular BD [65]. IL1B has been associated with ocular Behcet’s disease [66]. *IL-13* is a strong immunomodulatory cytokine which is a promising mode of treatment for uveitis [67,68,69,70]. *IL-15* and its receptor system is involved in the inflammatory process and pathogenesis of BD and the IL-15/Fc fusion protein has been shown to inhibit IRBP1-20 specific CD80+ T cell to decrease the severity of EAU [71,72]. An aberrantly high CNV of *IL23A* is a common risk factor for VKH and BD [73]. In mice, *IL-1RN* suppresses immune-mediated ocular inflammation and is considered a potential biomarker in the management of patients with uveitis [74]. Interleukin 2 receptor α (*IL2RA*) is a risk locus in various autoimmune diseases and a variant of this gene, *rs2104286*, was demonstrated to be strongly associated with intermediated uveitis [75]. An antibody against *IL2RA*, daclizumab is used to reduce intermediated uveitis [76]. However, *rs2104286* was not related to endogenous non-anterior uveitis [77]. EAU (experimental autoimmune uveoretinitis) disease severity was reduced in mice in which *IL-1B* expression was reduced in the retina through deletion of S100B, a Ca^2+^ binding protein [78]. In a Lewis rat model of EAU, *IL-2* and *IL-4* were produced in destructive foci in the retina and uveal tract. *IL-2* is thought to act as a cytotoxic effector, while *IL-4* is associated with a helper cell function [79]. In patients with BD, *IL-2* is more highly expressed, while *IL-4* is more lowly expressed [80]. Genetic findings suggest that more work should be done to evaluate both the molecular target and the inhibitor for personalized therapy.

*TLR2*, *TLR3*, *TLR7* and *TLR9* belong to the Toll-like receptor (*TLR*) family, which are key factors in pathogen recognition and activation of innate immunity. *TLR*s are thought to be associated with infection and auto-inflammatory or autoimmune diseases, including uveitis [81,82]. Several autoimmune diseases, including BD, are associated with certain *TLR* gene polymorphisms [83,84]. A significant association has been found between polymorphism of TLR2 and ocular BD patients [85]. The expression of *TLR4* was significantly up-regulated in monocyte-derived macrophages from VKH patients [86]. The chitosan-mediated TLR3-siRNA transfection had a potential therapeutic effect on remitting uveitis [87]. In a Chinese Han population, a high copy number of TLR7 conferred risk for BD patients [88]. In the Japanese population, the homozygous genotypes and homozygous deplotype configuration of TLR9 SNPs was associated with the susceptibility to BD [89]. It has been reported that glucocorticoid could improve uveitis by downregulating *TLR7* and *TLR9* in peripheral blood of patients with uveitis [90]. In our analysis, *TLR2*, *TLR3*, *TLR7* and *TLR9* have MIS scores of 968, 966, 926 and 958, respectively. We argue that *TLR2*, *TLR3*, *TLR7* and *TLR8* play essential roles in uveitis and thus require more attention.

#### 2.4.2. Transcription Associated Genes

*Jun* (also known as jun proto-oncogene) is a critical subunit of the transcription factor AP1, which is an important modulator of diverse biological processes such as cell proliferation, apoptosis and malignant transformation [91]. Jun is activated through phosphorylation at Ser 63 and Ser 73 by *JNK* [92,93]. A high level of *Jun* has been observed in various types of cancer including non-small cell lung cancer, oral squamous cell carcinoma, breast cancer and colorectal cancer [94,95,96,97,98]. Overexpression of *Jun* has led to aberrant tumor growth and progression and inhibited cell apoptosis [94]. The underlying mechanism of *Jun* as it relates to uveitis is still unclear. In a gene screen assay, it was found that expression of *Jun* showed a significantly higher index in experimental lens-induced uveitis rabbits [99]. In our analysis, *Jun* showed a significant index *p*-value and an MIS of 999; therefore, we propose that Jun may be an essential factor in uveitis.

*STAT1* and *STAT6* encode transcription factors that belong to the *STAT* family, where phosphorylation is activated by receptor associated kinases. Atopic dermatitis associated uveitis may be driven by TH2-mediated inflammation [100]. *IL-4* is a TH2 cytokine, and binding with its receptor can activate *STAT6* via the (Jak) Janus kinase/STAT signaling pathway to promote many immunomodulatory genes [101]. Furthermore, the Stat6 VT (STAT6 V547A/T548A) mouse model of atopic dermatitis exhibited uveitis symptoms [100]. In the STAT family, TH17 cells can be induced by *IL-2* and suppressed by *IL-27*/*STAT1* to contribute to uveitis [102]. In this study, *STAT1* and *STAT6* both had significant *p*-values and MIS of 999, and therefore we hypothesize that *STAT1/6* function has a putative role in uveitis.

#### 2.4.3. Cell Adhesion and Signal Transduction Related Genes

Matrix metalloproteinase (*MMP*) are key factors for the degradation of extracellular matrix components and modification of cytokines, protease inhibitors, and cell surface signaling systems [103,104,105,106]. Polymorphisms on the *MMP-9* promoter can affect the development of visceral involvement in Korean people with BD [107]. In our RWR analysis, *MMP9* had an MIS of 971 and a *p*-value < 0.001, which suggests that *MMP-9* may be a novel susceptibility gene for uveitis.

*VCAM1* (vascular cell adhesion molecule 1) belongs to the Ig superfamily and is a cell surface sialoglycoprotein expressed by cytokine-activated endothelium. This protein is mediated by leukocyte-endothelial cell adhesion and signal transduction [108,109]. *VCAM1* can be regulated by inflammatory cytokines such as *IL1B* [108]. Uveitis is closely associated with the immune system and immune-related proteins including the interleukin family. In our study, the MIS of *VCAM1* was 968 and the *p*-value was less than 0.05, which makes *VCAM1* a candidate gene for uveitis.

**Figure 2:**
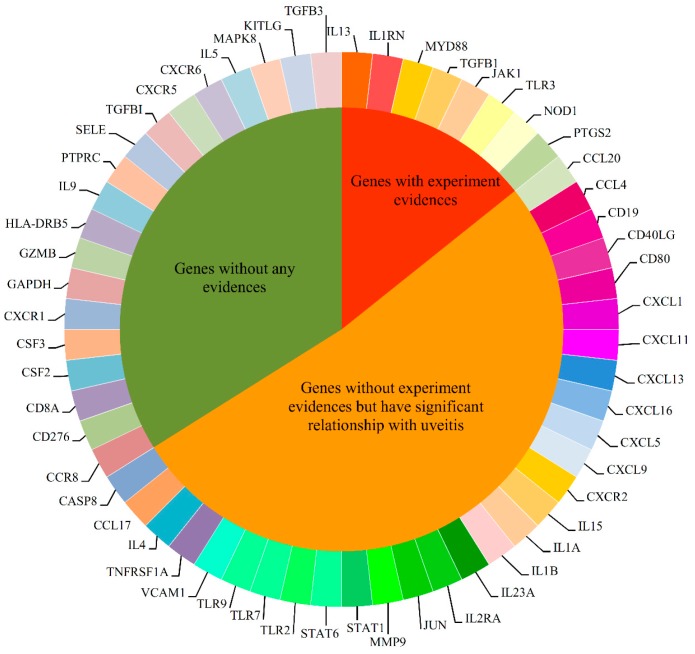
Clustering results of the 56 novel genes according to their evidences for being novel uveitis-related genes. Among 56 novel genes, eight have experiment evidence, 29 have significant relationship with uveitis but without experiment evidence, while no evidence can be found for the rest genes.

We detected 56 novel uveitis-related genes using the RWR-based method. These genes can be clustered into three categories, shown in Figure 2. Among these 56 potential genes, eight (8/56, 14.3%) genes, *IL13*, *IL1RN*, *JAK1*, *MYD88*, *NOD1*, *PTGS2*, *TGFB1* and *TLR3*, were considered as uveitis genes by experimental evidence [110,111,112,113,114,115,116,117], and 29 (29/56, 51.8%) genes (*CASP8*, *CCL17*, *CCL20*, *CCL4*, *CD19*, *CD40 LG*, *CD80*, *CXCL1*, *CXCL11*, *CXCL13*, *CXCL16*, *CXCL5*, *CXCL9*, *CXCR2*, *IL15*, *IL1A*, *IL1B*, *IL23A*, *IL2RA*, *IL4*, *JUN*, *MMP9*, *STAT1*, *STAT6*, *TLR2*, *TLR7*, *TLR9*, *TNFRSF1A* and *VCAM1*) had a correlation with uveitis. However, the pathogenesis is not clear. In our results, we found that these genes have a significant relationship with uveitis genes and therefore need more validation. There are few reports of the rest of the genes (19/56, 33.9%) (*CCR8*, *CD276*, *CD8A*, *CSF2*, *CSF3*, *CXCR1*, *CXCR5*, *CXCR6*, *GAPDH*, *GZMB*, *HLA-DB5*, *IL5*, *IL9*, *KITLG*, *MAPK8*, *PTPRC*, *SELE*, *TGFB3* and *TGFBI*) which participate in the process of uveitis. We considered that they might be novel uveitis genes and merit attention. We argue that some of these may be critical putative virulence genes for uveitis and could be interesting agents for the treatment of human uveitis.

### 2.5. Comparison of Other Methods

The results listed in Section 2.1, Section 2.2, Section 2.3 and Section 2.4 can partly prove the effectiveness of the RWR-based method. In this section, we compared our method with a classic GBA-based method [30,31,32], i.e., a method like the nearest neighbor algorithm (NNA). This method identified novel genes from neighbors of the uveitis-related genes in a network. For convenience, we directly used the PPI network that was adopted in the RWR-based method. In addition, we called a neighbor of a node is a nearer neighbor if the edge between them was assigned a higher weight due to the definition of the interaction score reported in STRING. The GBA-based method selected the *k* nearest neighbors of each uveitis-related genes and collected them together as the predicted genes of the method, where *k* is a predefined parameter.

The five-fold cross-validation method was also adopted to test the GBA-based method, which used the same partition in testing the RWR-based method. Because we do not know the best value of *k*, we tried the following values: 1, 2, 3, 4, 5, 6, 7, 8, 9, 10, 20, 30, 40, 50, 60, 70, 80, 90, and 100. The testing results are provided in Supplementary Table S2. The best performance of the GBA-based method with different parameter *k* on each part is shown in Table 3. Compared with the testing results of RWR-based method, also listed in Table 3 for convenience, we can see that GBA-based method provides higher recalls sometimes, however, it always provides lower precisions, indicating the GBA-based method can yield more false positive genes. If only considering the F1-measure, we can conclude that F1-measures of the RWR-based method are always higher than those of the GBA-based method. It is indicated that the RWR-based method is superior to GBA-based method for identification of uveitis-related genes.

## 3. Materials and Methods

### 3.1. Materials

Uveitis-related genes were collected from literatures indexed by PubMed (http://www.ncbi.nlm.nih.gov/pubmed/). The keywords “uveitis” and “genes” were used to search the literature in PubMed, which resulted in the collection of 744 papers. Among them, 98 review papers that generally summarized uveitis-related genes were manually reviewed. From those 98 papers, 121 genes were chosen from 96 reviews reporting the functional genes that may be important for uveitis or for specific uveitis symptoms. These genes are provided in Supplementary Table S3. In total, 146 Ensembl IDs for these genes were also determined and are provided in Supplementary Table S3.

### 3.2. Protein-protein Interaction (PPI) Network

PPIs are useful for the investigation of genetic disorders because they play an essential role in intracellular and intercellular biochemical processes. Many computational methods have been developed using this information, such as the prediction engines for the identification of protein functions [118,119,120] and methods for identification of novel disease genes [36,37,38]. Several methods were built based on the hypothesis that two proteins in a PPI are more likely to share similar functions. Thus, we can infer novel genes related to uveitis using PPI information and the uveitis-related genes mentioned in Section 3.1.

In this study, we used the PPI information retrieved from STRING (Search Tool for the Retrieval of Interacting Genes/Proteins, Version 9.1, http://string-db.org/) [121] to construct the PPI network that the RWR algorithm can be applied. To access the PPI information in STRING, we downloaded the file “protein.links.v9.1 txt.gz”. Because “9606” is the organism code for the human interactome in STRING, lines in this file that started with “9606” were extracted, obtaining 2,425,314 human PPIs involving 20,770 proteins. According to STRING, these PPIs were derived from the following four sources: (1) genomic context; (2) high-throughput experiments; (3) (conserved) co-expression; and (4) previous knowledge. Thus, the information in STRING contained both the direct (physical) and the indirect (functional) association between proteins, therefore STRING could widely measure the associations between proteins. Each PPI contained two Ensembl IDs and one score that ranged between 150 and 999, which indicated the strength of the interaction. An interaction with a high score meant this interaction has a high probability of occurring. For each interaction containing proteins *p_a_* and *p_b_*, the score was denoted by *S*(*p_a_*,*p_b_*). The PPI network defined the 20,770 proteins as the nodes, and two nodes were adjacent if and only if their corresponding proteins can form a PPI. Additionally, each edge in the network represented a PPI; thus, we assigned a weight to each edge, which was defined as the score of its corresponding PPI. From our analysis, a PPI network containing 20,770 nodes and 2,425,314 edges was obtained.

### 3.3. RWR-Based Method

The RWR algorithm was executed on the PPI network using validated genes as seed nodes to search possible genes. Then, a permutation test was executed to exclude false discoveries found by RWR. The remaining candidate genes with strong associations to validated genes were selected for further analysis. The pseudo-codes of the RWR-based method are listed in Table 4.

#### 3.3.1. Searching Possible Genes Using the RWR Algorithm

RWR is a type of ranking algorithm [33]. Based on a seed node or a set of seed nodes, it simulates a walker that starts from the nodes and randomly walks in a network. Here, 146 Ensembl IDs listed in Supplementary Table S3 were deemed as seed nodes. Starting from these nodes, we attempted to discover novel nodes (genes) related to uveitis. In the beginning of the RWR algorithm, a 20,770-D vector *P*_0_ was constructed, in which each composition represented the probability that a node in the network was a uveitis-related gene. Because the 146 Ensembl IDs represented validated uveitis-related genes, their compositions in *P*_0_ were set to 1/146, while others were set to zero. Then, the RWR algorithm repeatedly updated this probability vector until it became stable. We designated *P_i_* to represent the probability vector after the *i*-th step was executed. The probability vector was updated according to the following equation:

(1) $$P_{i+1}=(1-r)A^{T}P_{i}+rP_{0}$$

where *A* represented the column-wise normalized adjacency matrix of the PPI network and *r* was set to 0.8. When ${\left\|P_{t+1}-P_{t}\right\|}_{L_{1}}<10^{-6}$, the update procedure was stopped, and *P_t_*_+1_ was the output of the RWR algorithm.

According to the probability vector yielded by the RWR algorithm, some nodes received high probabilities. It was apparent that their corresponding genes are more likely to be uveitis-related genes. To avoid missing possible uveitis-related genes, we set a probability threshold of 10^−5^. The corresponding genes of these nodes were designated as RWR genes.

In this study, we used the RWR program on the heterogeneous network that was implemented in Matlab and proposed by Li and Patra [122]. The code can be downloaded at http://www3.ntu.edu.sg/home/aspatra/research/Yongjin_BI2010.zip. By setting the special values of some parameters, this program could be used to execute the RWR algorithm on a single network.

#### 3.3.2. Excluding False Discoveries Using the Permutation Test

Based on the validated uveitis-related genes and RWR algorithm, new RWR genes were accessed. However, this result was influenced by the structure of the constructed PPI network, i.e., some RWR genes were selected due to the structure of the network and they were not necessarily unique to uveitis. Furthermore, if we randomly selected some nodes in the network as seed nodes of the RWR algorithm, these genes were still selected for and were therefore deemed as likely to be false positive. To control for these genes, a permutation test was executed. We randomly constructed 1000 Ensembl ID sets, denoted by $E_{1},E_{2},\ldots ,E_{1000}$, consisting of 146 Ensembl IDs. For each set, the Ensembl IDs were deemed seed nodes of the RWR algorithm. Each RWR gene was given a probability. Thus, there were 1000 probabilities for 1000 sets and one probability for 146 Ensembl IDs of the uveitis-related genes for each RWR gene. Then, a measurement, called the *p*-value, was counted for each RWR gene *g*, which was defined as:

(2) $$p-\mathrm{value}(g)=\frac{\Theta }{1000}$$

where Θ represented the number of randomly constructed sets where the probability assigned to *g* was larger than that for the 146 Ensembl IDs of uveitis-related genes. Clearly, an RWR gene with a high *p*-value indicated that the gene was not specific for uveitis and should be discarded. RWR genes with *p*-values less than 0.05 were selected for further analysis as potential candidate genes for uveitis.

#### 3.3.3. Selection of Core Genes by Associations with Validated Genes

We hypothesized that, of the candidate genes, some may have a strong correlation with uveitis. To further select core candidate genes, two criteria were designed. Candidate genes satisfying both criteria were selected for additional analysis. Candidate genes that had the strongest associations with uveitis-related genes were more likely to be novel uveitis-related genes. Thus, for each candidate gene *g*, we calculated the maximum interaction score (MIS) as follows:

(3) $$MIS(g)=\max\{S(g,g^{\prime }):g^{\prime }\mathrm{is}\;a\;\mathrm{uveitis}-\mathrm{related}\;\mathrm{gene}\}$$

A high MIS suggested that the candidate gene was closely related to at least one uveitis-related gene, indicating that it was a novel uveitis-related gene with a high probability. According to STRING, a score of 900 was the cut-off for the highest confidence level. Therefore, candidate genes with MISs larger than 900 were selected.

Validated uveitis-related genes have strong associations with specific gene ontology (GO) terms and Kyoto Encyclopedia of Genes and Genomes (KEGG) pathways. Therefore, candidate genes that had similar associations with uveitis GO terms and KEGG pathways were more likely to be novel uveitis-related genes. We performed GO term (KEGG pathway) [123,124,125,126] enrichment analysis for candidate genes and uveitis-related genes. The representation of a gene *g* on all GO terms and KEGG pathways was encoded into a vector ES (g) using this theory. This vector can be obtained by an in-house program using the R function phyper. The R code used was “score <− −log10 (phyper (numWdrawn− 1, numW, numB, numDrawn, lower.tail = FALSE)),” where numW, numB, and numDrawn are the number of genes annotated to the GO term or KEGG pathway, the number of genes not annotated to the GO term or KEGG pathway, and the number of neighbors of gene g and numWdrawn is the number of neighbors of gene g that are also annotated to the GO term or KEGG pathway. The relativity of the two genes g and $g^{\prime }$ on GO terms and KEGG pathways was measured by

(4) $$\Gamma (g,g^{\prime })=\frac{ES(g)\cdot ES(g^{\prime })}{\left\|ES(g)\right\|\cdot \left\|ES(g^{\prime })\right\|}$$

A high outcome of Equation (4) indicated that *g* and $g^{\prime }$ have a similar relationship in terms of GO terms and KEGG pathways. For any candidate gene *g*, we calculated the maximum function score (MFS) using the following equation:

(5) $$MFS(g)=\max\{\Gamma (g,g^{\prime }):g^{\prime }\mathrm{is}\;a\;\mathrm{uveitis}-\mathrm{related}\;\mathrm{gene}\}$$

Candidate genes with high MFSs were selected. In this equation, we set 0.8 as the threshold of MFS to select essential candidate genes.

### 3.4. Methods for Testing RWR-Based Method

In this study, we designed the RWR-based method to identify novel uveitis-related genes. However, it is necessary to test its effectiveness in advance. Here, the five-fold cross-validation [127] was employed. In detail, 146 Ensembl IDs of uveitis-related genes were randomly and equally divided into five parts. Then, Ensembl IDs in each part were singled out in turn and other Ensembl IDs in the rest four parts were used as the seed nodes in the RWR-based method. For each part, the results yielded by a good identification method on the rest four parts should satisfy the following conditions: (I) the results can recover a high proportion of the Ensembl IDs in the part; and (II) the results cannot contain several Ensembl IDs that are not in the part. Thus, recall and precision were employed to evaluate the results yielded by the RWR-based method, which can be calculated by

(6) $$\left\{ \begin{array}{l} \mathrm{recall}=\frac{TP}{TP+FN}\\ \mathrm{precision}=\frac{TP}{TP+FP} \end{array}\right.$$

where *TP* represented the number of Ensembl IDs in the part that can be recovered by the method, *FN* represented the number of Ensembl IDs in the part that cannot be recovered by the method and *FP* represented the number of Ensembl IDs that were yielded by the method and not in the part. In addition, to evaluate the predicted results on the whole, the F1-measure was also adopted, which can be computed by

(7) $$F1-\mathrm{measure}=\frac{2\times \mathrm{recall}\times \mathrm{precision}}{\mathrm{recall}+\mathrm{precision}}$$

It is clear that a high F1-measure means the good performance of the method.

## 4. Conclusions

This study presented a computational method to determine novel uveitis-related genes. Using the RWR algorithm and certain screening criteria, 56 putative genes were accessed. Extensive analysis of the obtained genes confirmed that several genes are associated with the pathogenesis of uveitis. We hope that the identified novel genes may be used as material to study uveitis and that the proposed method can be extended to other diseases.

## Figures and Tables

**Table 1:** The performance of the Random Walk with Restrart (RWR)-based method yielded by five-fold cross-validation.

Index of Part	Recall	Precision	F1-Measure
1	0.172	0.089	0.118
2	0.172	0.088	0.116
3	0.379	0.177	0.242
4	0.310	0.141	0.194
5	0.400	0.211	0.276
Mean	0.287	0.141	0.189

**Table 2:** Novel genes inferred by Random Walk with Restrart (RWR)-based method.

Ensembl ID	Gene Symbol	Description	Probability	*p*-Value	MIS	MFS
ENSP00000351671 ^b^	*CCL20*	C-C motif chemokine ligand 20	1.65 × 10^−4^	<0.001	999	0.841
ENSP00000250151 ^b^	*CCL4*	C-C motif chemokine ligand 4	1.64 × 10^−4^	<0.001	994	0.820
ENSP00000326432 ^c^	*CCR8*	C-C motif chemokine receptor 8	8.90 × 10^−5^	<0.001	951	0.816
ENSP00000313419 ^b^	*CD19*	CD19 molecule	2.15 × 10^−4^	<0.001	947	0.837
ENSP00000320084 ^c^	*CD276*	CD276 molecule	1.91 × 10^−4^	<0.001	955	0.823
ENSP00000359663 ^b^	*CD40LG*	CD40 ligand	1.97 × 10^−4^	<0.001	999	0.839
ENSP00000264246 ^b^	*CD80*	CD80 molecule	2.18 × 10^−4^	<0.001	999	0.820
ENSP00000283635 ^c^	*CD8A*	CD8a molecule	1.91 × 10^−4^	<0.001	990	0.815
ENSP00000296871 ^c^	*CSF2*	Colony stimulating factor 2	2.71 × 10^−4^	<0.001	992	0.875
ENSP00000225474 ^c^	*CSF3*	Colony stimulating factor 3	1.55 × 10^−4^	<0.001	916	0.829
ENSP00000379110 ^b^	*CXCL1*	C-X-C motif chemokine ligand 1	1.69 × 10^−4^	<0.001	973	0.827
ENSP00000306884 ^b^	*CXCL11*	C-X-C motif chemokine ligand 11	1.28 × 10^−4^	<0.001	999	0.818
ENSP00000286758 ^b^	*CXCL13*	C-X-C motif chemokine ligand 13	1.49 × 10^−4^	<0.001	986	0.806
ENSP00000293778 ^b^	*CXCL16*	C-X-C motif chemokine ligand 16	1.02 × 10^−4^	<0.001	952	0.800
ENSP00000296027 ^b^	*CXCL5*	C-X-C motif chemokine ligand 5	1.11 × 10^−4^	<0.001	958	0.811
ENSP00000354901 ^b^	*CXCL9*	C-X-C motif chemokine ligand 9	2.13 × 10^−4^	<0.001	999	0.883
ENSP00000295683 ^c^	*CXCR1*	C-X-C motif chemokine receptor 1	8.67 × 10^−5^	<0.001	999	0.833
ENSP00000319635 ^b^	*CXCR2*	C-X-C motif chemokine receptor 2	1.02 × 10^−4^	<0.001	999	0.851
ENSP00000229239 ^c^	*GAPDH*	Glyceraldehyde-3-phosphate dehydrogenase	2.12 × 10^−4^	<0.001	922	0.824
ENSP00000216341 ^c^	*GZMB*	Granzyme B	2.46 × 10^−4^	<0.001	991	0.829
ENSP00000364114 ^c^	*HLA-DRB5*	Major histocompatibility complex, class II, DR β 5	2.27 × 10^−4^	<0.001	948	0.822
ENSP00000304915 ^a^	*IL13*	Interleukin 13	1.31 × 10^−4^	<0.001	999	0.813
ENSP00000296545 ^b^	*IL15*	Interleukin 15	1.85 × 10^−4^	<0.001	946	0.806
ENSP00000263339 ^b^	*IL1A*	Interleukin 1 α	1.82 × 10^−4^	<0.001	996	0.820
ENSP00000263341 ^b^	*IL1B*	Interleukin 1 β	3.58 × 10^−4^	<0.001	999	0.873
ENSP00000259206 ^a^	*IL1RN*	Interleukin 1 receptor antagonist	1.68 × 10^−4^	<0.001	999	0.836
ENSP00000228534 ^b^	*IL23A*	Interleukin 23 subunit Α	2.87 × 10^−4^	<0.001	998	0.844
ENSP00000369293 ^b^	*IL2RA*	Interleukin 2 receptor subunit Α	2.46 × 10^−4^	<0.001	999	0.866
ENSP00000274520 ^c^	*IL9*	Interleukin 9	1.27 × 10^−4^	<0.001	965	0.806
ENSP00000360266 ^b^	*JUN*	Jun proto-oncogene, AP-1 transcription factor subunit	3.22 × 10^−4^	<0.001	999	0.831
ENSP00000361405 ^b^	*MMP9*	Matrix metallopeptidase 9	1.70 × 10^−4^	<0.001	971	0.833
ENSP00000379625 ^a^	*MYD88*	Myeloid differentiation primary response 88	1.82 × 10^−4^	<0.001	999	0.882
ENSP00000356346 ^c^	*PTPRC*	Protein tyrosine phosphatase, receptor type C	2.18 × 10^−4^	<0.001	994	0.826
ENSP00000331736 ^c^	*SELE*	Selectin E	1.46 × 10^−4^	<0.001	978	0.830
ENSP00000354394 ^b^	*STAT1*	Signal transducer and activator of transcription 1	2.63 × 10^−4^	<0.001	999	0.852
ENSP00000300134 ^b^	*STAT6*	Signal transducer and activator of transcription 6	1.77 × 10^−4^	<0.001	999	0.804
ENSP00000221930 ^a^	*TGFB1*	Transforming growth factor β 1	2.90 × 10^−4^	<0.001	997	0.832
ENSP00000416330 ^c^	*TGFBI*	Transforming growth factor β induced	1.91 × 10^−4^	<0.001	917	0.813
ENSP00000260010 ^b^	*TLR2*	Toll like receptor 2	2.25 × 10^−4^	<0.001	968	0.888
ENSP00000370034 ^b^	*TLR7*	Toll like receptor 7	1.26 × 10^−4^	<0.001	926	0.819
ENSP00000353874 ^b^	*TLR9*	Toll like receptor 9	1.55 × 10^−4^	<0.001	958	0.854
ENSP00000294728 ^b^	*VCAM1*	Vascular cell adhesion molecule 1	2.23 × 10^−4^	<0.001	968	0.882
ENSP00000292174 ^c^	*CXCR5*	C-X-C motif chemokine receptor 5	1.14 × 10^−4^	0.001	976	0.820
ENSP00000343204 ^a^	*JAK1*	Janus kinase 1	1.21 × 10^−4^	0.001	999	0.818
ENSP00000162749 ^b^	*TNFRSF1A*	TNF Receptor superfamily member 1A	2.30 × 10^−4^	0.001	999	0.826
ENSP00000304414 ^c^	*CXCR6*	C-X-C motif chemokine receptor 6	9.27 × 10^−5^	0.002	964	0.803
ENSP00000296795 ^a^	*TLR3*	Toll like receptor 3	1.58 × 10^−4^	0.002	966	0.858
ENSP00000231454 ^c^	*IL5*	Interleukin 5	1.13 × 10^−4^	0.004	991	0.803
ENSP00000222823 ^a^	*NOD1*	Nucleotide binding oligomerization domain containing 1	7.72 × 10^−5^	0.004	991	0.866
ENSP00000231449 ^b^	*IL4*	Interleukin 4	2.55 × 10^−4^	0.005	999	0.852
ENSP00000356438 ^a^	*PTGS2*	Prostaglandin-endoperoxide synthase 2	1.92 × 10^−4^	0.009	972	0.864
ENSP00000219244 ^b^	*CCL17*	C-C motif chemokine ligand 17	1.20 × 10^−4^	0.01	984	0.808
ENSP00000351273 ^b^	*CASP8*	Caspase 8	9.66 × 10^−5^	0.027	999	0.821
ENSP00000353483 ^c^	*MAPK8*	Mitogen-activated protein kinase 8	1.03 × 10^−4^	0.034	925	0.847
ENSP00000228280 ^c^	*KITLG*	KIT ligand	9.60 × 10^−5^	0.039	958	0.810
ENSP00000238682 ^c^	*TGFB3*	Transforming growth factor β 3	5.37 × 10^−5^	0.049	961	0.850

^a^: Genes with experiment evidence; ^b^: Genes without experiment evidence but have significant relationship with uveitis; ^c^: Genes without any evidence.

**Table 3:** Comparison of the RWR-based method and GBA-based method.

Index of Part	RWR-Based Method			GBA-Based Method			
	Recall	Precision	F1-Measure	Best Value of *k*	Recall	Precision	F1-Measure
1	0.172	0.089	0.118	1	0.207	0.061	0.094
2	0.172	0.088	0.116	1	0.207	0.059	0.092
3	0.379	0.177	0.242	3	0.345	0.039	0.069
4	0.310	0.141	0.194	1	0.172	0.052	0.079
5	0.400	0.211	0.276	3	0.500	0.061	0.109

**Table 4:** The pseudo-code of the RWR-based method.

RWR-Based Method
**Input:** Ensembl IDs of uveitis-related genes, a PPI network **Output:** A number of putative uveitis-related genes
Execute the RWR algorithm on the PPI network using the Ensembl IDs of uveitis-related genes as seed nodes, yielding a probability for each gene in the network; genes with probabilities higher than 10^−5^ were selected and called RWR genes; Execute a permutation test, producing the *p*-value for each RWR gene; select RWR genes with *p*-values less than 0.05; the remaining genes were called candidate genes; For each candidate gene, calculate its MIS (cf. Equation (3)) and MFS (cf. Equation (5)); select candidate genes with MISs no less than 900 and MFSs larger than 0.8; Output the remaining candidate genes as the putative uveitis-related genes.

## Supplementary Materials

Supplementary materials can be found at www.mdpi.com/1422-0067/18/5/1045/s1.
