# Supplementary material for: Determination of Genes Related to Uveitis by Utilization of the Random Walk with Restart Algorithm on a Protein–Protein Interaction Network
Source: Int J Mol Sci. 2017 May 13;18(5):1045. doi: 10.3390/ijms18051045 (PMC5454957; doi:10.3390/ijms18051045)
Supplement: Supplementary file 1 [file ijms-18-01045-s001.zip › Supp-I.pdf]

Supplementary Table I. Candidate genes yielded by RWR-based method and their measurements

Ensembl ID	Gene symbol	Probability	P-value	MIS	MFS
ENSP00000328973	TSPO	3.93E-04	<0.001	865	0.684
ENSP00000348986	INS-IGF2	3.70E-04	<0.001	968	0.767
ENSP00000263341	IL1B	3.58E-04	<0.001	999	0.873
ENSP00000360266	JUN	3.22E-04	<0.001	999	0.831
ENSP00000221930	TGFB1	2.90E-04	<0.001	997	0.832
ENSP00000228534	IL23A	2.87E-04	<0.001	998	0.844
ENSP00000296871	CSF2	2.71E-04	<0.001	992	0.875
ENSP00000354394	STAT1	2.63E-04	<0.001	999	0.852
ENSP00000245907	C3	2.51E-04	<0.001	999	0.660
ENSP00000216341	GZMB	2.46E-04	<0.001	991	0.829
ENSP00000369293	IL2RA	2.46E-04	<0.001	999	0.866
ENSP00000364114	HLA-DRB5	2.27E-04	<0.001	948	0.822
ENSP00000356694	FASLG	2.27E-04	<0.001	965	0.795
ENSP00000278385	CD44	2.26E-04	<0.001	986	0.797
ENSP00000260010	TLR2	2.25E-04	<0.001	968	0.888
ENSP00000290866	ACE	2.25E-04	<0.001	874	0.707
ENSP00000294728	VCAM1	2.23E-04	<0.001	968	0.882
ENSP00000356346	PTPRC	2.18E-04	<0.001	994	0.826
ENSP00000264246	CD80	2.18E-04	<0.001	999	0.820
ENSP00000313419	CD19	2.15E-04	<0.001	947	0.837

ENSP00000354901	CXCL9	2.13E-04	<0.001	999	0.883
ENSP00000365435	TNFRSF1B	2.13E-04	<0.001	996	0.794
ENSP00000229239	GAPDH	2.12E-04	<0.001	922	0.824
ENSP00000396688	C4A	2.10E-04	<0.001	812	0.564
ENSP00000375881	ALPP	2.10E-04	<0.001	948	0.752
ENSP00000337825	LCK	2.10E-04	<0.001	999	0.741
ENSP00000318472	NCAM1	2.04E-04	<0.001	958	0.701
ENSP00000295453	ALPPL2	2.02E-04	<0.001	948	0.752
ENSP00000359663	CD40LG	1.97E-04	<0.001	999	0.839
ENSP00000320084	CD276	1.91E-04	<0.001	955	0.823
ENSP00000283635	CD8A	1.91E-04	<0.001	990	0.815
ENSP00000416330	TGFBI	1.91E-04	<0.001	917	0.813
ENSP00000310036	CD34	1.88E-04	<0.001	924	0.794
ENSP00000296545	IL15	1.85E-04	<0.001	946	0.806
ENSP00000386884	CXCR4	1.82E-04	<0.001	995	0.791
ENSP00000263339	IL1A	1.82E-04	<0.001	996	0.820
ENSP00000379625	MYD88	1.82E-04	<0.001	999	0.882
ENSP00000302665	IGF1	1.81E-04	<0.001	947	0.800
ENSP00000274306	GZMA	1.80E-04	<0.001	908	0.718
ENSP00000300134	STAT6	1.77E-04	<0.001	999	0.804
ENSP00000361405	MMP9	1.70E-04	<0.001	971	0.833
ENSP00000379110	CXCL1	1.69E-04	<0.001	973	0.827

ENSP00000229402	KLRB1	1.68E-04	<0.001	677	0.688
ENSP00000259206	IL1RN	1.68E-04	<0.001	999	0.836
ENSP00000351671	CCL20	1.65E-04	<0.001	999	0.841
ENSP00000250151	CCL4	1.64E-04	<0.001	994	0.820
ENSP00000287497	ITGAM	1.63E-04	<0.001	999	0.793
ENSP00000372815	C4A	1.62E-04	<0.001	565	0.564
ENSP00000227752	IL10RA	1.62E-04	<0.001	999	0.770
ENSP00000263321	TYR	1.61E-04	<0.001	659	0.731
ENSP00000410046	HLA-DRB4	1.61E-04	<0.001	669	0.000
ENSP00000263967	PIK3CA	1.59E-04	<0.001	962	0.765
ENSP00000416022	HLA-DRB3	1.58E-04	<0.001	750	0.000
ENSP00000337127	SOD2	1.56E-04	<0.001	958	0.657
ENSP00000353874	TLR9	1.55E-04	<0.001	958	0.854
ENSP00000225474	CSF3	1.55E-04	<0.001	916	0.829
ENSP00000223642	C5	1.55E-04	<0.001	906	0.518
ENSP00000229794	MAPK14	1.53E-04	<0.001	963	0.796
ENSP00000415941	C4B	1.53E-04	<0.001	893	0.376
ENSP00000252486	APOE	1.52E-04	<0.001	927	0.765
ENSP00000364444	C4A	1.51E-04	<0.001	609	0.564
ENSP00000286758	CXCL13	1.49E-04	<0.001	986	0.806
ENSP00000245414	IRF1	1.48E-04	<0.001	992	0.728
ENSP00000365048	TNFSF13B	1.48E-04	<0.001	931	0.747

ENSP00000314425	IL12RB1	1.48E-04	<0.001	993	0.772
ENSP00000412786	C4B_2	1.47E-04	<0.001	565	0.448
ENSP00000388662	C4A	1.46E-04	<0.001	567	0.564
ENSP00000327251	NOS2	1.46E-04	<0.001	973	0.735
ENSP00000331736	SELE	1.46E-04	<0.001	978	0.830
ENSP00000336769	KIR2DL1	1.45E-04	<0.001	999	0.552
ENSP00000226279	CD38	1.43E-04	<0.001	981	0.774
ENSP00000352121	PIK3CG	1.41E-04	<0.001	930	0.777
ENSP00000368450	CD83	1.40E-04	<0.001	927	0.787
ENSP00000329411	IRF7	1.38E-04	<0.001	925	0.738
ENSP00000349252	ITGAL	1.37E-04	<0.001	999	0.740
ENSP00000259607	CCL21	1.36E-04	<0.001	999	0.798
ENSP00000375608	KIR3DL1	1.35E-04	<0.001	988	0.542
ENSP00000354566	CD3E	1.34E-04	<0.001	968	0.735
ENSP00000358501	CD58	1.32E-04	<0.001	929	0.701
ENSP00000304915	IL13	1.31E-04	<0.001	999	0.813
ENSP00000410857	HLA-DRB4	1.30E-04	<0.001	673	0.768
ENSP00000225245	CCL3	1.29E-04	<0.001	997	0.757
ENSP00000338130	KLRD1	1.29E-04	<0.001	902	0.593
ENSP00000263686	SELP	1.28E-04	<0.001	930	0.743
ENSP00000306884	CXCL11	1.28E-04	<0.001	999	0.818
ENSP00000296028	PPBP	1.28E-04	<0.001	948	0.789

ENSP00000369554	IFNA2	1.27E-04	<0.001	972	0.717
ENSP00000225844	CCL13	1.27E-04	<0.001	994	0.779
ENSP00000274520	IL9	1.27E-04	<0.001	965	0.806
ENSP00000358490	CD2	1.26E-04	<0.001	837	0.591
ENSP00000370034	TLR7	1.26E-04	<0.001	926	0.819
ENSP00000216223	IL2RB	1.24E-04	<0.001	999	0.663
ENSP00000276927	IFNA1	1.22E-04	<0.001	899	0.740
ENSP00000302234	CCL11	1.22E-04	<0.001	996	0.720
ENSP00000289153	PIK3CB	1.21E-04	<0.001	963	0.784
ENSP00000259271	GAD2	1.20E-04	<0.001	563	0.322
ENSP00000300692	CD3D	1.20E-04	<0.001	938	0.609
ENSP00000347197	C5AR1	1.19E-04	<0.001	933	0.774
ENSP00000366563	PIK3CD	1.18E-04	<0.001	921	0.757
ENSP00000308815	CCL19	1.17E-04	<0.001	998	0.792
ENSP00000291890	NCR1	1.16E-04	<0.001	540	0.594
ENSP00000357470	IL6R	1.16E-04	<0.001	999	0.792
ENSP00000370343	IRF4	1.13E-04	<0.001	917	0.711
ENSP00000348273	MBP	1.13E-04	<0.001	752	0.652
ENSP00000329418	SOCS1	1.13E-04	<0.001	999	0.732
ENSP00000298125	WDFY2	1.11E-04	<0.001	562	0.747
ENSP00000296027	CXCL5	1.11E-04	<0.001	958	0.811
ENSP00000377141	ARRB1	1.10E-04	<0.001	950	0.409

ENSP00000411940	IFNA17	1.10E-04	<0.001	894	0.711
ENSP00000369574	IFNA21	1.10E-04	<0.001	894	0.720
ENSP00000390707	HLA-E	1.09E-04	<0.001	556	0.707
ENSP00000353472	HLA-G	1.08E-04	<0.001	908	0.647
ENSP00000308165	CD36	1.07E-04	<0.001	914	0.728
ENSP00000352064	KLRC1	1.06E-04	<0.001	907	0.568
ENSP00000209873	AAAS	1.06E-04	<0.001	346	0.135
ENSP00000395005	MOG	1.06E-04	<0.001	926	0.646
ENSP00000391898	MOG	1.06E-04	<0.001	926	0.646
ENSP00000390785	MOG	1.06E-04	<0.001	926	0.646
ENSP00000366095	MOG	1.05E-04	<0.001	926	0.646
ENSP00000357025	CD48	1.05E-04	<0.001	845	0.671
ENSP00000004921	CCL18	1.05E-04	<0.001	921	0.738
ENSP00000296029	PF4	1.04E-04	<0.001	981	0.671
ENSP00000240618	KLRK1	1.03E-04	<0.001	461	0.526
ENSP00000299855	MMP3	1.03E-04	<0.001	894	0.717
ENSP00000319635	CXCR2	1.02E-04	<0.001	999	0.851
ENSP00000293778	CXCL16	1.02E-04	<0.001	952	0.800
ENSP00000342300	NELFCD	1.02E-04	<0.001	788	0.722
ENSP00000354932	TLR1	9.99E-05	<0.001	804	0.653
ENSP00000252723	EPO	9.98E-05	<0.001	963	0.749
ENSP00000362994	TRAF1	9.94E-05	<0.001	998	0.727

ENSP00000268296	ITGAX	9.79E-05	<0.001	970	0.674
ENSP00000200307	CCL7	9.79E-05	<0.001	988	0.686
ENSP00000264563	IL11	9.69E-05	<0.001	965	0.766
ENSP00000303276	RNASE2	9.66E-05	<0.001	430	0.589
ENSP00000160262	ICAM3	9.66E-05	<0.001	905	0.732
ENSP00000348019	SLC17A5	9.57E-05	<0.001	307	0.517
ENSP00000375086	CCL25	9.51E-05	<0.001	958	0.743
ENSP00000323074	RAI1	9.49E-05	<0.001	428	0.150
ENSP00000338010	IL21R	9.48E-05	<0.001	999	0.775
ENSP00000393646	HLA-DMB	9.47E-05	<0.001	852	0.681
ENSP00000296026	CXCL3	9.47E-05	<0.001	946	0.755
ENSP00000325525	KIR3DL2	9.46E-05	<0.001	962	0.459
ENSP00000416509	LTA	9.36E-05	<0.001	645	0.701
ENSP00000352672	PGLYRP4	9.31E-05	<0.001	722	0.721
ENSP00000397331	HLA-G	9.28E-05	<0.001	895	0.647
ENSP00000340011	KIR2DS4	9.27E-05	<0.001	961	0.493
ENSP00000409132	HLA-G	9.27E-05	<0.001	900	0.647
ENSP00000221452	RELB	9.23E-05	<0.001	919	0.711
ENSP00000363115	FGR	9.18E-05	<0.001	954	0.654
ENSP00000200691	MT3	9.18E-05	<0.001	611	0.727
ENSP00000292144	CD3G	9.07E-05	<0.001	939	0.574
ENSP00000407674	HLA-DPB1	8.97E-05	<0.001	629	0.734

ENSP00000326432	CCR8	8.90E-05	<0.001	951	0.816
ENSP00000387624	HLA-G	8.88E-05	<0.001	899	0.647
ENSP00000398655	ITK	8.87E-05	<0.001	951	0.652
ENSP00000364076	HLA-DQA2	8.85E-05	<0.001	955	0.726
ENSP00000269280	NLRP1	8.82E-05	<0.001	995	0.623
ENSP00000312262	ADRBK1	8.80E-05	<0.001	988	0.485
ENSP00000312326	AOC3	8.71E-05	<0.001	839	0.665
ENSP00000295683	CXCR1	8.67E-05	<0.001	999	0.833
ENSP00000359504	GBP1	8.62E-05	<0.001	923	0.532
ENSP00000397420	HLA-E	8.53E-05	<0.001	552	0.707
ENSP00000226317	CXCL6	8.49E-05	<0.001	939	0.736
ENSP00000281834	TNFSF4	8.40E-05	<0.001	641	0.688
ENSP00000229134	IL26	8.18E-05	<0.001	675	0.736
ENSP00000296861	TNFRSF21	7.99E-05	<0.001	461	0.352
ENSP00000228438	CLEC2B	7.91E-05	<0.001	506	0.580
ENSP00000247153	CFP	7.86E-05	<0.001	805	0.626
ENSP00000364396	TNXB	7.82E-05	<0.001	758	0.321
ENSP00000316333	CD55	7.70E-05	<0.001	896	0.547
ENSP00000315997	LILRB1	7.59E-05	<0.001	953	0.465
ENSP00000221847	EBI3	7.58E-05	<0.001	932	0.725
ENSP00000349365	IL27	7.51E-05	<0.001	803	0.767
ENSP00000229708	ULBP1	7.41E-05	<0.001	674	0.508

ENSP00000403495	LTA	7.36E-05	<0.001	430	0.701
ENSP00000225842	CCL1	7.35E-05	<0.001	956	0.666
ENSP00000407133	LTA	7.32E-05	<0.001	430	0.701
ENSP00000416337	LTA	7.32E-05	<0.001	430	0.701
ENSP00000360181	SH2D1A	7.21E-05	<0.001	867	0.627
ENSP00000372582	HLA-DPB1	7.15E-05	<0.001	466	0.734
ENSP00000222902	CCL24	7.14E-05	<0.001	988	0.580
ENSP00000349562	CCDC180	6.98E-05	<0.001	462	0.550
ENSP00000290200	IL10RB	6.94E-05	<0.001	986	0.589
ENSP00000356792	XCL1	6.80E-05	<0.001	886	0.597
ENSP00000216968	PROCR	6.77E-05	<0.001	890	0.450
ENSP00000375829	LAD1	6.75E-05	<0.001	516	0.361
ENSP00000263461	WDR11	6.75E-05	<0.001	540	0.239
ENSP00000319883	EMR2	6.74E-05	<0.001	235	0.274
ENSP00000382422	HLA-DPB1	6.72E-05	<0.001	364	0.734
ENSP00000215631	GADD45B	6.72E-05	<0.001	926	0.602
ENSP00000351473	GIMAP5	6.66E-05	<0.001	880	0.716
ENSP00000260356	THBS1	6.62E-05	<0.001	834	0.710
ENSP00000220616	TG	6.62E-05	<0.001	508	0.485
ENSP00000245903	CD70	6.50E-05	<0.001	997	0.711
ENSP00000397587	HLA-DPA1	6.49E-05	<0.001	618	0.380
ENSP00000363330	CD52	6.45E-05	<0.001	541	0.529

ENSP00000393355	MICB	6.39E-05	<0.001	907	0.509
ENSP00000311856	SLC25A30	6.38E-05	<0.001	562	0.773
ENSP00000279544	KLRF1	6.37E-05	<0.001	373	0.525
ENSP00000295400	TGFA	6.37E-05	<0.001	931	0.703
ENSP00000293280	CCL23	6.34E-05	<0.001	983	0.526
ENSP00000407561	MICB	6.33E-05	<0.001	426	0.509
ENSP00000348918	SAA1	6.32E-05	<0.001	806	0.654
ENSP00000383142	NFKBIL1	6.08E-05	<0.001	375	0.414
ENSP00000411611	NFKBIL1	6.07E-05	<0.001	375	0.414
ENSP00000288709	MMEL1	6.07E-05	<0.001	463	0.408
ENSP00000365318	NFKBIL1	6.05E-05	<0.001	375	0.414
ENSP00000366729	TNFRSF9	6.04E-05	<0.001	899	0.502
ENSP00000330374	GIMAP6	6.00E-05	<0.001	922	0.642
ENSP00000332812	PTGDR2	5.89E-05	<0.001	918	0.737
ENSP00000407942	C4B	5.86E-05	<0.001	370	0.376
ENSP00000345008	FBLN5	5.84E-05	<0.001	429	0.261
ENSP00000302833	GIMAP1	5.79E-05	<0.001	793	0.594
ENSP00000245817	TNFSF9	5.75E-05	<0.001	874	0.697
ENSP00000395544	NFKBIL1	5.62E-05	<0.001	297	0.414
ENSP00000394486	NFKBIL1	5.62E-05	<0.001	297	0.414
ENSP00000235933	CD160	5.59E-05	<0.001	857	0.491
ENSP00000398890	HLA-DMB	5.47E-05	<0.001	613	0.681

ENSP00000354575	LSR	5.44E-05	<0.001	750	0.598
ENSP00000320709	ADIPOQ	5.20E-05	<0.001	909	0.423
ENSP00000357152	CD1C	4.93E-05	<0.001	657	0.656
ENSP00000264952	GRK7	4.90E-05	<0.001	800	0.125
ENSP00000281631	PARP8	4.83E-05	<0.001	183	0.199
ENSP00000252229	MICB	4.74E-05	<0.001	673	0.509
ENSP00000393566	HLA-DPA1	4.70E-05	<0.001	764	0.380
ENSP00000349709	RAET1E	4.60E-05	<0.001	417	0.332
ENSP00000356552	MR1	4.55E-05	<0.001	552	0.558
ENSP00000306523	INSM2	4.43E-05	<0.001	563	0.139
ENSP00000243347	TNFAIP6	4.43E-05	<0.001	517	0.322
ENSP00000324742	IL32	4.20E-05	<0.001	877	0.601
ENSP00000356329	RAET1G	4.14E-05	<0.001	401	0.431
ENSP00000349893	DLK2	4.06E-05	<0.001	407	0.207
ENSP00000337432	IL17F	3.62E-05	<0.001	914	0.593
ENSP00000265598	LAMP3	3.61E-05	<0.001	611	0.615
ENSP00000344095	ZNF683	1.75E-05	<0.001	657	0.379
ENSP00000162749	TNFRSF1A	2.30E-04	0.001	999	0.826
ENSP00000316328	CIITA	1.70E-04	0.001	933	0.659
ENSP00000330341	SOCS3	1.50E-04	0.001	999	0.786
ENSP00000342215	KIR2DL3	1.49E-04	0.001	999	0.526
ENSP00000313967	C1QB	1.42E-04	0.001	902	0.598

ENSP00000383645	KIR3DS1	1.35E-04	0.001	966	0.572
ENSP00000219070	MMP2	1.23E-04	0.001	940	0.733
ENSP00000343204	JAK1	1.21E-04	0.001	999	0.818
ENSP00000262407	ITGA2B	1.16E-04	0.001	825	0.496
ENSP00000225275	MPO	1.14E-04	0.001	922	0.766
ENSP00000292174	CXCR5	1.14E-04	0.001	976	0.820
ENSP00000189444	NFKB2	1.07E-04	0.001	922	0.636
ENSP00000356946	FCGR3A	1.07E-04	0.001	867	0.701
ENSP00000369581	IFNB1	1.04E-04	0.001	927	0.684
ENSP00000372793	LTA	9.43E-05	0.001	647	0.701
ENSP00000357757	LCE1C	9.11E-05	0.001	750	0.931
ENSP00000357776	LCE3D	9.00E-05	0.001	889	0.529
ENSP00000352071	CD163	8.66E-05	0.001	912	0.629
ENSP00000402134	MICA	8.49E-05	0.001	821	0.463
ENSP00000241261	TNFSF10	8.28E-05	0.001	750	0.756
ENSP00000243786	INHA	8.23E-05	0.001	987	0.510
ENSP00000339634	KIR2DL4	7.90E-05	0.001	899	0.429
ENSP00000331172	CD8B	7.88E-05	0.001	963	0.581
ENSP00000356429	RGS1	7.84E-05	0.001	934	0.641
ENSP00000261233	IRAK3	7.74E-05	0.001	961	0.675
ENSP00000282397	FLT1	7.69E-05	0.001	999	0.711
ENSP00000359497	GBP2	7.36E-05	0.001	915	0.512

ENSP00000216117	HMOX1	7.31E-05	0.001	931	0.644
ENSP00000241453	FLT3	7.14E-05	0.001	885	0.756
ENSP00000330138	SSTR3	7.00E-05	0.001	901	0.458
ENSP00000293275	CCL16	6.95E-05	0.001	998	0.678
ENSP00000371327	KLRC2	6.75E-05	0.001	851	0.452
ENSP00000354416	CCL28	6.24E-05	0.001	967	0.617
ENSP00000337065	CXCL14	6.23E-05	0.001	859	0.563
ENSP00000376445	TIRAP	6.16E-05	0.001	999	0.749
ENSP00000377204	GRK6	5.80E-05	0.001	962	0.271
ENSP00000309124	OLR1	5.78E-05	0.001	901	0.566
ENSP00000332116	PDE4B	5.71E-05	0.001	813	0.159
ENSP00000306565	ISG20	5.69E-05	0.001	929	0.536
ENSP00000261652	TNFRSF13B	5.65E-05	0.001	540	0.632
ENSP00000319476	ICOS	5.08E-05	0.001	886	0.715
ENSP00000226524	PF4V1	5.01E-05	0.001	860	0.242
ENSP00000355812	FGFR1OP	4.90E-05	0.001	899	0.125
ENSP00000408146	HLA-DPB1	4.89E-05	0.001	715	0.734
ENSP00000216160	TAB1	4.74E-05	0.001	999	0.642
ENSP00000302935	IL16	4.35E-05	0.001	983	0.573
ENSP00000286380	RAET1L	3.24E-05	0.001	495	0.427
ENSP00000373006	MICB	3.09E-05	0.001	324	0.509
ENSP00000407092	MICB	2.95E-05	0.001	324	0.509

ENSP00000293218	UNK	2.66E-05	0.001	335	0.206
ENSP00000412031	HLA-DRB4	1.68E-04	0.002	916	0.000
ENSP00000296795	TLR3	1.58E-04	0.002	966	0.858
ENSP00000363773	C1QA	1.57E-04	0.002	965	0.535
ENSP00000372607	HLA-DRB3	1.56E-04	0.002	878	0.000
ENSP00000375844	CAPN10	1.32E-04	0.002	724	0.256
ENSP00000357721	S100A8	1.26E-04	0.002	943	0.588
ENSP00000414196	HLA-DPB1	1.24E-04	0.002	907	0.734
ENSP00000273550	FTH1	1.16E-04	0.002	907	0.615
ENSP00000342681	CD5	1.11E-04	0.002	945	0.762
ENSP00000356033	TAGAP	1.10E-04	0.002	593	0.504
ENSP00000315477	CD209	9.39E-05	0.002	904	0.737
ENSP00000304414	CXCR6	9.27E-05	0.002	964	0.803
ENSP00000315768	STAT2	9.06E-05	0.002	998	0.678
ENSP00000363079	MBL2	9.06E-05	0.002	747	0.586
ENSP00000340089	TLR5	8.44E-05	0.002	857	0.795
ENSP00000377218	IRF2	8.14E-05	0.002	917	0.655
ENSP00000302079	C3AR1	8.12E-05	0.002	942	0.723
ENSP00000291582	AIRE	7.73E-05	0.002	515	0.420
ENSP00000381599	MX1	7.35E-05	0.002	927	0.601
ENSP00000335006	LCE3A	7.07E-05	0.002	540	0.426
ENSP00000337383	NLRP3	6.46E-05	0.002	955	0.743

ENSP00000366307	THBD	6.42E-05	0.002	973	0.642
ENSP00000333639	IFNL2	6.25E-05	0.002	806	0.488
ENSP00000354612	PTGS1	6.04E-05	0.002	851	0.608
ENSP00000343505	TNFSF13	5.97E-05	0.002	401	0.646
ENSP00000358092	PRDM1	5.78E-05	0.002	463	0.485
ENSP00000404533	HLA-DRA	5.62E-05	0.002	728	0.654
ENSP00000282141	CRYGC	5.41E-05	0.002	659	0.615
ENSP00000261745	NAA25	5.13E-05	0.002	462	0.252
ENSP00000286639	BATF	5.03E-05	0.002	669	0.379
ENSP00000371376	TLR6	5.01E-05	0.002	609	0.771
ENSP00000265421	POLB	4.49E-05	0.002	294	0.173
ENSP00000264260	IL18RAP	4.47E-05	0.002	996	0.470
ENSP00000324827	AKAP17A	4.31E-05	0.002	317	0.395
ENSP00000218721	MLNR	4.03E-05	0.002	899	0.546
ENSP00000300305	RUNX1	2.14E-04	0.003	963	0.674
ENSP00000219235	CCL22	1.24E-04	0.003	983	0.795
ENSP00000363958	BRD2	1.19E-04	0.003	859	0.456
ENSP00000334869	LCE1A	9.51E-05	0.003	730	0.866
ENSP00000270357	RNPEPL1	8.90E-05	0.003	462	0.302
ENSP00000328207	TNFRSF18	7.31E-05	0.003	942	0.680
ENSP00000343040	HMGB1	7.19E-05	0.003	970	0.585
ENSP00000262158	SMAD7	7.16E-05	0.003	899	0.754

ENSP00000351497	FCGR2B	7.14E-05	0.003	872	0.674
ENSP00000357980	HTRA1	7.06E-05	0.003	430	0.367
ENSP00000229281	C12orf57	7.05E-05	0.003	917	0.323
ENSP00000255409	CHI3L1	6.84E-05	0.003	893	0.391
ENSP00000376609	GRK5	6.39E-05	0.003	899	0.474
ENSP00000372817	STK19	6.38E-05	0.003	401	0.191
ENSP00000246551	HCST	6.33E-05	0.003	585	0.631
ENSP00000264870	F13A1	6.25E-05	0.003	942	0.450
ENSP00000392398	GPX5	6.09E-05	0.003	922	0.479
ENSP00000364482	STK19	6.06E-05	0.003	649	0.191
ENSP00000417404	HFE	5.72E-05	0.003	563	0.295
ENSP00000405295	HLA-DRA	5.67E-05	0.003	782	0.654
ENSP00000316460	FYB	5.65E-05	0.003	929	0.544
ENSP00000380855	PDCD1LG2	4.68E-05	0.003	970	0.637
ENSP00000358831	ELOVL4	4.55E-05	0.003	530	0.074
ENSP00000347596	EFEMP1	4.46E-05	0.003	390	0.214
ENSP00000349477	KLRG1	4.35E-05	0.003	833	0.445
ENSP00000408534	TNFAIP8	3.42E-05	0.003	904	0.176
ENSP00000391592	PTPN6	1.32E-04	0.004	998	0.657
ENSP00000330382	PDGFB	1.31E-04	0.004	956	0.744
ENSP00000357727	S100A9	1.18E-04	0.004	939	0.664
ENSP00000265023	KNG1	1.15E-04	0.004	941	0.614

ENSP00000231454	IL5	1.13E-04	0.004	991	0.803
ENSP00000328173	C1S	9.76E-05	0.004	929	0.558
ENSP00000263408	C9	9.37E-05	0.004	414	0.457
ENSP00000223095	SERPINE1	9.33E-05	0.004	961	0.713
ENSP00000364802	HSPA1A	8.87E-05	0.004	766	0.603
ENSP00000357013	CD244	7.92E-05	0.004	379	0.468
ENSP00000370473	IGFBP3	7.90E-05	0.004	910	0.701
ENSP00000222823	NOD1	7.72E-05	0.004	991	0.866
ENSP00000362299	ENG	7.33E-05	0.004	732	0.612
ENSP00000271588	HMCN1	7.28E-05	0.004	502	0.243
ENSP00000367851	CYBB	7.26E-05	0.004	817	0.530
ENSP00000247829	TSPAN8	6.06E-05	0.004	363	0.386
ENSP00000392762	DCT	5.73E-05	0.004	613	0.578
ENSP00000293328	STAT5B	5.60E-05	0.004	999	0.668
ENSP00000203629	LAG3	5.59E-05	0.004	567	0.604
ENSP00000373024	C6orf15	5.50E-05	0.004	899	0.261
ENSP00000222002	SULT2A1	4.98E-05	0.004	403	0.371
ENSP00000357022	SLAMF7	4.59E-05	0.004	500	0.312
ENSP00000327890	IL3RA	4.55E-05	0.004	972	0.676
ENSP00000409910	HLA-E	4.51E-05	0.004	800	0.707
ENSP00000305107	GIMAP8	4.34E-05	0.004	828	0.434
ENSP00000014930	HEBP1	3.30E-05	0.004	902	0.512

ENSP00000259205	IL36G	2.25E-05	0.004	269	0.439
ENSP00000408697	NUGGC	1.03E-05	0.004	204	0.371
ENSP00000231449	IL4	2.55E-04	0.005	999	0.852
ENSP00000357981	CTSS	1.68E-04	0.005	972	0.720
ENSP00000340944	PTPN11	1.48E-04	0.005	999	0.664
ENSP00000250092	CD68	1.30E-04	0.005	753	0.753
ENSP00000367605	CCL3L3	1.24E-04	0.005	995	0.458
ENSP00000200652	SLC22A4	1.05E-04	0.005	540	0.414
ENSP00000333298	LAMP1	1.01E-04	0.005	812	0.426
ENSP00000264896	SCARB2	9.30E-05	0.005	906	0.733
ENSP00000373091	HLA-E	9.02E-05	0.005	784	0.707
ENSP00000263464	BIRC3	8.92E-05	0.005	996	0.672
ENSP00000419945	ERVW-1	8.27E-05	0.005	969	0.567
ENSP00000283977	PGM3	8.11E-05	0.005	462	0.123
ENSP00000231009	GZMK	8.00E-05	0.005	767	0.658
ENSP00000263923	KDR	7.93E-05	0.005	957	0.703
ENSP00000339151	IKBKB	7.81E-05	0.005	986	0.639
ENSP00000296585	ITGA2	7.33E-05	0.005	852	0.582
ENSP00000409151	HSPA1L	6.51E-05	0.005	396	0.272
ENSP00000306190	SLAMF1	6.42E-05	0.005	458	0.583
ENSP00000248071	KLF2	6.20E-05	0.005	466	0.540
ENSP00000234347	PRTN3	5.84E-05	0.005	804	0.593

ENSP00000260227	MMP7	5.61E-05	0.005	884	0.657
ENSP00000329869	TPO	5.17E-05	0.005	424	0.357
ENSP00000406495	TNXB	4.74E-05	0.005	368	0.321
ENSP00000387438	TNXB	4.72E-05	0.005	368	0.321
ENSP00000389677	TNXB	4.72E-05	0.005	368	0.321
ENSP00000409159	HLA-DQB2	4.71E-05	0.005	817	0.294
ENSP00000308479	GPR171	4.69E-05	0.005	577	0.571
ENSP00000369312	IL15RA	3.60E-05	0.005	809	0.593
ENSP00000397073	TRIM10	3.14E-05	0.005	296	0.373
ENSP00000332766	NPB	3.07E-05	0.005	899	0.484
ENSP00000326407	CLEC1A	2.64E-05	0.005	625	0.477
ENSP00000313437	MMP19	2.51E-05	0.005	859	0.162
ENSP00000226574	NFKB1	1.88E-04	0.006	981	0.717
ENSP00000363548	CXCL12	1.74E-04	0.006	986	0.758
ENSP00000351697	REV3L	1.53E-04	0.006	661	0.710
ENSP00000391676	JAK3	1.52E-04	0.006	997	0.764
ENSP00000354782	CD247	1.20E-04	0.006	951	0.623
ENSP00000306697	MRAP	1.09E-04	0.006	849	0.492
ENSP00000388001	OAS1	1.02E-04	0.006	923	0.621
ENSP00000356016	CR1	9.62E-05	0.006	871	0.585
ENSP00000372746	HLA-DRA	9.50E-05	0.006	952	0.654
ENSP00000261693	SCARB1	9.03E-05	0.006	906	0.719

ENSP00000227758	BIRC2	8.26E-05	0.006	999	0.603
ENSP00000294800	FCGR3B	7.77E-05	0.006	430	0.512
ENSP00000302707	FPR1	7.70E-05	0.006	933	0.702
ENSP00000356954	CTGF	7.07E-05	0.006	914	0.757
ENSP00000394624	OPRM1	6.94E-05	0.006	908	0.496
ENSP00000293276	CCL15	6.88E-05	0.006	966	0.379
ENSP00000301838	FADD	6.78E-05	0.006	977	0.725
ENSP00000263379	IL27RA	6.62E-05	0.006	942	0.671
ENSP00000378130	CFI	6.42E-05	0.006	992	0.478
ENSP00000273430	AGTR1	5.69E-05	0.006	899	0.557
ENSP00000356793	XCL2	5.59E-05	0.006	834	0.618
ENSP00000360973	AGTR2	5.14E-05	0.006	900	0.480
ENSP00000334876	GRK1	5.01E-05	0.006	824	0.121
ENSP00000263087	ITGAE	3.98E-05	0.006	563	0.487
ENSP00000307722	NSG2	3.90E-05	0.006	514	0.000
ENSP00000340125	PPP1R17	3.74E-05	0.006	229	0.096
ENSP00000345676	1-Mar	3.74E-05	0.006	864	0.643
ENSP00000386378	CD207	3.09E-05	0.006	517	0.620
ENSP00000363965	ALPL	1.96E-04	0.007	942	0.749
ENSP00000216797	NFKBIA	1.31E-04	0.007	982	0.757
ENSP00000364898	SYK	1.29E-04	0.007	983	0.739
ENSP00000389288	HLA-DPB1	1.24E-04	0.007	669	0.734

ENSP00000252444	LDLR	1.03E-04	0.007	894	0.667
ENSP00000339804	TSLP	9.67E-05	0.007	962	0.772
ENSP00000290722	PGLYRP3	9.37E-05	0.007	722	0.778
ENSP00000268638	IRF8	8.33E-05	0.007	915	0.661
ENSP00000233957	IL18R1	8.21E-05	0.007	983	0.725
ENSP00000333358	RNASE10	7.38E-05	0.007	262	0.258
ENSP00000271450	FCGR2A	7.25E-05	0.007	978	0.591
ENSP00000218032	TLR8	6.69E-05	0.007	877	0.756
ENSP00000345984	TRAF3IP2	6.63E-05	0.007	663	0.305
ENSP00000279281	VPS51	6.62E-05	0.007	399	0.054
ENSP00000221515	RETN	6.23E-05	0.007	942	0.619
ENSP00000261937	FLT4	6.06E-05	0.007	957	0.694
ENSP00000340210	CD59	5.95E-05	0.007	851	0.453
ENSP00000360869	IFIT1	5.71E-05	0.007	922	0.595
ENSP00000378723	HLA-DMB	5.65E-05	0.007	834	0.681
ENSP00000322788	MMP1	5.62E-05	0.007	953	0.713
ENSP00000327850	NFATC1	5.33E-05	0.007	985	0.599
ENSP00000406872	HLA-DQB2	4.74E-05	0.007	817	0.294
ENSP00000272928	CXCR7	4.02E-05	0.007	927	0.643
ENSP00000288840	SMAD6	3.76E-05	0.007	899	0.553
ENSP00000308925	TLR10	3.28E-05	0.007	828	0.703
ENSP00000298527	CLEC1B	3.04E-05	0.007	657	0.406

ENSP00000303242	ITGB2	1.35E-04	0.008	999	0.727
ENSP00000249075	LIF	1.26E-04	0.008	962	0.793
ENSP00000264606	HDAC4	1.13E-04	0.008	739	0.443
ENSP00000263863	GNLY	9.83E-05	0.008	838	0.568
ENSP00000338018	HIF1A	9.56E-05	0.008	957	0.749
ENSP00000375629	LILRB2	9.38E-05	0.008	946	0.688
ENSP00000333685	MAPK11	8.07E-05	0.008	936	0.638
ENSP00000336829	FGG	8.02E-05	0.008	947	0.525
ENSP00000405533	CCL4L1	7.86E-05	0.008	886	0.629
ENSP00000259089	BLK	7.26E-05	0.008	899	0.485
ENSP00000263621	ELANE	7.25E-05	0.008	946	0.676
ENSP00000046794	LCP2	6.87E-05	0.008	951	0.585
ENSP00000396486	HSPA1L	6.53E-05	0.008	396	0.272
ENSP00000365016	IRS2	6.42E-05	0.008	983	0.577
ENSP00000370842	IL33	5.56E-05	0.008	929	0.728
ENSP00000342278	OAS2	5.48E-05	0.008	910	0.466
ENSP00000252506	GADD45G	5.35E-05	0.008	906	0.310
ENSP00000297350	TNFRSF11B	5.13E-05	0.008	867	0.662
ENSP00000363157	TNFSF15	5.01E-05	0.008	565	0.614
ENSP00000314299	CFHR1	4.99E-05	0.008	902	0.357
ENSP00000353452	MYLK	4.82E-05	0.008	911	0.510
ENSP00000364016	PSMB8	4.72E-05	0.008	318	0.321

ENSP00000411321	HLA-DMB	3.98E-05	0.008	899	0.681
ENSP00000360195	KANK4	3.90E-05	0.008	899	0.159
ENSP00000313309	FUZ	3.82E-05	0.008	219	0.162
ENSP00000355870	HLX	3.17E-05	0.008	939	0.473
ENSP00000353500	CLEC4C	2.99E-05	0.008	567	0.604
ENSP00000364028	ECE1	2.03E-05	0.008	363	0.390
ENSP00000356438	PTGS2	1.92E-04	0.009	972	0.864
ENSP00000402694	HLA-E	1.09E-04	0.009	896	0.707
ENSP00000265132	AMBP	1.04E-04	0.009	519	0.404
ENSP00000308620	RAG2	9.98E-05	0.009	784	0.761
ENSP00000334145	F3	8.36E-05	0.009	947	0.722
ENSP00000395701	TAPBP	7.89E-05	0.009	882	0.280
ENSP00000301974	PTAFR	7.78E-05	0.009	954	0.651
ENSP00000284818	LY96	6.81E-05	0.009	999	0.730
ENSP00000288602	BRAF	6.57E-05	0.009	575	0.391
ENSP00000372608	HLA-DRA	6.14E-05	0.009	982	0.654
ENSP00000356087	IKBKE	5.80E-05	0.009	985	0.570
ENSP00000356320	ULBP2	5.54E-05	0.009	817	0.479
ENSP00000409197	CCL14	5.53E-05	0.009	981	0.314
ENSP00000334122	FGF3	5.27E-05	0.009	463	0.612
ENSP00000306185	ANTXR2	4.55E-05	0.009	550	0.461
ENSP00000330659	SOCS7	3.96E-05	0.009	823	0.353

ENSP00000263636	LY75	3.89E-05	0.009	500	0.660
ENSP00000264316	TXK	3.44E-05	0.009	675	0.559
ENSP00000219244	CCL17	1.20E-04	0.01	984	0.808
ENSP00000348170	HP	1.16E-04	0.01	926	0.600
ENSP00000248594	PTPN12	8.83E-05	0.01	954	0.584
ENSP00000259631	CCL27	7.89E-05	0.01	950	0.704
ENSP00000224784	ACTA2	7.77E-05	0.01	687	0.616
ENSP00000380073	IRF9	6.54E-05	0.01	963	0.607
ENSP00000391798	STK19	6.33E-05	0.01	401	0.191
ENSP00000371328	KLRC3	6.31E-05	0.01	829	0.420
ENSP00000350467	NAT9	6.10E-05	0.01	409	0.266
ENSP00000248598	FGL2	6.06E-05	0.01	835	0.692
ENSP00000357986	PLEKHA1	6.02E-05	0.01	657	0.246
ENSP00000357103	DARC	5.92E-05	0.01	981	0.515
ENSP00000239849	TNFSF11	5.55E-05	0.01	927	0.625
ENSP00000380785	HOOK2	4.69E-05	0.01	425	0.220
ENSP00000402951	HLA-DRA	4.45E-05	0.01	917	0.654
ENSP00000308361	P2RY14	4.45E-05	0.01	921	0.473
ENSP00000317891	TNIP1	4.21E-05	0.01	997	0.477
ENSP00000377783	PROS1	4.20E-05	0.01	928	0.517
ENSP00000378195	GPR75	4.18E-05	0.01	271	0.262
ENSP00000302855	ZNF280A	3.59E-05	0.01	283	0.439

ENSP00000329212	ATF7	3.44E-05	0.01	921	0.209
ENSP00000356024	CR2	3.31E-05	0.01	340	0.497
ENSP00000396656	NFKBIL1	3.09E-05	0.01	899	0.414
ENSP00000415805	AIF1	2.73E-05	0.01	462	0.446
ENSP00000239761	MRC1	2.47E-05	0.01	307	0.399
ENSP00000372912	LY6G6F	1.24E-05	0.01	210	0.280
ENSP00000295025	REL	1.26E-04	0.011	936	0.761
ENSP00000278407	SERPING1	1.21E-04	0.011	918	0.562
ENSP00000297494	NOS3	1.06E-04	0.011	944	0.724
ENSP00000302150	PRL	1.01E-04	0.011	994	0.684
ENSP00000010338	TRAF3IP3	1.00E-04	0.011	899	0.368
ENSP00000266646	INHBE	8.24E-05	0.011	829	0.737
ENSP00000229022	VDR	8.11E-05	0.011	540	0.668
ENSP00000368632	GATA3	6.95E-05	0.011	944	0.585
ENSP00000345492	SH2B3	6.01E-05	0.011	985	0.306
ENSP00000356056	DYNLT1	5.94E-05	0.011	565	0.226
ENSP00000323280	CD6	5.65E-05	0.011	625	0.529
ENSP00000339428	SOCS2	5.56E-05	0.011	962	0.630
ENSP00000302569	CLEC7A	5.54E-05	0.011	657	0.603
ENSP00000314173	SAMD12	5.01E-05	0.011	513	0.235
ENSP00000372721	PSMB9	4.88E-05	0.011	657	0.293
ENSP00000260049	IL18BP	4.80E-05	0.011	984	0.702

ENSP00000332139	CFD	4.54E-05	0.011	899	0.538
ENSP00000320936	IL17RA	4.39E-05	0.011	993	0.641
ENSP00000256452	IL5RA	3.51E-05	0.011	995	0.593
ENSP00000188790	FAP	3.39E-05	0.011	423	0.122
ENSP00000244360	RNF39	3.17E-05	0.011	385	0.279
ENSP00000316737	LCE1D	3.16E-05	0.011	513	0.382
ENSP00000333656	SIGIRR	2.88E-05	0.011	738	0.643
ENSP00000354581	GPX6	2.63E-05	0.011	284	0.214
ENSP00000387930	RNF39	2.60E-05	0.011	273	0.279
ENSP00000356713	IFNGR1	1.03E-04	0.012	999	0.740
ENSP00000355627	AGT	9.51E-05	0.012	896	0.652
ENSP00000261942	FAF2	9.12E-05	0.012	619	0.099
ENSP00000215637	MADCAM1	8.95E-05	0.012	909	0.676
ENSP00000220751	RIPK2	8.14E-05	0.012	999	0.759
ENSP00000369820	PIGA	6.97E-05	0.012	514	0.254
ENSP00000311857	PTPN2	6.76E-05	0.012	978	0.456
ENSP00000263388	NOTCH3	6.24E-05	0.012	994	0.799
ENSP00000227251	CRYAB	5.80E-05	0.012	822	0.393
ENSP00000375872	ATG16L1	5.58E-05	0.012	953	0.589
ENSP00000290524	RFX5	5.08E-05	0.012	318	0.222
ENSP00000320509	BANK1	5.06E-05	0.012	578	0.489
ENSP00000290219	IFNGR2	4.92E-05	0.012	990	0.671

ENSP00000363869	VSIG4	4.57E-05	0.012	552	0.381
ENSP00000267015	GPR84	4.54E-05	0.012	272	0.275
ENSP00000357038	SLAMF6	4.52E-05	0.012	374	0.469
ENSP00000320376	P2RY13	4.14E-05	0.012	921	0.616
ENSP00000350005	GIP	4.01E-05	0.012	227	0.305
ENSP00000401149	TAP1	3.95E-05	0.012	297	0.347
ENSP00000313644	MAP4K4	3.91E-05	0.012	884	0.359
ENSP00000378288	MYLK3	3.87E-05	0.012	850	0.274
ENSP00000256649	TRIM45	3.74E-05	0.012	461	0.324
ENSP00000353586	ZNF280B	3.73E-05	0.012	290	0.464
ENSP00000407291	RNF39	3.29E-05	0.012	401	0.279
ENSP00000408539	RNF39	3.29E-05	0.012	401	0.279
ENSP00000294671	GBP7	3.27E-05	0.012	509	0.197
ENSP00000415480	TRIM15	3.01E-05	0.012	288	0.353
ENSP00000393693	LTB	2.94E-05	0.012	340	0.503
ENSP00000358783	NPBWR2	2.87E-05	0.012	899	0.535
ENSP00000072516	IL1RAP	2.70E-05	0.012	996	0.293
ENSP00000204637	FLT3LG	2.67E-05	0.012	418	0.414
ENSP00000258449	TGFBRAP1	2.57E-05	0.012	899	0.233
ENSP00000296513	ADAD1	2.41E-05	0.012	366	0.290
ENSP00000373079	ATAT1	2.37E-05	0.012	899	0.346
ENSP00000349929	SDCCAG3	2.26E-05	0.012	362	0.472

ENSP00000311300	CTSW	2.23E-05	0.012	354	0.180
ENSP00000296870	IL3	1.45E-04	0.013	994	0.794
ENSP00000224237	VIM	1.21E-04	0.013	976	0.754
ENSP00000307875	B3GAT1	8.95E-05	0.013	788	0.552
ENSP00000378546	TAPBP	7.94E-05	0.013	886	0.280
ENSP00000369213	DDX58	7.25E-05	0.013	954	0.673
ENSP00000295718	PTPRN	7.19E-05	0.013	507	0.192
ENSP00000226218	SEBOX	7.07E-05	0.013	878	0.643
ENSP00000343023	SP100	6.69E-05	0.013	926	0.384
ENSP00000280193	VEGFC	6.69E-05	0.013	916	0.742
ENSP00000221466	FCGRT	6.50E-05	0.013	441	0.431
ENSP00000384708	FSHR	6.45E-05	0.013	229	0.382
ENSP00000409346	CISH	5.88E-05	0.013	973	0.709
ENSP00000314620	MS4A1	5.69E-05	0.013	796	0.584
ENSP00000402406	PSMB8	5.12E-05	0.013	460	0.321
ENSP00000291860	KIR3DL3	5.07E-05	0.013	859	0.505
ENSP00000409378	CHRM4	4.97E-05	0.013	900	0.451
ENSP00000360281	C8B	4.70E-05	0.013	374	0.387
ENSP00000333657	MX2	4.41E-05	0.013	910	0.504
ENSP00000296511	ANXA5	4.41E-05	0.013	839	0.666
ENSP00000270279	CBLC	4.10E-05	0.013	800	0.382
ENSP00000412310	SKIV2L	3.99E-05	0.013	369	0.162

ENSP00000263409	LIFR	3.88E-05	0.013	948	0.551
ENSP00000372713	HLA-DOA	3.54E-05	0.013	564	0.249
ENSP00000216802	PSME2	3.06E-05	0.013	829	0.366
ENSP00000370979	CRLF2	2.88E-05	0.013	958	0.588
ENSP00000365227	AIF1	2.75E-05	0.013	462	0.446
ENSP00000357231	THEMIS	2.64E-05	0.013	374	0.341
ENSP00000363836	CCNY	2.32E-05	0.013	308	0.417
ENSP00000405838	HLA-DPA1	1.48E-05	0.013	173	0.380
ENSP00000236147	SELL	1.20E-04	0.014	899	0.752
ENSP00000264708	POMC	1.03E-04	0.014	977	0.636
ENSP00000264033	CBL	9.29E-05	0.014	999	0.634
ENSP00000332124	MRC1L1	8.87E-05	0.014	977	0.000
ENSP00000364597	PADI4	8.28E-05	0.014	752	0.387
ENSP00000284240	THY1	7.79E-05	0.014	659	0.503
ENSP00000351602	FUT4	5.79E-05	0.014	655	0.523
ENSP00000284273	UBASH3B	5.41E-05	0.014	713	0.188
ENSP00000333919	BTLA	5.07E-05	0.014	673	0.609
ENSP00000266085	TIMP3	4.84E-05	0.014	430	0.616
ENSP00000293826	TNFSF12-TNFSF13	4.62E-05	0.014	427	0.503
ENSP00000308549	ADORA1	4.37E-05	0.014	903	0.502
ENSP00000252268	DPF2	4.16E-05	0.014	229	0.251
ENSP00000341327	SOCS4	4.10E-05	0.014	938	0.366

ENSP00000350878	S1PR3	3.88E-05	0.014	921	0.574
ENSP00000221978	NKG7	3.56E-05	0.014	702	0.524
ENSP00000270800	IL22RA1	3.54E-05	0.014	999	0.550
ENSP00000353259	CD300A	3.54E-05	0.014	366	0.389
ENSP00000259633	CD72	3.54E-05	0.014	471	0.562
ENSP00000410481	LTB	2.76E-05	0.014	340	0.503
ENSP00000374791	IGKV6-21	2.00E-05	0.014	204	0.401
ENSP00000341803	TCTEX1D4	1.43E-05	0.014	619	0.282
ENSP00000322570	POLE	1.49E-04	0.015	659	0.706
ENSP00000355718	DLL1	1.04E-04	0.015	940	0.763
ENSP00000380227	ITGA4	9.98E-05	0.015	900	0.688
ENSP00000387122	CLEC16A	8.43E-05	0.015	516	0.477
ENSP00000329967	TBK1	8.14E-05	0.015	994	0.610
ENSP00000340396	GBP5	7.73E-05	0.015	776	0.554
ENSP00000304236	CD14	7.53E-05	0.015	986	0.633
ENSP00000399309	NEU1	7.34E-05	0.015	567	0.459
ENSP00000259808	RIPK1	6.19E-05	0.015	998	0.648
ENSP00000360891	IFIT2	5.78E-05	0.015	927	0.565
ENSP00000337759	DOM3Z	5.78E-05	0.015	621	0.137
ENSP00000315474	GIMAP7	5.57E-05	0.015	832	0.607
ENSP00000368226	GK	5.04E-05	0.015	603	0.190
ENSP00000399298	HLA-DPB1	4.70E-05	0.015	296	0.734

ENSP00000397181	RGS4	4.69E-05	0.015	902	0.468
ENSP00000360730	IL13RA1	4.65E-05	0.015	998	0.530
ENSP00000276449	STAR	4.64E-05	0.015	800	0.354
ENSP00000405108	HLA-DOB	4.53E-05	0.015	587	0.265
ENSP00000264122	CBLB	4.47E-05	0.015	899	0.429
ENSP00000395780	HLA-DOB	4.40E-05	0.015	587	0.265
ENSP00000228740	LTA4H	3.92E-05	0.015	384	0.339
ENSP00000400626	SKIV2L	3.67E-05	0.015	369	0.162
ENSP00000336764	OPRL1	3.53E-05	0.015	899	0.461
ENSP00000357112	AIM2	3.43E-05	0.015	829	0.694
ENSP00000395102	LTB	2.76E-05	0.015	340	0.503
ENSP00000223795	TNFSF8	2.61E-05	0.015	508	0.582
ENSP00000351484	NAALADL1	2.12E-05	0.015	502	0.395
ENSP00000363768	C1QC	1.06E-04	0.016	900	0.554
ENSP00000307786	CYCS	1.06E-04	0.016	905	0.753
ENSP00000263125	PRKCQ	8.62E-05	0.016	964	0.415
ENSP00000327025	RORC	7.49E-05	0.016	705	0.731
ENSP00000364782	NEU1	7.24E-05	0.016	609	0.459
ENSP00000005180	CCL26	6.91E-05	0.016	980	0.431
ENSP00000368699	ISG15	6.85E-05	0.016	932	0.591
ENSP00000305416	S1PR1	6.68E-05	0.016	946	0.601
ENSP00000327336	BGN	6.36E-05	0.016	958	0.552

ENSP00000397705	HLA-F	6.26E-05	0.016	845	0.389
ENSP00000254868	CLEC10A	5.68E-05	0.016	506	0.578
ENSP00000328133	IL20RB	5.17E-05	0.016	810	0.524
ENSP00000340937	COL17A1	4.32E-05	0.016	427	0.137
ENSP00000378786	HLA-DRA	4.26E-05	0.016	866	0.654
ENSP00000379651	LILRA6	4.24E-05	0.016	427	0.566
ENSP00000266003	MLN	3.68E-05	0.016	899	0.489
ENSP00000391879	TRIM26	3.08E-05	0.016	294	0.323
ENSP00000415328	TRIM26	3.07E-05	0.016	294	0.323
ENSP00000291744	FCN2	2.00E-05	0.016	937	0.427
ENSP00000346839	FN1	1.69E-04	0.017	993	0.763
ENSP00000277541	NOTCH1	1.44E-04	0.017	997	0.797
ENSP00000262539	PTPN3	1.02E-04	0.017	927	0.692
ENSP00000343428	GPR18	9.05E-05	0.017	945	0.520
ENSP00000304895	IRS1	8.04E-05	0.017	999	0.685
ENSP00000310127	IRF3	7.87E-05	0.017	986	0.684
ENSP00000298171	TSHR	6.11E-05	0.017	939	0.401
ENSP00000317327	UBASH3A	5.88E-05	0.017	636	0.225
ENSP00000348793	AFF3	5.71E-05	0.017	428	0.435
ENSP00000351314	PSMB10	5.63E-05	0.017	919	0.384
ENSP00000398412	MICB	5.62E-05	0.017	899	0.509
ENSP00000347379	OCLN	5.49E-05	0.017	920	0.493

ENSP00000244061	RNF114	5.14E-05	0.017	401	0.312
ENSP00000280200	CD226	4.72E-05	0.017	333	0.514
ENSP00000383503	ZNRD1	4.37E-05	0.017	388	0.137
ENSP00000228918	LTBR	3.70E-05	0.017	845	0.604
ENSP00000300571	GPRC5B	3.38E-05	0.017	200	0.208
ENSP00000278888	MS4A2	3.26E-05	0.017	540	0.580
ENSP00000307259	P2RY12	2.91E-05	0.017	900	0.501
ENSP00000346892	SERF1A	2.65E-05	0.017	195	0.320
ENSP00000262629	TYROBP	1.80E-04	0.018	933	0.687
ENSP00000276414	GNRH1	1.06E-04	0.018	901	0.518
ENSP00000406878	PSMB8	1.02E-04	0.018	910	0.321
ENSP00000353940	PMEL	6.64E-05	0.018	953	0.415
ENSP00000384886	IFI30	6.37E-05	0.018	922	0.477
ENSP00000264972	ZAP70	5.99E-05	0.018	967	0.545
ENSP00000365817	HLA-E	5.92E-05	0.018	896	0.707
ENSP00000381129	GRK4	5.02E-05	0.018	805	0.181
ENSP00000348912	ADAM33	4.12E-05	0.018	461	0.454
ENSP00000410626	PHRF1	3.98E-05	0.018	517	0.416
ENSP00000338171	SKAP1	3.87E-05	0.018	351	0.302
ENSP00000373167	TIGIT	3.84E-05	0.018	559	0.568
ENSP00000258749	AOAH	3.77E-05	0.018	601	0.648
ENSP00000347314	SPG20	3.67E-05	0.018	430	0.198

ENSP00000265857	GET4	3.53E-05	0.018	318	0.506
ENSP00000251377	LILRA2	3.47E-05	0.018	336	0.458
ENSP00000327647	CRADD	2.86E-05	0.018	507	0.526
ENSP00000411683	TRIM15	2.54E-05	0.018	288	0.353
ENSP00000385470	TNFSF18	2.02E-05	0.018	720	0.443
ENSP00000355865	PARK2	9.40E-05	0.019	998	0.643
ENSP00000236850	APOA1	8.72E-05	0.019	764	0.609
ENSP00000366124	CST3	8.55E-05	0.019	651	0.504
ENSP00000353224	TFRC	8.50E-05	0.019	907	0.498
ENSP00000351777	VCP	8.24E-05	0.019	630	0.243
ENSP00000361850	PLAU	8.14E-05	0.019	922	0.717
ENSP00000382819	DOM3Z	5.80E-05	0.019	621	0.137
ENSP00000303149	GPR27	5.18E-05	0.019	319	0.219
ENSP00000257570	OASL	5.17E-05	0.019	910	0.298
ENSP00000319279	MALT1	4.78E-05	0.019	991	0.642
ENSP00000354159	NLRC4	4.65E-05	0.019	998	0.732
ENSP00000357625	BNIP3	4.22E-05	0.019	890	0.407
ENSP00000371532	VLDLR	3.71E-05	0.019	303	0.343
ENSP00000295463	ALPI	3.69E-05	0.019	621	0.143
ENSP00000364543	SKIV2L	3.68E-05	0.019	369	0.162
ENSP00000305877	NMUR1	3.52E-05	0.019	899	0.493
ENSP00000395598	TRIM10	3.15E-05	0.019	296	0.373

ENSP00000365884	TRIM15	2.54E-05	0.019	288	0.353
ENSP00000269394	ZNF750	2.49E-05	0.019	425	0.463
ENSP00000412897	IFNA4	1.41E-05	0.019	430	0.464
ENSP00000308938	PLG	9.98E-05	0.02	977	0.684
ENSP00000348812	PTPRD	7.53E-05	0.02	767	0.540
ENSP00000379330	NFATC2	7.21E-05	0.02	999	0.543
ENSP00000340292	DLK1	5.03E-05	0.02	424	0.593
ENSP00000338799	IL6ST	5.02E-05	0.02	999	0.659
ENSP00000416599	ZNRD1	4.41E-05	0.02	388	0.137
ENSP00000405636	ZNRD1	4.18E-05	0.02	388	0.137
ENSP00000309968	ADAM17	4.11E-05	0.02	860	0.551
ENSP00000393744	PSMB9	3.64E-05	0.02	714	0.293
ENSP00000363993	PSMB9	3.62E-05	0.02	714	0.293
ENSP00000372718	HLA-DMB	1.62E-05	0.02	232	0.681
ENSP00000413471	HLA-DMB	1.60E-05	0.02	232	0.681
ENSP00000363941	HLA-DPA1	1.51E-05	0.02	173	0.380
ENSP00000264998	TF	1.22E-04	0.021	838	0.700
ENSP00000356671	SERPINC1	1.02E-04	0.021	991	0.640
ENSP00000353731	DPP4	7.56E-05	0.021	896	0.419
ENSP00000258298	TEX35	7.28E-05	0.021	891	0.311
ENSP00000246115	S1PR4	6.97E-05	0.021	971	0.605
ENSP00000399388	TCF19	6.17E-05	0.021	899	0.230

ENSP00000299663	CLEC4E	4.41E-05	0.021	540	0.548
ENSP00000348472	PXK	4.41E-05	0.021	516	0.436
ENSP00000242465	SRGN	4.32E-05	0.021	900	0.393
ENSP00000348888	PIGR	4.16E-05	0.021	843	0.314
ENSP00000293599	AQP5	3.73E-05	0.021	879	0.470
ENSP00000355136	SORBS1	3.60E-05	0.021	424	0.193
ENSP00000376765	PIAS3	3.52E-05	0.021	997	0.388
ENSP00000283243	PLA2R1	3.19E-05	0.021	800	0.127
ENSP00000325663	NFKBIZ	3.15E-05	0.021	764	0.498
ENSP00000410512	HLA-DQB2	2.57E-05	0.021	733	0.294
ENSP00000391938	HLA-DQB2	2.57E-05	0.021	733	0.294
ENSP00000371505	CLEC6A	2.28E-05	0.021	613	0.524
ENSP00000390224	C1orf132	2.05E-05	0.021	245	0.000
ENSP00000243213	IL13RA2	2.03E-05	0.021	903	0.520
ENSP00000221972	CD79A	2.25E-04	0.022	886	0.790
ENSP00000263642	IFIH1	8.33E-05	0.022	792	0.649
ENSP00000256646	NOTCH2	6.88E-05	0.022	994	0.780
ENSP00000268389	IGSF6	6.12E-05	0.022	788	0.674
ENSP00000303208	PCSK9	5.49E-05	0.022	815	0.264
ENSP00000332468	TRAF3	5.23E-05	0.022	999	0.710
ENSP00000302812	SP7	4.97E-05	0.022	898	0.694
ENSP00000348068	SERPINA1	4.55E-05	0.022	909	0.544

ENSP00000226840	AADAT	4.53E-05	0.022	306	0.534
ENSP00000237455	RNF103	4.29E-05	0.022	285	0.173
ENSP00000356530	RNASEL	4.20E-05	0.022	914	0.452
ENSP00000372726	TAP2	3.62E-05	0.022	293	0.177
ENSP00000264613	CP	3.39E-05	0.022	659	0.556
ENSP00000256906	HRH4	3.34E-05	0.022	904	0.613
ENSP00000331827	TNFAIP8L1	3.04E-05	0.022	336	0.314
ENSP00000263413	C6	2.70E-05	0.022	214	0.329
ENSP00000356385	CFHR2	2.56E-05	0.022	188	0.465
ENSP00000368349	POLA1	1.57E-04	0.023	659	0.692
ENSP00000272190	REN	7.84E-05	0.023	906	0.632
ENSP00000258317	NPL	7.17E-05	0.023	462	0.362
ENSP00000261254	CCND2	6.36E-05	0.023	887	0.697
ENSP00000341208	STAT5A	6.19E-05	0.023	999	0.719
ENSP00000221496	AMH	5.70E-05	0.023	427	0.549
ENSP00000340191	FPR2	5.16E-05	0.023	920	0.615
ENSP00000338812	C1QTNF6	5.14E-05	0.023	800	0.429
ENSP00000305133	SOCS5	4.79E-05	0.023	993	0.417
ENSP00000258381	SP110	4.74E-05	0.023	385	0.475
ENSP00000347032	PIP4K2C	4.31E-05	0.023	308	0.261
ENSP00000363571	MUSK	4.11E-05	0.023	470	0.575
ENSP00000364801	HSPA1B	3.99E-05	0.023	340	0.399

ENSP00000382915	HSPA1A	3.99E-05	0.023	340	0.603
ENSP00000375391	HSPA1B	3.99E-05	0.023	340	0.399
ENSP00000375399	HSPA1B	3.99E-05	0.023	340	0.399
ENSP00000403530	HSPA1B	3.99E-05	0.023	340	0.399
ENSP00000365596	GPR183	3.95E-05	0.023	729	0.640
ENSP00000329380	GP1BA	3.91E-05	0.023	390	0.314
ENSP00000372881	HSPA1L	3.84E-05	0.023	337	0.272
ENSP00000408347	HSPA1L	3.84E-05	0.023	337	0.272
ENSP00000331111	ZNRD1	3.68E-05	0.023	404	0.137
ENSP00000262999	UCP1	3.48E-05	0.023	894	0.410
ENSP00000350198	SSTR2	3.48E-05	0.023	900	0.511
ENSP00000265572	OPRK1	3.37E-05	0.023	900	0.467
ENSP00000302517	HLA-DRB3	3.27E-05	0.023	580	0.329
ENSP00000354676	OSTC	3.22E-05	0.023	542	0.448
ENSP00000296503	HMGB2	3.19E-05	0.023	300	0.331
ENSP00000393958	DHX16	3.10E-05	0.023	899	0.148
ENSP00000343000	IL19	2.99E-05	0.023	904	0.554
ENSP00000360966	SLC2A6	2.81E-05	0.023	165	0.133
ENSP00000281092	FER	1.53E-05	0.023	951	0.501
ENSP00000347858	XIAP	5.59E-05	0.024	999	0.678
ENSP00000406797	PSMB8	5.20E-05	0.024	463	0.321
ENSP00000268704	SPG7	4.80E-05	0.024	859	0.647

ENSP00000255427	CHIT1	4.60E-05	0.024	385	0.156
ENSP00000299642	CLEC3A	4.30E-05	0.024	502	0.583
ENSP00000357123	MNDA	3.61E-05	0.024	506	0.512
ENSP00000257254	APLNR	3.31E-05	0.024	907	0.479
ENSP00000329507	CD300C	2.83E-05	0.024	328	0.273
ENSP00000356189	IPCEF1	2.72E-05	0.024	274	0.194
ENSP00000384273	RELA	2.00E-04	0.025	993	0.735
ENSP00000357753	IVL	8.86E-05	0.025	551	0.404
ENSP00000338548	FGF1	8.62E-05	0.025	854	0.787
ENSP00000302269	VAV1	8.51E-05	0.025	983	0.600
ENSP00000351905	TGFBR2	6.29E-05	0.025	997	0.691
ENSP00000408617	HDAC9	6.22E-05	0.025	942	0.643
ENSP00000342070	CTSB	5.75E-05	0.025	886	0.627
ENSP00000286452	KIF5A	4.96E-05	0.025	419	0.181
ENSP00000255040	APCS	4.80E-05	0.025	892	0.591
ENSP00000362057	NOX1	4.26E-05	0.025	851	0.483
ENSP00000408907	HSPA1A	4.07E-05	0.025	340	0.603
ENSP00000360157	FOXD3	4.01E-05	0.025	408	0.610
ENSP00000279441	MMP10	3.90E-05	0.025	888	0.460
ENSP00000293897	SSTR5	3.52E-05	0.025	902	0.488
ENSP00000275015	NFKBIE	3.10E-05	0.025	672	0.501
ENSP00000296980	IL22RA2	3.02E-05	0.025	997	0.383

ENSP00000287907	HTR5A	2.97E-05	0.025	904	0.521
ENSP00000265440	TFEC	2.14E-05	0.025	601	0.442
ENSP00000265171	EGF	1.41E-04	0.026	993	0.793
ENSP00000363976	HLA-DMA	1.04E-04	0.026	961	0.564
ENSP00000358997	IRAK1	7.99E-05	0.026	999	0.736
ENSP00000406359	HSPA1A	6.78E-05	0.026	918	0.603
ENSP00000359490	GBP4	5.97E-05	0.026	899	0.452
ENSP00000335620	GSTA1	5.75E-05	0.026	750	0.685
ENSP00000257749	BACH2	5.19E-05	0.026	460	0.288
ENSP00000345344	CTSL1	4.87E-05	0.026	996	0.544
ENSP00000260302	MMP13	4.57E-05	0.026	928	0.689
ENSP00000356395	CFHR3	4.49E-05	0.026	424	0.355
ENSP00000271411	POU2F1	4.36E-05	0.026	657	0.337
ENSP00000412027	PSMB9	3.68E-05	0.026	657	0.293
ENSP00000403721	CYP21A2	3.57E-05	0.026	409	0.199
ENSP00000341828	CHIA	3.55E-05	0.026	395	0.368
ENSP00000299440	RAG1	3.42E-05	0.026	543	0.576
ENSP00000361467	DLG5	3.28E-05	0.026	669	0.441
ENSP00000267377	SSTR1	3.23E-05	0.026	901	0.493
ENSP00000295980	IL17RE	3.15E-05	0.026	514	0.468
ENSP00000217407	LBP	3.03E-05	0.026	922	0.562
ENSP00000222305	USF2	2.66E-05	0.026	296	0.369

ENSP00000302924	IL17D	2.12E-05	0.026	527	0.382
ENSP00000352514	RUNX2	1.82E-04	0.027	927	0.773
ENSP00000351273	CASP8	9.66E-05	0.027	999	0.821
ENSP00000407195	TAPBP	8.60E-05	0.027	896	0.280
ENSP00000362463	GLO1	6.06E-05	0.027	420	0.314
ENSP00000341268	TRADD	5.99E-05	0.027	999	0.664
ENSP00000363081	DKK1	5.36E-05	0.027	874	0.786
ENSP00000307235	EIF2AK3	4.51E-05	0.027	643	0.711
ENSP00000308208	MMP14	4.40E-05	0.027	894	0.679
ENSP00000300184	MS4A7	4.36E-05	0.027	707	0.746
ENSP00000204615	THPO	3.59E-05	0.027	901	0.652
ENSP00000394942	CYP21A2	3.38E-05	0.027	340	0.199
ENSP00000390497	TRIM10	3.16E-05	0.027	296	0.373
ENSP00000278379	SLC1A2	3.10E-05	0.027	877	0.205
ENSP00000236826	MMP8	3.05E-05	0.027	719	0.559
ENSP00000362181	NCR2	2.92E-05	0.027	424	0.576
ENSP00000316228	CLEC4M	2.72E-05	0.027	872	0.494
ENSP00000233156	TFPI	2.22E-05	0.027	927	0.640
ENSP00000364512	G6PC2	2.07E-05	0.027	569	0.143
ENSP00000279804	CTF1	1.97E-05	0.027	929	0.522
ENSP00000274335	PIK3R1	9.39E-05	0.028	999	0.624
ENSP00000253408	GFAP	7.66E-05	0.028	902	0.705

ENSP00000323929	A2M	5.45E-05	0.028	929	0.635
ENSP00000370880	MLANA	5.44E-05	0.028	954	0.386
ENSP00000312027	CD7	5.33E-05	0.028	788	0.574
ENSP00000231751	LTF	5.20E-05	0.028	901	0.664
ENSP00000008938	PGLYRP1	5.14E-05	0.028	505	0.569
ENSP00000348831	NUDT10	4.49E-05	0.028	429	0.331
ENSP00000307218	NAT1	4.35E-05	0.028	514	0.441
ENSP00000265394	CPVL	4.29E-05	0.028	274	0.237
ENSP00000221403	DHDH	4.19E-05	0.028	562	0.261
ENSP00000360216	DNTT	4.08E-05	0.028	839	0.744
ENSP00000341821	FPR3	3.81E-05	0.028	918	0.573
ENSP00000396813	PSMB9	3.74E-05	0.028	657	0.293
ENSP00000351706	GK2	3.72E-05	0.028	247	0.354
ENSP00000408860	CYP21A2	3.38E-05	0.028	340	0.199
ENSP00000375066	HCAR2	3.33E-05	0.028	907	0.591
ENSP00000297469	GPER	3.23E-05	0.028	900	0.559
ENSP00000305464	APLN	3.05E-05	0.028	938	0.511
ENSP00000389155	ABHD16A	3.03E-05	0.028	406	0.181
ENSP00000377978	CCL4L2	2.86E-05	0.028	482	0.534
ENSP00000342520	IL17REL	2.26E-05	0.028	310	0.482
ENSP00000317980	IGSF9B	1.71E-05	0.028	201	0.343
ENSP00000263733	FAM20B	1.61E-05	0.028	201	0.221

ENSP00000397139	HLA-DPA1	1.51E-05	0.028	173	0.380
ENSP00000215781	OSM	6.14E-05	0.029	981	0.711
ENSP00000356430	RGS18	5.60E-05	0.029	955	0.654
ENSP00000366525	FTL	5.60E-05	0.029	906	0.520
ENSP00000085219	CD22	5.50E-05	0.029	712	0.624
ENSP00000265371	NRP1	5.50E-05	0.029	901	0.654
ENSP00000368880	FOXO1	5.29E-05	0.029	988	0.646
ENSP00000348089	ERCC6	5.21E-05	0.029	528	0.165
ENSP00000245222	SPHK2	4.89E-05	0.029	901	0.161
ENSP00000381793	GRB10	4.80E-05	0.029	800	0.706
ENSP00000363512	ALOX5	4.69E-05	0.029	877	0.568
ENSP00000263056	MAP3K8	3.91E-05	0.029	707	0.586
ENSP00000312455	CFLAR	3.70E-05	0.029	872	0.573
ENSP00000372599	TAP2	3.65E-05	0.029	293	0.177
ENSP00000357906	TNFAIP8L2	3.52E-05	0.029	581	0.644
ENSP00000333779	EVI2B	3.45E-05	0.029	498	0.512
ENSP00000261187	SLC16A7	2.59E-05	0.029	841	0.115
ENSP00000404450	LTB	1.93E-05	0.029	340	0.503
ENSP00000286301	CSF1R	1.04E-04	0.03	907	0.678
ENSP00000282561	GJA1	7.49E-05	0.03	905	0.577
ENSP00000289473	NCF1	6.06E-05	0.03	621	0.498
ENSP00000313875	CD46	5.75E-05	0.03	868	0.527

ENSP00000211122	GSTA3	5.48E-05	0.03	675	0.697
ENSP00000414905	HLA-F	5.12E-05	0.03	551	0.389
ENSP00000246553	FFAR1	4.02E-05	0.03	899	0.529
ENSP00000359643	LPAR3	3.94E-05	0.03	899	0.502
ENSP00000355797	TCP10L2	3.68E-05	0.03	238	0.325
ENSP00000314897	ANGPT2	3.38E-05	0.03	867	0.716
ENSP00000227163	SPI1	3.38E-05	0.03	859	0.521
ENSP00000356251	GPR37L1	3.32E-05	0.03	201	0.281
ENSP00000263119	CABIN1	3.21E-05	0.03	461	0.410
ENSP00000327513	CSF1	3.09E-05	0.03	769	0.644
ENSP00000339377	NPY5R	2.89E-05	0.03	899	0.486
ENSP00000248244	TICAM1	2.77E-05	0.03	978	0.642
ENSP00000365431	TCF19	2.68E-05	0.03	523	0.230
ENSP00000295755	RETNLB	2.36E-05	0.03	411	0.529
ENSP00000416448	HLA-DOA	2.26E-05	0.03	806	0.249
ENSP00000378856	LGALS9	2.21E-05	0.03	517	0.422
ENSP00000360141	GNAS	9.05E-05	0.031	462	0.345
ENSP00000282091	PTH	8.20E-05	0.031	926	0.585
ENSP00000268035	IGF1R	7.09E-05	0.031	825	0.712
ENSP00000356708	OLIG3	6.09E-05	0.031	542	0.435
ENSP00000420168	GSTA2	5.52E-05	0.031	750	0.690
ENSP00000386161	WWOX	5.04E-05	0.031	864	0.410

ENSP00000285850	SLC7A7	4.29E-05	0.031	653	0.558
ENSP00000383953	RGS6	2.86E-05	0.031	899	0.487
ENSP00000297904	FIGF	2.51E-05	0.031	908	0.660
ENSP00000227348	CRTAM	2.33E-05	0.031	526	0.432
ENSP00000299694	BEAN1	1.54E-05	0.031	260	0.191
ENSP00000261405	VWF	9.53E-05	0.032	752	0.689
ENSP00000343957	IFNAR2	6.47E-05	0.032	976	0.690
ENSP00000347988	NDUFA5	6.43E-05	0.032	609	0.191
ENSP00000361965	ADA	5.41E-05	0.032	526	0.377
ENSP00000298902	IFI27	4.29E-05	0.032	922	0.267
ENSP00000289707	SLAMF8	4.29E-05	0.032	649	0.660
ENSP00000272644	GPR17	3.84E-05	0.032	901	0.509
ENSP00000302324	RNASE3	3.75E-05	0.032	540	0.488
ENSP00000379500	UEVLD	3.42E-05	0.032	429	0.206
ENSP00000262630	ZBTB32	3.16E-05	0.032	493	0.426
ENSP00000294984	IL24	3.14E-05	0.032	899	0.390
ENSP00000360215	ZBP1	3.06E-05	0.032	425	0.592
ENSP00000370935	CSF2RA	2.82E-05	0.032	944	0.551
ENSP00000353238	SPN	2.79E-05	0.032	745	0.427
ENSP00000278070	PPRC1	2.31E-05	0.032	251	0.126
ENSP00000386341	TICAM2	2.05E-05	0.032	999	0.609
ENSP00000270202	AKT1	2.45E-04	0.033	994	0.659

ENSP00000304736	ELOVL6	1.20E-04	0.033	794	0.308
ENSP00000264498	FGF2	9.10E-05	0.033	926	0.765
ENSP00000312999	GNAI2	8.44E-05	0.033	967	0.448
ENSP00000302564	BCL2L1	7.45E-05	0.033	930	0.725
ENSP00000359998	GSTA4	5.63E-05	0.033	730	0.696
ENSP00000300289	PDIA3	5.03E-05	0.033	965	0.149
ENSP00000247470	PYCARD	4.97E-05	0.033	987	0.724
ENSP00000373614	SELPLG	4.90E-05	0.033	563	0.591
ENSP00000262741	PIK3R3	4.81E-05	0.033	899	0.434
ENSP00000379908	ICA1	4.31E-05	0.033	327	0.325
ENSP00000357066	ARG1	4.29E-05	0.033	881	0.476
ENSP00000405306	NCR3	3.96E-05	0.033	361	0.471
ENSP00000389071	NCR3	3.96E-05	0.033	361	0.471
ENSP00000297450	ANGPT1	3.52E-05	0.033	877	0.714
ENSP00000356791	DPT	3.22E-05	0.033	800	0.250
ENSP00000363643	P2RY4	3.08E-05	0.033	899	0.503
ENSP00000398224	TRIM15	2.91E-05	0.033	357	0.353
ENSP00000244174	IL9R	2.81E-05	0.033	860	0.529
ENSP00000310814	STARD6	2.50E-05	0.033	168	0.169
ENSP00000364858	CARD16	2.38E-05	0.033	958	0.471
ENSP00000358031	C1orf138	1.48E-05	0.033	899	0.485
ENSP00000309845	HRAS	2.74E-04	0.034	948	0.778

ENSP00000353483	MAPK8	1.03E-04	0.034	925	0.847
ENSP00000261267	LYZ	9.25E-05	0.034	611	0.623
ENSP00000271324	CD53	7.08E-05	0.034	747	0.539
ENSP00000359174	SLC35A3	5.66E-05	0.034	899	0.069
ENSP00000264716	FOSL2	4.29E-05	0.034	397	0.397
ENSP00000310405	XCR1	3.93E-05	0.034	899	0.591
ENSP00000238607	PGF	3.64E-05	0.034	884	0.724
ENSP00000234961	OPRD1	3.59E-05	0.034	931	0.434
ENSP00000357113	IFI16	3.35E-05	0.034	902	0.431
ENSP00000319308	RGS5	3.23E-05	0.034	800	0.288
ENSP00000317337	CD300LB	2.94E-05	0.034	368	0.242
ENSP00000349003	PTGER3	2.92E-05	0.034	902	0.520
ENSP00000329684	GALR2	2.88E-05	0.034	899	0.518
ENSP00000364217	AGER	2.85E-05	0.034	889	0.605
ENSP00000253699	ZFYVE20	2.80E-05	0.034	528	0.114
ENSP00000386588	RGPD3	2.51E-05	0.034	540	0.307
ENSP00000358188	NBPF15	1.20E-05	0.034	374	0.262
ENSP00000256078	KRAS	1.03E-04	0.035	825	0.721
ENSP00000218388	TIMP1	9.14E-05	0.035	934	0.725
ENSP00000222390	HGF	8.51E-05	0.035	941	0.788
ENSP00000357731	LOR	8.06E-05	0.035	469	0.365
ENSP00000353393	F8	5.78E-05	0.035	913	0.652

ENSP00000264664	FGF10	5.37E-05	0.035	867	0.792
ENSP00000410443	HLA-DRA	5.22E-05	0.035	822	0.654
ENSP00000355988	IRF6	4.99E-05	0.035	955	0.480
ENSP00000404619	PRRC2A	4.99E-05	0.035	362	0.208
ENSP00000222139	EPOR	4.71E-05	0.035	999	0.619
ENSP00000265643	GAL	4.60E-05	0.035	904	0.455
ENSP00000414817	HLA-DMB	4.25E-05	0.035	836	0.681
ENSP00000384665	LPAR2	4.07E-05	0.035	926	0.525
ENSP00000364805	HSPA1L	3.89E-05	0.035	337	0.272
ENSP00000366061	NMS	3.86E-05	0.035	901	0.461
ENSP00000379566	CCHCR1	3.14E-05	0.035	462	0.271
ENSP00000259075	TANK	3.00E-05	0.035	418	0.616
ENSP00000380150	CARD11	2.89E-05	0.035	913	0.545
ENSP00000395497	GTF2H4	2.75E-05	0.035	408	0.120
ENSP00000256733	SAA2	2.18E-05	0.035	788	0.528
ENSP00000320168	NUCB2	2.04E-05	0.035	844	0.114
ENSP00000272324	REG3G	1.59E-05	0.035	675	0.238
ENSP00000358016	SCML4	1.07E-05	0.035	179	0.407
ENSP00000247461	CANX	8.95E-05	0.036	939	0.290
ENSP00000360876	IFIT3	5.06E-05	0.036	912	0.507
ENSP00000363804	KLF4	4.91E-05	0.036	434	0.683
ENSP00000308782	GP6	3.97E-05	0.036	630	0.422

ENSP00000367086	ACOT7	3.36E-05	0.036	899	0.134
ENSP00000339952	KSR2	3.31E-05	0.036	336	0.086
ENSP00000403628	CCHCR1	3.05E-05	0.036	460	0.271
ENSP00000313661	HTR1D	2.90E-05	0.036	899	0.471
ENSP00000339637	BRAT1	2.48E-05	0.036	168	0.328
ENSP00000412297	PPP1R11	2.37E-05	0.036	430	0.283
ENSP00000365692	PIM2	1.70E-05	0.036	828	0.315
ENSP00000368914	PSTPIP1	5.05E-05	0.037	995	0.401
ENSP00000390120	LILRA6	4.09E-05	0.037	410	0.566
ENSP00000342112	LMO7	3.41E-05	0.037	230	0.129
ENSP00000332591	NPY2R	3.02E-05	0.037	903	0.511
ENSP00000301908	PNOC	3.00E-05	0.037	905	0.495
ENSP00000255008	SSTR4	2.91E-05	0.037	900	0.498
ENSP00000309103	BAD	2.87E-05	0.037	946	0.521
ENSP00000229922	CAP2	2.56E-05	0.037	378	0.107
ENSP00000291232	TNFRSF13C	2.53E-05	0.037	517	0.571
ENSP00000410390	HLA-DOB	2.26E-05	0.037	810	0.265
ENSP00000393667	FAM126B	1.92E-05	0.037	238	0.219
ENSP00000321203	TNIP2	1.64E-05	0.037	992	0.564
ENSP00000354558	MTOR	9.39E-05	0.038	998	0.763
ENSP00000312286	PLA2G1B	6.96E-05	0.038	921	0.669
ENSP00000230990	HBEGF	5.83E-05	0.038	947	0.726

ENSP00000268058	PML	5.81E-05	0.038	906	0.576
ENSP00000357697	S100A2	5.04E-05	0.038	899	0.114
ENSP00000294507	LAPTM5	4.57E-05	0.038	650	0.485
ENSP00000295981	IL17RC	3.58E-05	0.038	950	0.470
ENSP00000265447	ANXA11	3.28E-05	0.038	361	0.257
ENSP00000386069	ADRA2C	2.94E-05	0.038	899	0.470
ENSP00000368538	TNFRSF4	2.78E-05	0.038	901	0.498
ENSP00000308774	BMX	2.55E-05	0.038	959	0.373
ENSP00000338260	APOL4	1.90E-05	0.038	263	0.137
ENSP00000366234	IL31	1.88E-05	0.038	424	0.521
ENSP00000250377	KIAA0391	1.50E-05	0.038	195	0.109
ENSP00000228280	KITLG	9.60E-05	0.039	958	0.810
ENSP00000226359	AFP	6.65E-05	0.039	624	0.349
ENSP00000239938	EGR1	6.39E-05	0.039	904	0.709
ENSP00000404524	HSPA1A	6.22E-05	0.039	911	0.603
ENSP00000393087	HSPA1B	5.77E-05	0.039	430	0.399
ENSP00000387477	PRRC2A	5.03E-05	0.039	362	0.208
ENSP00000351997	MAP2K6	5.01E-05	0.039	933	0.662
ENSP00000244709	TREM1	3.48E-05	0.039	493	0.601
ENSP00000359540	CREG1	3.20E-05	0.039	463	0.218
ENSP00000264501	KIAA1109	3.07E-05	0.039	420	0.375
ENSP00000302227	CCBL1	3.01E-05	0.039	290	0.470

ENSP00000299727	GALR1	2.92E-05	0.039	900	0.495
ENSP00000307766	HTR1E	2.90E-05	0.039	900	0.512
ENSP00000257622	POM121	2.58E-05	0.039	196	0.166
ENSP00000262305	RAB11FIP3	2.39E-05	0.039	899	0.228
ENSP00000391349	DOM3Z	5.79E-05	0.04	621	0.137
ENSP00000363435	ITPR3	5.56E-05	0.04	366	0.345
ENSP00000303532	DEFB4A	5.39E-05	0.04	979	0.631
ENSP00000028008	RNASET2	5.16E-05	0.04	462	0.147
ENSP00000381607	GSTP1	4.72E-05	0.04	664	0.562
ENSP00000415481	PROM1	4.36E-05	0.04	865	0.697
ENSP00000373477	GPX3	4.17E-05	0.04	882	0.369
ENSP00000371341	TNK2	3.67E-05	0.04	645	0.532
ENSP00000289429	CD1A	3.65E-05	0.04	385	0.430
ENSP00000358730	ADORA3	3.65E-05	0.04	907	0.573
ENSP00000351901	CARD8	3.08E-05	0.04	993	0.788
ENSP00000391181	CCHCR1	2.98E-05	0.04	462	0.271
ENSP00000370126	SERF1B	2.98E-05	0.04	196	0.193
ENSP00000358099	RGS10	2.90E-05	0.04	900	0.515
ENSP00000328364	MAFA	1.79E-05	0.04	338	0.243
ENSP00000359291	ZNF185	1.66E-05	0.04	825	0.435
ENSP00000369558	IFNA6	1.49E-05	0.04	910	0.474
ENSP00000365663	NPPA	6.64E-05	0.041	919	0.538

ENSP00000386187	IFITM1	4.51E-05	0.041	916	0.343
ENSP00000365775	MTHFR	3.93E-05	0.041	867	0.459
ENSP00000278927	ESAM	3.92E-05	0.041	854	0.563
ENSP00000216629	BDKRB1	3.60E-05	0.041	901	0.495
ENSP00000342560	HRH3	3.18E-05	0.041	899	0.512
ENSP00000356658	GPR52	3.10E-05	0.041	176	0.326
ENSP00000260061	LRRC32	2.95E-05	0.041	380	0.222
ENSP00000224600	RBP3	2.79E-05	0.041	462	0.298
ENSP00000351650	NUDT3	2.20E-05	0.041	395	0.000
ENSP00000302961	HSPA4	1.04E-04	0.042	962	0.635
ENSP00000318902	FOXP1	4.62E-05	0.042	326	0.437
ENSP00000229319	LDHB	4.25E-05	0.042	425	0.178
ENSP00000361202	IRS4	3.83E-05	0.042	437	0.351
ENSP00000375893	GPR55	3.19E-05	0.042	899	0.516
ENSP00000350348	GRM7	3.11E-05	0.042	336	0.104
ENSP00000359512	GBP3	2.45E-05	0.042	556	0.447
ENSP00000388794	CD177	1.97E-05	0.042	500	0.257
ENSP00000225371	EPX	1.73E-05	0.042	514	0.529
ENSP00000318480	YME1L1	6.93E-05	0.043	659	0.513
ENSP00000327048	MAF	5.15E-05	0.043	829	0.508
ENSP00000357153	CD1D	4.94E-05	0.043	846	0.635
ENSP00000359245	ABCA4	4.54E-05	0.043	430	0.082

ENSP00000360626	KCNG1	4.27E-05	0.043	176	0.174
ENSP00000329991	IFNL1	3.72E-05	0.043	425	0.691
ENSP00000329468	ADAP2	3.57E-05	0.043	517	0.615
ENSP00000263734	EPAS1	3.25E-05	0.043	336	0.483
ENSP00000342513	IFI6	3.06E-05	0.043	918	0.284
ENSP00000332504	CCR10	2.86E-05	0.043	918	0.799
ENSP00000384432	ICOSLG	2.63E-05	0.043	563	0.472
ENSP00000394494	IFNA13	2.54E-05	0.043	374	0.512
ENSP00000237380	MED28	2.43E-05	0.043	427	0.527
ENSP00000371872	SLC35G5	1.46E-05	0.043	409	0.207
ENSP00000292778	YDJC	1.22E-05	0.043	340	0.175
ENSP00000342295	ANKRD55	1.04E-05	0.043	263	0.343
ENSP00000350941	SRC	1.99E-04	0.044	999	0.772
ENSP00000381237	HM13	4.83E-05	0.044	822	0.064
ENSP00000252818	JUND	4.32E-05	0.044	984	0.593
ENSP00000351755	LPAR1	4.13E-05	0.044	902	0.495
ENSP00000370912	TEC	3.97E-05	0.044	988	0.629
ENSP00000415786	SERPINE2	3.79E-05	0.044	437	0.413
ENSP00000268763	KSR1	3.57E-05	0.044	336	0.346
ENSP00000362613	CDX4	3.42E-05	0.044	252	0.371
ENSP00000310117	PPP1R14B	3.28E-05	0.044	238	0.172
ENSP00000401980	MAVS	3.21E-05	0.044	729	0.659

ENSP00000339186	GRAP2	2.96E-05	0.044	919	0.421
ENSP00000224181	C8G	2.76E-05	0.044	297	0.288
ENSP00000300659	NFATC3	2.66E-05	0.044	969	0.329
ENSP00000262290	LPO	2.45E-05	0.044	380	0.495
ENSP00000269571	ERBB2	1.15E-04	0.045	995	0.786
ENSP00000307713	BDKRB2	3.80E-05	0.045	901	0.493
ENSP00000280155	ADRA2A	3.25E-05	0.045	905	0.445
ENSP00000205143	DLL3	1.85E-05	0.045	913	0.666
ENSP00000295897	ALB	3.48E-04	0.046	968	0.774
ENSP00000338983	MUC1	6.56E-05	0.046	866	0.497
ENSP00000314709	ZHX2	4.81E-05	0.046	916	0.762
ENSP00000271651	CTSK	4.58E-05	0.046	899	0.525
ENSP00000370408	CDX2	4.23E-05	0.046	858	0.660
ENSP00000264192	CYTIP	4.07E-05	0.046	574	0.485
ENSP00000327191	CCT6B	4.02E-05	0.046	243	0.197
ENSP00000233202	SLC11A1	3.98E-05	0.046	422	0.546
ENSP00000408453	HLA-DMB	3.94E-05	0.046	440	0.681
ENSP00000199280	AQP2	3.65E-05	0.046	421	0.291
ENSP00000263798	TYRO3	3.57E-05	0.046	817	0.653
ENSP00000233954	IL1RL1	3.32E-05	0.046	330	0.569
ENSP00000354652	NPY1R	3.25E-05	0.046	904	0.519
ENSP00000263437	NLRP2	2.52E-05	0.046	732	0.796

ENSP00000340019	HSPD1	6.61E-05	0.047	978	0.445
ENSP00000222330	GSK3A	4.57E-05	0.047	411	0.488
ENSP00000347948	TNFRSF14	4.41E-05	0.047	464	0.377
ENSP00000369299	TRIM22	4.26E-05	0.047	577	0.483
ENSP00000369858	ALOX5AP	3.85E-05	0.047	576	0.537
ENSP00000363596	CNR2	3.34E-05	0.047	274	0.216
ENSP00000276914	PLIN2	3.04E-05	0.047	861	0.311
ENSP00000318142	FUT7	2.81E-05	0.047	469	0.348
ENSP00000365233	TPP2	2.81E-05	0.047	718	0.445
ENSP00000347427	MINK1	2.78E-05	0.047	424	0.223
ENSP00000357122	PYHIN1	2.78E-05	0.047	462	0.331
ENSP00000255476	RFXAP	2.64E-05	0.047	306	0.229
ENSP00000369531	UBQLNL	2.62E-05	0.047	318	0.245
ENSP00000344844	HAVCR1	2.09E-05	0.047	429	0.347
ENSP00000360560	TCTE1	2.05E-05	0.047	297	0.145
ENSP00000242152	NPY	6.11E-05	0.048	917	0.496
ENSP00000220003	CSK	4.59E-05	0.048	950	0.524
ENSP00000370115	SERPINB1	4.29E-05	0.048	522	0.656
ENSP00000344218	C1orf162	3.21E-05	0.048	611	0.679
ENSP00000322924	HTR1F	2.89E-05	0.048	900	0.493
ENSP00000357020	LY9	2.89E-05	0.048	824	0.498
ENSP00000356595	ABL2	2.74E-05	0.048	253	0.188

ENSP00000371432	PRLR	2.68E-05	0.048	999	0.515
ENSP00000370003	BNIP3L	2.64E-05	0.048	313	0.232
ENSP00000380450	NOXO1	2.53E-05	0.048	243	0.048
ENSP00000295269	SLC9A4	2.18E-05	0.048	308	0.355
ENSP00000242365	KIAA1549	1.50E-05	0.048	252	0.066
ENSP00000346206	TAP1	5.51E-05	0.049	937	0.347
ENSP00000238682	TGFB3	5.37E-05	0.049	961	0.850
ENSP00000266025	TMEM115	4.94E-05	0.049	357	0.174
ENSP00000365465	CDSN	4.91E-05	0.049	554	0.429
ENSP00000315130	CLU	4.04E-05	0.049	902	0.556
ENSP00000370088	AMELX	4.04E-05	0.049	318	0.239
ENSP00000341170	PTN	4.01E-05	0.049	871	0.538
ENSP00000410674	NOTCH4	3.65E-05	0.049	851	0.810
ENSP00000281806	MCHR2	3.51E-05	0.049	899	0.507
ENSP00000261826	P2RX7	3.39E-05	0.049	883	0.493
ENSP00000328472	S1PR5	3.27E-05	0.049	910	0.504
ENSP00000387281	ADRA2B	2.89E-05	0.049	899	0.483
ENSP00000330633	CNTN2	2.75E-05	0.049	621	0.413
ENSP00000261729	RASAL1	2.66E-05	0.049	162	0.063
ENSP00000273183	STAC	2.63E-05	0.049	317	0.389
ENSP00000358902	SAMD10	2.20E-05	0.049	226	0.430
ENSP00000309524	CSHL1	1.18E-05	0.049	800	0.085

ENSP00000308472	EIF4EBP3	1.16E-05	0.049	324	0.292
ENSP00000324806	GSK3B	8.38E-05	0.05	-	-
ENSP00000343027	GNAI1	6.77E-05	0.05	-	-
ENSP00000228928	OAS3	4.16E-05	0.05	-	-
ENSP00000244241	IL17C	3.82E-05	0.05	-	-
ENSP00000251595	HBA2	3.80E-05	0.05	-	-
ENSP00000274793	PLA2G7	3.61E-05	0.05	-	-
ENSP00000223190	NRF1	3.59E-05	0.05	-	-
ENSP00000274695	CDKAL1	3.56E-05	0.05	-	-
ENSP00000353679	MME	3.34E-05	0.05	-	-
ENSP00000357255	BGLAP	3.29E-05	0.05	-	-
ENSP00000367930	OXER1	2.89E-05	0.05	-	-
ENSP00000386229	RGS14	2.88E-05	0.05	-	-
ENSP00000296694	SCGB3A2	2.23E-05	0.05	-	-
ENSP00000326110	C5orf30	1.86E-05	0.05	-	-
ENSP00000362711	TXLNA	1.10E-05	0.05	-	-
ENSP00000357778	LCE3E	5.61E-05	0.051	-	-
ENSP00000388386	CDSN	5.02E-05	0.051	-	-
ENSP00000233057	EIF2AK2	4.96E-05	0.051	-	-
ENSP00000305941	USH2A	3.89E-05	0.051	-	-
ENSP00000375022	IGHV3-30	3.57E-05	0.051	-	-
ENSP00000158762	ACAP1	3.56E-05	0.051	-	-

ENSP00000302846	PTGER4	3.04E-05	0.051	-	-
ENSP00000261464	TRAF5	2.96E-05	0.051	-	-
ENSP00000297313	RGS20	2.87E-05	0.051	-	-
ENSP00000267953	BCL2A1	2.85E-05	0.051	-	-
ENSP00000394290	GNL1	2.63E-05	0.051	-	-
ENSP00000392726	POM121L2	2.21E-05	0.051	-	-
ENSP00000272519	RALB	2.12E-05	0.051	-	-
ENSP00000420298	CD200	2.09E-05	0.051	-	-
ENSP00000274276	OSMR	1.73E-05	0.051	-	-
ENSP00000244869	EREG	1.70E-05	0.051	-	-
ENSP00000357150	CD1B	1.60E-05	0.051	-	-
ENSP00000270452	LILRB4	1.11E-05	0.051	-	-
ENSP00000234590	ENO1	5.94E-05	0.052	-	-
ENSP00000354769	C1orf68	5.43E-05	0.052	-	-
ENSP00000251372	LILRA1	1.34E-05	0.052	-	-
ENSP00000307954	FAM174A	1.08E-05	0.052	-	-
ENSP00000305071	RFXANK	3.68E-05	0.053	-	-
ENSP00000242208	INHBA	3.09E-05	0.053	-	-
ENSP00000379282	ABHD16A	3.06E-05	0.053	-	-
ENSP00000340698	GIPC1	2.96E-05	0.053	-	-
ENSP00000357615	FRK	2.87E-05	0.053	-	-
ENSP00000325708	CD37	2.83E-05	0.053	-	-

ENSP00000246949	DNASE1	2.57E-05	0.053	-	-
ENSP00000350256	CCR9	2.49E-05	0.053	-	-
ENSP00000277632	FAM188A	1.79E-05	0.053	-	-
ENSP00000357789	FLG	6.90E-05	0.054	-	-
ENSP00000353198	PYY	3.16E-05	0.054	-	-
ENSP00000358963	HTR1B	3.06E-05	0.054	-	-
ENSP00000285871	CCDC146	2.25E-05	0.054	-	-
ENSP00000331831	GAS6	2.01E-05	0.054	-	-
ENSP00000351430	GYPE	1.81E-05	0.054	-	-
ENSP00000356700	C1orf105	1.67E-05	0.054	-	-
ENSP00000347997	UBQLN3	1.53E-05	0.054	-	-
ENSP00000320866	CALR	1.31E-04	0.055	-	-
ENSP00000303830	INSR	7.81E-05	0.055	-	-
ENSP00000323795	TEX35	7.29E-05	0.055	-	-
ENSP00000309757	LPL	6.55E-05	0.055	-	-
ENSP00000358404	TCF7L2	4.61E-05	0.055	-	-
ENSP00000362082	CCND3	4.33E-05	0.055	-	-
ENSP00000319248	ZEB1	3.11E-05	0.055	-	-
ENSP00000356213	VIP	3.08E-05	0.055	-	-
ENSP00000243346	NMI	2.77E-05	0.055	-	-
ENSP00000409605	TRIM15	2.71E-05	0.055	-	-
ENSP00000302046	RNASE6	1.73E-05	0.055	-	-

ENSP00000361281	BEST4	1.40E-05	0.055	-	-
ENSP00000309142	PDE12	1.14E-05	0.055	-	-
ENSP00000236671	CTSD	5.62E-05	0.056	-	-
ENSP00000265077	VCAN	4.82E-05	0.056	-	-
ENSP00000231188	GRM6	3.20E-05	0.056	-	-
ENSP00000262065	MMD	3.02E-05	0.056	-	-
ENSP00000286574	WIF1	2.90E-05	0.056	-	-
ENSP00000199764	CEACAM6	2.82E-05	0.056	-	-
ENSP00000192788	UHRF1BP1	2.17E-05	0.056	-	-
ENSP00000295640	RNPEP	2.09E-05	0.056	-	-
ENSP00000374902	TRBV6-9	1.69E-05	0.056	-	-
ENSP00000269445	DYM	1.44E-05	0.056	-	-
ENSP00000306245	FOS	2.16E-04	0.057	-	-
ENSP00000374990	IGHG1	7.01E-05	0.057	-	-
ENSP00000232014	BCL6	6.64E-05	0.057	-	-
ENSP00000357459	ADAR	3.63E-05	0.057	-	-
ENSP00000254436	TRIM21	3.46E-05	0.057	-	-
ENSP00000222307	KXD1	3.42E-05	0.057	-	-
ENSP00000264834	KLF1	3.24E-05	0.057	-	-
ENSP00000326259	OR52H1	3.01E-05	0.057	-	-
ENSP00000362850	DSN1	2.70E-05	0.057	-	-
ENSP00000296130	CLEC3B	2.25E-05	0.057	-	-

ENSP00000362524	ANGPTL2	1.74E-05	0.057	-	-
ENSP00000316675	PVRIG	1.63E-05	0.057	-	-
ENSP00000369553	IFNA8	1.21E-05	0.057	-	-
ENSP00000215832	MAPK1	1.35E-04	0.058	-	-
ENSP00000342952	ADCY2	8.35E-05	0.058	-	-
ENSP00000324740	YES1	6.49E-05	0.058	-	-
ENSP00000365012	HCK	4.69E-05	0.058	-	-
ENSP00000250378	CMA1	3.83E-05	0.058	-	-
ENSP00000342082	SLPI	2.98E-05	0.058	-	-
ENSP00000330070	NPW	2.89E-05	0.058	-	-
ENSP00000320563	WDFY4	2.19E-05	0.058	-	-
ENSP00000370074	SERPINB9	1.37E-05	0.058	-	-
ENSP00000359301	MAGEA3	4.91E-05	0.059	-	-
ENSP00000284486	FAM167A	3.41E-05	0.059	-	-
ENSP00000268605	NOL3	3.20E-05	0.059	-	-
ENSP00000331103	IP6K2	2.72E-05	0.059	-	-
ENSP00000365806	GNL1	2.63E-05	0.059	-	-
ENSP00000283285	CD96	2.62E-05	0.059	-	-
ENSP00000274629	KCNMB1	2.46E-05	0.059	-	-
ENSP00000169298	ST6GAL1	2.36E-05	0.059	-	-
ENSP00000219919	AQP9	2.32E-05	0.059	-	-
ENSP00000400104	RXRB	2.08E-05	0.059	-	-

ENSP00000249066	APOL2	1.93E-05	0.059	-	-
ENSP00000321997	LARP1B	1.53E-05	0.059	-	-
ENSP00000369566	IFNA10	1.27E-05	0.059	-	-
ENSP00000355583	MLK4	1.07E-05	0.059	-	-
ENSP00000358622	IKBKG	8.66E-05	0.06	-	-
ENSP00000355370	CNTF	4.63E-05	0.06	-	-
ENSP00000373169	DRD3	3.73E-05	0.06	-	-
ENSP00000252483	PVRL2	3.14E-05	0.06	-	-
ENSP00000315602	CHRNA3	2.99E-05	0.06	-	-
ENSP00000312988	NFKBIB	2.61E-05	0.06	-	-
ENSP00000392828	GPSM1	2.18E-05	0.06	-	-
ENSP00000300417	LRSAM1	2.02E-05	0.06	-	-
ENSP00000359446	CPN1	1.48E-05	0.06	-	-
ENSP00000273145	CCBP2	1.34E-05	0.06	-	-
ENSP00000317997	DUOX1	1.03E-05	0.06	-	-
ENSP00000369889	COL2A1	6.05E-05	0.061	-	-
ENSP00000381098	GRIP1	5.85E-05	0.061	-	-
ENSP00000257497	ANXA1	5.67E-05	0.061	-	-
ENSP00000364133	TGFBR1	5.65E-05	0.061	-	-
ENSP00000306361	FGA	4.64E-05	0.061	-	-
ENSP00000296435	CAMP	4.44E-05	0.061	-	-
ENSP00000264867	PPARGC1A	3.97E-05	0.061	-	-

ENSP00000276689	NDUFB9	3.88E-05	0.061	-	-
ENSP00000262768	TIMP2	3.82E-05	0.061	-	-
ENSP00000345161	SFTPB	3.73E-05	0.061	-	-
ENSP00000399530	SKIV2L	3.71E-05	0.061	-	-
ENSP00000386200	FOXP2	3.59E-05	0.061	-	-
ENSP00000390131	NCR3	3.43E-05	0.061	-	-
ENSP00000401504	HLA-DOA	2.69E-05	0.061	-	-
ENSP00000317674	APOL1	2.15E-05	0.061	-	-
ENSP00000314879	ALOXE3	1.85E-05	0.061	-	-
ENSP00000284981	APP	1.13E-04	0.062	-	-
ENSP00000360871	FCN1	3.44E-05	0.062	-	-
ENSP00000293362	PSME3	2.91E-05	0.062	-	-
ENSP00000265131	TNC	2.87E-05	0.062	-	-
ENSP00000379475	DDX39B	2.82E-05	0.062	-	-
ENSP00000314223	GPR88	2.70E-05	0.062	-	-
ENSP00000388566	CASP4	2.46E-05	0.062	-	-
ENSP00000270474	PDE4A	6.20E-05	0.063	-	-
ENSP00000298942	PTER	5.13E-05	0.063	-	-
ENSP00000311545	EMR1	4.69E-05	0.063	-	-
ENSP00000292401	AZGP1	3.40E-05	0.063	-	-
ENSP00000353820	CYP2D6	3.37E-05	0.063	-	-
ENSP00000330393	LEPR	3.13E-05	0.063	-	-

ENSP00000330284	NPBWR1	2.90E-05	0.063	-	-
ENSP00000273951	GC	2.45E-05	0.063	-	-
ENSP00000265388	TNPO3	2.29E-05	0.063	-	-
ENSP00000311697	FGF5	2.06E-05	0.063	-	-
ENSP00000234816	ANGPTL1	1.93E-05	0.063	-	-
ENSP00000405176	TWIST2	1.36E-05	0.063	-	-
ENSP00000227507	CCND1	1.11E-04	0.064	-	-
ENSP00000373023	CDSN	5.46E-05	0.064	-	-
ENSP00000296154	CASR	5.33E-05	0.064	-	-
ENSP00000263645	CD81	5.11E-05	0.064	-	-
ENSP00000009180	CD9	4.26E-05	0.064	-	-
ENSP00000333194	RGS19	3.58E-05	0.064	-	-
ENSP00000263036	OPTN	3.48E-05	0.064	-	-
ENSP00000408581	TMEM132D	2.29E-05	0.064	-	-
ENSP00000375899	SP140	2.10E-05	0.064	-	-
ENSP00000355157	FCRL2	1.77E-05	0.064	-	-
ENSP00000331152	MXI1	1.65E-05	0.064	-	-
ENSP00000400326	DDX39B	6.27E-05	0.065	-	-
ENSP00000332225	PMCH	4.61E-05	0.065	-	-
ENSP00000353408	MSN	4.51E-05	0.065	-	-
ENSP00000356505	NCF2	4.35E-05	0.065	-	-
ENSP00000242057	AHR	4.34E-05	0.065	-	-

ENSP00000361548	MPL	3.39E-05	0.065	-	-
ENSP00000350815	NR3C2	3.14E-05	0.065	-	-
ENSP00000247270	EVI2A	2.85E-05	0.065	-	-
ENSP00000291842	SHKBP1	2.77E-05	0.065	-	-
ENSP00000229332	CLEC4A	2.41E-05	0.065	-	-
ENSP00000295117	CCDC104	2.31E-05	0.065	-	-
ENSP00000244336	CEACAM8	1.48E-05	0.065	-	-
ENSP00000300057	MESP1	1.18E-05	0.065	-	-
ENSP00000339328	PLAUR	6.15E-05	0.066	-	-
ENSP00000315878	MS4A6A	4.27E-05	0.066	-	-
ENSP00000344352	ATF3	3.73E-05	0.066	-	-
ENSP00000351908	MAP3K5	3.72E-05	0.066	-	-
ENSP00000322421	HBA1	3.18E-05	0.066	-	-
ENSP00000352455	MFAP5	2.67E-05	0.066	-	-
ENSP00000309714	SH3PXD2B	2.46E-05	0.066	-	-
ENSP00000307666	CCDC132	2.45E-05	0.066	-	-
ENSP00000244043	PTGIS	1.93E-05	0.066	-	-
ENSP00000342156	NCR3	1.66E-05	0.066	-	-
ENSP00000349616	ADIPOR2	1.26E-05	0.066	-	-
ENSP00000396538	NFAT5	5.45E-05	0.067	-	-
ENSP00000364000	COL5A2	4.59E-05	0.067	-	-
ENSP00000354822	XAF1	3.76E-05	0.067	-	-

ENSP00000272167	EPHX1	3.10E-05	0.067	-	-
ENSP00000409367	GNL1	3.08E-05	0.067	-	-
ENSP00000363431	NPY4R	2.89E-05	0.067	-	-
ENSP00000298596	STOX1	2.45E-05	0.067	-	-
ENSP00000232424	HES1	1.98E-05	0.067	-	-
ENSP00000365530	STXBP4	1.72E-05	0.067	-	-
ENSP00000367069	ZFAT	1.55E-05	0.067	-	-
ENSP00000321445	MSC	1.49E-05	0.067	-	-
ENSP00000377502	PHLDB2	1.23E-05	0.067	-	-
ENSP00000368104	BMP2	6.93E-05	0.068	-	-
ENSP00000231509	NR3C1	6.53E-05	0.068	-	-
ENSP00000241416	ACVR2A	3.36E-05	0.068	-	-
ENSP00000403576	GNL1	3.08E-05	0.068	-	-
ENSP00000312002	HAVCR2	2.36E-05	0.068	-	-
ENSP00000295756	TRAT1	2.24E-05	0.068	-	-
ENSP00000354119	LAT	2.15E-05	0.068	-	-
ENSP00000392787	TRIM27	1.81E-05	0.068	-	-
ENSP00000342667	RNF19A	1.44E-05	0.068	-	-
ENSP00000278071	ITPRIP	1.23E-05	0.068	-	-
ENSP00000363822	AR	9.71E-05	0.069	-	-
ENSP00000287727	ZFYVE9	7.57E-05	0.069	-	-
ENSP00000294724	AGL	5.97E-05	0.069	-	-

ENSP00000264110	ATF2	4.64E-05	0.069	-	-
ENSP00000366410	NMNAT1	3.56E-05	0.069	-	-
ENSP00000261714	BLMH	2.69E-05	0.069	-	-
ENSP00000268459	NKD1	2.40E-05	0.069	-	-
ENSP00000349967	CCRL2	1.94E-05	0.069	-	-
ENSP00000366682	IRG1	1.89E-05	0.069	-	-
ENSP00000337144	AQP12A	1.62E-05	0.069	-	-
ENSP00000206249	ESR1	1.43E-04	0.07	-	-
ENSP00000241052	CAT	5.96E-05	0.07	-	-
ENSP00000401303	SHC1	4.27E-05	0.07	-	-
ENSP00000375557	POU5F1B	3.85E-05	0.07	-	-
ENSP00000255262	NMUR2	3.75E-05	0.07	-	-
ENSP00000344577	APOL3	2.14E-05	0.07	-	-
ENSP00000312099	CRYGS	1.96E-05	0.07	-	-
ENSP00000355941	TATDN3	1.90E-05	0.07	-	-
ENSP00000314976	IL20RA	1.66E-05	0.07	-	-
ENSP00000336762	ANG	1.65E-05	0.07	-	-
ENSP00000312304	TPMT	1.53E-05	0.07	-	-
ENSP00000369571	IFNA14	1.41E-05	0.07	-	-
ENSP00000257267	C5AR2	1.14E-05	0.07	-	-
ENSP00000334448	GNG2	5.92E-05	0.071	-	-
ENSP00000256458	IRAK2	3.69E-05	0.071	-	-

ENSP00000249636	PIAS1	3.65E-05	0.071	-	-
ENSP00000337632	SARNP	2.44E-05	0.071	-	-
ENSP00000171757	P2RY10	2.23E-05	0.071	-	-
ENSP00000325506	CLVS1	2.17E-05	0.071	-	-
ENSP00000270632	SPIB	2.08E-05	0.071	-	-
ENSP00000261368	SNCAIP	1.80E-05	0.071	-	-
ENSP00000317357	OR13D1	1.49E-05	0.071	-	-
ENSP00000360718	RAB3B	6.31E-05	0.072	-	-
ENSP00000264218	NMU	3.86E-05	0.072	-	-
ENSP00000252029	TYMP	3.63E-05	0.072	-	-
ENSP00000376849	CASP5	2.91E-05	0.072	-	-
ENSP00000348215	SH3PXD2A	2.43E-05	0.072	-	-
ENSP00000354632	MT-ATP6	2.32E-05	0.072	-	-
ENSP00000351113	FRYL	2.16E-05	0.072	-	-
ENSP00000244519	BTN3A3	2.00E-05	0.072	-	-
ENSP00000357301	RXFP4	3.50E-05	0.073	-	-
ENSP00000366396	XRN2	3.26E-05	0.073	-	-
ENSP00000327054	LMNB2	2.86E-05	0.073	-	-
ENSP00000255688	RARRES3	2.50E-05	0.073	-	-
ENSP00000374778	IGKV4-1	1.97E-05	0.073	-	-
ENSP00000256447	CD180	1.72E-05	0.073	-	-
ENSP00000267890	TTBK2	1.53E-05	0.073	-	-

ENSP00000341905	RASAL3	1.41E-05	0.073	-	-
ENSP00000412800	DDAH2	1.04E-05	0.073	-	-
ENSP00000340858	B2M	2.12E-04	0.074	-	-
ENSP00000052754	DCN	7.47E-05	0.074	-	-
ENSP00000348234	TAT	4.78E-05	0.074	-	-
ENSP00000376436	ETS1	4.20E-05	0.074	-	-
ENSP00000257857	CD63	3.53E-05	0.074	-	-
ENSP00000355520	RGS7	2.93E-05	0.074	-	-
ENSP00000381823	SEC24A	2.82E-05	0.074	-	-
ENSP00000264758	ADD1	2.17E-05	0.074	-	-
ENSP00000320503	EDC3	2.15E-05	0.074	-	-
ENSP00000344967	SPRY4	2.09E-05	0.074	-	-
ENSP00000341030	CSN2	1.72E-05	0.074	-	-
ENSP00000343891	NLRP13	1.37E-05	0.074	-	-
ENSP00000220597	PAG1	1.26E-05	0.074	-	-
ENSP00000361125	VEGFA	2.59E-04	0.075	-	-
ENSP00000358548	NRAS	7.91E-05	0.075	-	-
ENSP00000368683	EDN1	5.46E-05	0.075	-	-
ENSP00000275525	IGFBP1	4.58E-05	0.075	-	-
ENSP00000263826	AKT3	4.00E-05	0.075	-	-
ENSP00000402316	TAP1	3.72E-05	0.075	-	-
ENSP00000385057	APOBEC3G	3.46E-05	0.075	-	-

ENSP00000357794	TCHH	3.11E-05	0.075	-	-
ENSP00000372155	PSME1	2.78E-05	0.075	-	-
ENSP00000364683	BAMBI	2.36E-05	0.075	-	-
ENSP00000367608	CA9	2.31E-05	0.075	-	-
ENSP00000230568	LY86	4.61E-05	0.076	-	-
ENSP00000231004	LOX	4.47E-05	0.076	-	-
ENSP00000407375	GPX1	3.91E-05	0.076	-	-
ENSP00000358165	FCGR1A	3.42E-05	0.076	-	-
ENSP00000365175	PRRC2A	3.28E-05	0.076	-	-
ENSP00000264474	CSTA	2.71E-05	0.076	-	-
ENSP00000269980	BCKDHA	2.66E-05	0.076	-	-
ENSP00000259254	GYPC	2.51E-05	0.076	-	-
ENSP00000310832	CTSF	1.11E-05	0.076	-	-
ENSP00000220809	PLAT	6.15E-05	0.077	-	-
ENSP00000261799	PDGFRB	5.87E-05	0.077	-	-
ENSP00000357494	ROS1	4.58E-05	0.077	-	-
ENSP00000358541	SIKE1	4.19E-05	0.077	-	-
ENSP00000324248	PENK	3.67E-05	0.077	-	-
ENSP00000363998	ITCH	3.14E-05	0.077	-	-
ENSP00000222553	NAMPT	2.85E-05	0.077	-	-
ENSP00000216214	FAM118A	1.69E-05	0.077	-	-
ENSP00000287482	SASS6	1.65E-05	0.077	-	-

ENSP00000393996	AIF1	1.46E-05	0.077	-	-
ENSP00000219535	FAM173A	1.27E-05	0.077	-	-
ENSP00000358867	GNAI3	5.88E-05	0.078	-	-
ENSP00000395337	LDHA	4.14E-05	0.078	-	-
ENSP00000215479	AMELY	4.01E-05	0.078	-	-
ENSP00000218758	ACP5	3.18E-05	0.078	-	-
ENSP00000331614	IKZF1	2.71E-05	0.078	-	-
ENSP00000267101	ERBB3	5.50E-05	0.079	-	-
ENSP00000217305	PDYN	4.00E-05	0.079	-	-
ENSP00000340347	TCF7	3.44E-05	0.079	-	-
ENSP00000363031	THEMIS2	3.22E-05	0.079	-	-
ENSP00000333268	CSH1	2.52E-05	0.079	-	-
ENSP00000402460	CLSTN2	1.55E-05	0.079	-	-
ENSP00000391116	NBPF12	1.42E-05	0.079	-	-
ENSP00000349181	HVCN1	1.37E-05	0.079	-	-
ENSP00000383516	HMX1	1.07E-05	0.079	-	-
ENSP00000220772	SFRP1	3.37E-05	0.08	-	-
ENSP00000358203	NBPF20	2.53E-05	0.08	-	-
ENSP00000281317	MMAA	1.70E-05	0.08	-	-
ENSP00000349723	BCL11B	1.39E-05	0.08	-	-
ENSP00000254691	CARD6	1.32E-05	0.08	-	-
ENSP00000343925	ESR2	8.13E-05	0.081	-	-

ENSP00000252034	ELN	5.91E-05	0.081	-	-
ENSP00000389875	PSORS1C1	3.02E-05	0.081	-	-
ENSP00000254325	RFX1	2.92E-05	0.081	-	-
ENSP00000328708	RXFP3	2.88E-05	0.081	-	-
ENSP00000336630	ADORA2A	2.65E-05	0.081	-	-
ENSP00000362446	ZNF79	2.13E-05	0.081	-	-
ENSP00000366863	TBC1D4	2.05E-05	0.081	-	-
ENSP00000388910	NFATC4	1.79E-05	0.081	-	-
ENSP00000406250	HLA-DPA1	1.53E-05	0.081	-	-
ENSP00000329568	NLRP7	1.27E-05	0.081	-	-
ENSP00000228872	CDKN1B	5.51E-05	0.082	-	-
ENSP00000329623	BCL2	5.36E-05	0.082	-	-
ENSP00000399604	CDSN	5.02E-05	0.082	-	-
ENSP00000348611	PHLPP2	3.89E-05	0.082	-	-
ENSP00000290122	CELA3A	2.85E-05	0.082	-	-
ENSP00000334052	LGMN	2.56E-05	0.082	-	-
ENSP00000246006	CD93	2.36E-05	0.082	-	-
ENSP00000225387	CRYBA1	2.00E-05	0.082	-	-
ENSP00000345317	CXCL17	1.15E-05	0.082	-	-
ENSP00000369564	IFNA16	1.03E-05	0.082	-	-
ENSP00000254351	SDC1	4.70E-05	0.083	-	-
ENSP00000241125	GJA3	3.52E-05	0.083	-	-

ENSP00000415363	PRRC2A	3.09E-05	0.083	-	-
ENSP00000394400	SKIV2L	3.09E-05	0.083	-	-
ENSP00000399542	DDX39B	2.92E-05	0.083	-	-
ENSP00000231656	CDX1	2.78E-05	0.083	-	-
ENSP00000363377	FOXO4	2.51E-05	0.083	-	-
ENSP00000338648	MS4A4A	1.49E-05	0.083	-	-
ENSP00000372901	DDAH2	1.09E-05	0.083	-	-
ENSP00000244741	CDKN1A	8.55E-05	0.084	-	-
ENSP00000277480	LCN2	3.87E-05	0.084	-	-
ENSP00000234091	ID2	3.70E-05	0.084	-	-
ENSP00000294339	TAL1	3.27E-05	0.084	-	-
ENSP00000248901	CYTH4	2.93E-05	0.084	-	-
ENSP00000337053	SEL1L	2.93E-05	0.084	-	-
ENSP00000230882	GHR	2.74E-05	0.084	-	-
ENSP00000389207	NOTCH4	2.36E-05	0.084	-	-
ENSP00000270139	IFNAR1	3.76E-05	0.085	-	-
ENSP00000293330	HCRT	3.59E-05	0.085	-	-
ENSP00000379158	CAST	3.24E-05	0.085	-	-
ENSP00000401321	NOTCH4	2.48E-05	0.085	-	-
ENSP00000372751	NOTCH4	2.36E-05	0.085	-	-
ENSP00000367631	ANKEF1	2.32E-05	0.085	-	-
ENSP00000266066	SFRP5	2.22E-05	0.085	-	-

ENSP00000261454	PROX1	2.05E-05	0.085	-	-
ENSP00000276420	DOK2	1.55E-05	0.085	-	-
ENSP00000368766	ADRA1D	3.66E-05	0.086	-	-
ENSP00000362410	MAFB	2.83E-05	0.086	-	-
ENSP00000216274	RIPK3	1.57E-05	0.086	-	-
ENSP00000251973	CARD10	1.41E-05	0.086	-	-
ENSP00000359013	TNFRSF6B	1.24E-05	0.086	-	-
ENSP00000364252	PLA2G2A	5.55E-05	0.087	-	-
ENSP00000241651	MYOG	4.64E-05	0.087	-	-
ENSP00000254322	DNAJB1	3.43E-05	0.087	-	-
ENSP00000225992	PPY	3.10E-05	0.087	-	-
ENSP00000302811	MTNR1A	2.93E-05	0.087	-	-
ENSP00000215939	CRYBB1	2.20E-05	0.087	-	-
ENSP00000366031	CST7	2.18E-05	0.087	-	-
ENSP00000356630	HECA	1.76E-05	0.087	-	-
ENSP00000388107	UBA52	1.83E-04	0.088	-	-
ENSP00000216336	CTSG	3.72E-05	0.088	-	-
ENSP00000274063	SFRP2	2.92E-05	0.088	-	-
ENSP00000268150	MFGE8	2.36E-05	0.088	-	-
ENSP00000296125	TGM4	1.19E-05	0.088	-	-
ENSP00000307272	RPTOR	3.19E-05	0.089	-	-
ENSP00000233813	IGFBP5	2.69E-05	0.089	-	-

ENSP00000234831	TMEM59	2.40E-05	0.089	-	-
ENSP00000331066	OLIG1	2.30E-05	0.089	-	-
ENSP00000345393	MAFF	2.14E-05	0.089	-	-
ENSP00000396251	GTF2H4	2.08E-05	0.089	-	-
ENSP00000357791	HRNR	1.71E-05	0.089	-	-
ENSP00000348632	RBCK1	1.47E-05	0.089	-	-
ENSP00000295006	CAPN2	1.30E-05	0.089	-	-
ENSP00000360286	RAE1	4.97E-05	0.09	-	-
ENSP00000419425	PPIA	4.10E-05	0.09	-	-
ENSP00000326247	SAMD9L	3.96E-05	0.09	-	-
ENSP00000320401	UGT2B17	3.17E-05	0.09	-	-
ENSP00000259895	GTF2H4	2.09E-05	0.09	-	-
ENSP00000331698	KCNJ15	1.63E-05	0.09	-	-
ENSP00000367714	HES5	3.46E-05	0.091	-	-
ENSP00000314733	TOLLIP	3.20E-05	0.091	-	-
ENSP00000229330	HCFC2	2.05E-05	0.091	-	-
ENSP00000347979	FAS	1.77E-05	0.091	-	-
ENSP00000344549	CARD14	1.37E-05	0.091	-	-
ENSP00000412309	SLC14A1	1.11E-05	0.091	-	-
ENSP00000360125	PGM1	3.05E-05	0.092	-	-
ENSP00000310170	FOSL1	2.87E-05	0.092	-	-
ENSP00000394049	SLA	2.43E-05	0.092	-	-

ENSP00000388322	HHLA1	2.00E-05	0.092	-	-
ENSP00000239223	DUSP1	1.83E-05	0.092	-	-
ENSP00000374552	RNF216	1.27E-05	0.092	-	-
ENSP00000270879	FCN3	1.07E-05	0.092	-	-
ENSP00000401272	GPANK1	4.43E-05	0.093	-	-
ENSP00000386444	SAG	3.04E-05	0.093	-	-
ENSP00000354335	MPEG1	2.90E-05	0.093	-	-
ENSP00000357033	CD84	1.53E-05	0.093	-	-
ENSP00000264036	MCAM	1.38E-05	0.093	-	-
ENSP00000401548	TCF19	3.23E-05	0.094	-	-
ENSP00000316244	HTR1A	3.20E-05	0.094	-	-
ENSP00000298910	LRRK2	3.10E-05	0.094	-	-
ENSP00000368686	E2F4	2.94E-05	0.094	-	-
ENSP00000348099	PDLIM7	2.74E-05	0.094	-	-
ENSP00000330005	RGMA	2.45E-05	0.094	-	-
ENSP00000222286	GAPDHS	2.31E-05	0.094	-	-
ENSP00000267012	BIN2	2.04E-05	0.094	-	-
ENSP00000374855	IGLC1	1.71E-05	0.094	-	-
ENSP00000384053	CSF2RB	1.41E-05	0.094	-	-
ENSP00000364694	ASPN	4.31E-05	0.095	-	-
ENSP00000007722	ITGA3	3.49E-05	0.095	-	-
ENSP00000215909	LGALS1	3.42E-05	0.095	-	-

ENSP00000265983	HPX	3.33E-05	0.095	-	-
ENSP00000367124	SLC3A2	3.24E-05	0.095	-	-
ENSP00000382423	MAP3K1	3.12E-05	0.095	-	-
ENSP00000294489	PDPN	2.76E-05	0.095	-	-
ENSP00000297991	AQP3	2.33E-05	0.095	-	-
ENSP00000354607	FZD5	2.13E-05	0.095	-	-
ENSP00000372313	MSLN	1.57E-05	0.095	-	-
ENSP00000341551	SMAD4	8.20E-05	0.096	-	-
ENSP00000291700	S100B	3.43E-05	0.096	-	-
ENSP00000305152	PLP1	2.79E-05	0.096	-	-
ENSP00000294338	PDZK1IP1	2.16E-05	0.096	-	-
ENSP00000346805	CRYBA4	1.73E-05	0.096	-	-
ENSP00000335203	ATPIF1	1.71E-05	0.096	-	-
ENSP00000362469	SLC2A8	1.30E-05	0.096	-	-
ENSP00000331857	NLRP9	1.17E-05	0.096	-	-
ENSP00000324648	CYP2B6	6.23E-05	0.097	-	-
ENSP00000338297	IGF2	4.72E-05	0.097	-	-
ENSP00000280551	SEC24D	2.80E-05	0.097	-	-
ENSP00000323901	FZD2	2.44E-05	0.097	-	-
ENSP00000325589	PTPRCAP	1.90E-05	0.097	-	-
ENSP00000356581	GPR126	1.57E-05	0.097	-	-
ENSP00000266839	MMAB	1.52E-05	0.097	-	-

ENSP00000343339	P2RX2	1.52E-05	0.097	-	-
ENSP00000415900	IL31RA	1.17E-05	0.097	-	-
ENSP00000340330	KAT5	4.25E-05	0.098	-	-
ENSP00000352157	MAPK10	3.96E-05	0.098	-	-
ENSP00000253054	GMFG	2.85E-05	0.098	-	-
ENSP00000292199	NLRX1	2.52E-05	0.098	-	-
ENSP00000221498	DKKL1	2.44E-05	0.098	-	-
ENSP00000316152	SFTPC	2.34E-05	0.098	-	-
ENSP00000274217	FAM105A	1.97E-05	0.098	-	-
ENSP00000354691	FCRL5	1.92E-05	0.098	-	-
ENSP00000418901	SYT14	1.85E-05	0.098	-	-
ENSP00000260197	SORL1	1.73E-05	0.098	-	-
ENSP00000262177	DNAJB6	1.50E-05	0.098	-	-
ENSP00000318770	AQP11	1.28E-05	0.098	-	-
ENSP00000289932	SLC5A11	1.24E-05	0.098	-	-
ENSP00000357283	LMNA	5.17E-05	0.099	-	-
ENSP00000369127	DNAJA1	3.08E-05	0.099	-	-
ENSP00000406965	ABHD16A	3.07E-05	0.099	-	-
ENSP00000300108	TAC3	2.28E-05	0.099	-	-
ENSP00000346402	RNH1	2.26E-05	0.099	-	-
ENSP00000370297	CER1	1.96E-05	0.099	-	-
ENSP00000385461	TFDP3	1.63E-05	0.099	-	-

ENSP00000351209	EPHA2	3.65E-05	0.1	-	-
ENSP00000249016	MCHR1	3.57E-05	0.1	-	-
ENSP00000226299	LAP3	3.56E-05	0.1	-	-
ENSP00000357711	S100A7	2.95E-05	0.1	-	-
ENSP00000322304	PORCN	2.58E-05	0.1	-	-
ENSP00000356037	C4BPA	2.45E-05	0.1	-	-
ENSP00000364163	NOTCH4	2.37E-05	0.1	-	-
ENSP00000262878	SAMHD1	2.04E-05	0.1	-	-
ENSP00000286186	CASP10	1.81E-05	0.1	-	-
ENSP00000312741	CAMKK2	1.71E-05	0.1	-	-
ENSP00000342538	STMN4	1.41E-05	0.1	-	-
ENSP00000323568	SLC2A2	3.33E-05	0.101	-	-
ENSP00000304858	ORMDL3	3.24E-05	0.101	-	-
ENSP00000261292	LIPG	3.13E-05	0.101	-	-
ENSP00000346829	WLS	2.32E-05	0.101	-	-
ENSP00000264553	GZMM	1.68E-05	0.101	-	-
ENSP00000391372	SH3GL3	1.58E-05	0.101	-	-
ENSP00000279463	CNTN5	1.11E-05	0.101	-	-
ENSP00000359910	PSMA7	3.39E-05	0.102	-	-
ENSP00000269485	TNFRSF11A	3.35E-05	0.102	-	-
ENSP00000405229	TRIM27	2.48E-05	0.102	-	-
ENSP00000364893	ARHGEF7	2.44E-05	0.102	-	-

ENSP00000320516	EHD1	2.41E-05	0.102	-	-
ENSP00000286201	FZD7	2.31E-05	0.102	-	-
ENSP00000310800	VANGL1	2.17E-05	0.102	-	-
ENSP00000217423	CST4	1.81E-05	0.102	-	-
ENSP00000296280	MASP1	1.63E-05	0.102	-	-
ENSP00000262659	CCM2L	1.41E-05	0.102	-	-
ENSP00000223271	RARRES2	1.37E-05	0.102	-	-
ENSP00000254301	LGALS3	2.49E-05	0.103	-	-
ENSP00000226247	FOXN1	2.38E-05	0.103	-	-
ENSP00000328169	JAG2	2.36E-05	0.103	-	-
ENSP00000262101	MSR1	2.27E-05	0.103	-	-
ENSP00000354927	MAP3K3	2.21E-05	0.103	-	-
ENSP00000258349	RC3H1	1.55E-05	0.103	-	-
ENSP00000215855	CRYBB3	1.52E-05	0.103	-	-
ENSP00000221740	CASP14	1.51E-05	0.103	-	-
ENSP00000256785	CFHR5	1.43E-05	0.103	-	-
ENSP00000362013	TRERF1	1.39E-05	0.103	-	-
ENSP00000361021	PTEN	1.07E-04	0.104	-	-
ENSP00000246792	RRAS	4.61E-05	0.104	-	-
ENSP00000379213	PTHLH	3.72E-05	0.104	-	-
ENSP00000229335	AICDA	3.27E-05	0.104	-	-
ENSP00000218099	F9	2.65E-05	0.104	-	-

ENSP00000310126	LINGO2	2.39E-05	0.104	-	-
ENSP00000370227	AREGB	1.86E-05	0.104	-	-
ENSP00000312652	LEP	1.26E-04	0.105	-	-
ENSP00000265724	ABCB1	4.86E-05	0.105	-	-
ENSP00000308176	BTK	4.45E-05	0.105	-	-
ENSP00000293379	ITGA5	3.58E-05	0.105	-	-
ENSP00000262776	LGALS3BP	2.11E-05	0.105	-	-
ENSP00000261623	CYBA	1.80E-05	0.105	-	-
ENSP00000303507	BCR	5.31E-05	0.106	-	-
ENSP00000323036	ACPP	4.80E-05	0.106	-	-
ENSP00000352575	INPP5D	3.83E-05	0.106	-	-
ENSP00000347409	KEL	2.67E-05	0.106	-	-
ENSP00000253496	F12	1.81E-05	0.106	-	-
ENSP00000382707	IFITM3	1.39E-05	0.106	-	-
ENSP00000317087	8-Mar	1.21E-05	0.106	-	-
ENSP00000310880	MAL	1.13E-05	0.106	-	-
ENSP00000390725	HLA-DQA2	1.02E-05	0.106	-	-
ENSP00000405833	HLA-DQA2	1.02E-05	0.106	-	-
ENSP00000245451	BMP4	8.15E-05	0.107	-	-
ENSP00000247668	TRAF2	4.64E-05	0.107	-	-
ENSP00000219700	HMOX2	4.22E-05	0.107	-	-
ENSP00000367830	PRKCZ	3.77E-05	0.107	-	-

ENSP00000376544	CD79B	2.42E-05	0.107	-	-
ENSP00000217182	EEF1A2	2.37E-05	0.107	-	-
ENSP00000225941	ABI3	2.27E-05	0.107	-	-
ENSP00000264487	AREG	1.88E-05	0.107	-	-
ENSP00000338814	BAG5	1.80E-05	0.107	-	-
ENSP00000262971	PIAS4	1.78E-05	0.107	-	-
ENSP00000240617	PLBD1	1.68E-05	0.107	-	-
ENSP00000393198	HES4	1.61E-05	0.107	-	-
ENSP00000380514	HAGH	1.41E-05	0.107	-	-
ENSP00000288422	TAB3	1.33E-05	0.107	-	-
ENSP00000367130	HES3	1.32E-05	0.107	-	-
ENSP00000400695	HLA-DQA2	1.02E-05	0.107	-	-
ENSP00000304697	UBB	8.21E-05	0.108	-	-
ENSP00000375892	AKT2	5.29E-05	0.108	-	-
ENSP00000365466	PSORS1C1	2.75E-05	0.108	-	-
ENSP00000361366	SFTPD	2.50E-05	0.108	-	-
ENSP00000358106	PREP	1.89E-05	0.108	-	-
ENSP00000256196	RRAS2	4.23E-05	0.109	-	-
ENSP00000330237	CASP9	3.73E-05	0.109	-	-
ENSP00000240986	GJA8	3.41E-05	0.109	-	-
ENSP00000299166	NDUFB8	3.22E-05	0.109	-	-
ENSP00000306881	SEC23A	2.93E-05	0.109	-	-

ENSP00000364731	F7	2.21E-05	0.109	-	-
ENSP00000321674	4-Sep	1.97E-05	0.109	-	-
ENSP00000262395	TRAF4	1.74E-05	0.109	-	-
ENSP00000291565	PDXK	1.68E-05	0.109	-	-
ENSP00000287641	SST	6.24E-05	0.11	-	-
ENSP00000400047	POU5F1	3.92E-05	0.11	-	-
ENSP00000329797	CADM1	2.64E-05	0.11	-	-
ENSP00000358126	VPS45	2.30E-05	0.11	-	-
ENSP00000357726	S100A12	2.15E-05	0.11	-	-
ENSP00000304987	BFSP2	2.01E-05	0.11	-	-
ENSP00000366404	TRIM27	1.83E-05	0.11	-	-
ENSP00000357656	FYN	1.00E-04	0.111	-	-
ENSP00000176183	DRD4	3.39E-05	0.111	-	-
ENSP00000346103	GPX4	3.32E-05	0.111	-	-
ENSP00000249041	GALR3	2.91E-05	0.111	-	-
ENSP00000295381	ARHGAP25	2.58E-05	0.111	-	-
ENSP00000164227	BCL3	2.38E-05	0.111	-	-
ENSP00000327943	SLC5A2	1.94E-05	0.111	-	-
ENSP00000349873	HIST1H2AL	1.32E-05	0.111	-	-
ENSP00000335076	EDARADD	1.17E-05	0.111	-	-
ENSP00000358421	HSD3B1	3.44E-05	0.112	-	-
ENSP00000230301	C6orf118	3.03E-05	0.112	-	-

ENSP00000249749	DLL4	2.50E-05	0.112	-	-
ENSP00000251390	LILRA3	2.08E-05	0.112	-	-
ENSP00000280097	HNMT	1.84E-05	0.112	-	-
ENSP00000369218	RBM17	1.69E-05	0.112	-	-
ENSP00000367104	BFSP1	1.52E-05	0.112	-	-
ENSP00000275169	GPR6	1.45E-05	0.112	-	-
ENSP00000346467	LTBP1	1.20E-05	0.112	-	-
ENSP00000257068	MTNR1B	3.06E-05	0.113	-	-
ENSP00000285407	KLF10	2.67E-05	0.113	-	-
ENSP00000288167	IL17RB	1.69E-05	0.113	-	-
ENSP00000265162	ENPEP	1.39E-05	0.113	-	-
ENSP00000292176	ZBTB7B	1.14E-05	0.113	-	-
ENSP00000302176	ASPSCR1	3.02E-05	0.114	-	-
ENSP00000304111	ABCG4	2.85E-05	0.114	-	-
ENSP00000286835	SCAF4	2.25E-05	0.114	-	-
ENSP00000305529	SIRPG	2.10E-05	0.114	-	-
ENSP00000291568	CSTB	2.02E-05	0.114	-	-
ENSP00000353072	ATP2A3	2.00E-05	0.114	-	-
ENSP00000356248	PTPN7	1.70E-05	0.114	-	-
ENSP00000221448	SNRNP70	3.30E-05	0.115	-	-
ENSP00000352936	SPINK5	2.81E-05	0.115	-	-
ENSP00000267843	FGF7	2.80E-05	0.115	-	-

ENSP00000371790	FGF9	2.18E-05	0.115	-	-
ENSP00000376623	CSH2	1.89E-05	0.115	-	-
ENSP00000357594	FAM26F	1.76E-05	0.115	-	-
ENSP00000380598	BLOC1S5	1.67E-05	0.115	-	-
ENSP00000349275	NRG1	3.39E-05	0.116	-	-
ENSP00000373715	DCP2	2.78E-05	0.116	-	-
ENSP00000370697	P2RY8	1.45E-05	0.116	-	-
ENSP00000357158	FCRL1	1.31E-05	0.116	-	-
ENSP00000369666	FBXL19	1.15E-05	0.116	-	-
ENSP00000339692	RASSF4	1.03E-05	0.116	-	-
ENSP00000261340	CLEC2D	1.02E-05	0.116	-	-
ENSP00000353059	APAF1	4.63E-05	0.117	-	-
ENSP00000256857	GRP	3.27E-05	0.117	-	-
ENSP00000260408	ADAM10	2.98E-05	0.117	-	-
ENSP00000364126	APEX2	2.29E-05	0.117	-	-
ENSP00000337946	PACRG	2.08E-05	0.117	-	-
ENSP00000262031	RBMS2	1.50E-05	0.117	-	-
ENSP00000358470	VTCN1	1.15E-05	0.117	-	-
ENSP00000241802	HLA-DQA2	1.02E-05	0.117	-	-
ENSP00000315167	ALOX12B	2.09E-05	0.118	-	-
ENSP00000356382	F13B	2.02E-05	0.118	-	-
ENSP00000257600	DTX1	1.56E-05	0.118	-	-

ENSP00000271532	FCRL4	1.52E-05	0.118	-	-
ENSP00000161559	CEACAM1	1.36E-05	0.118	-	-
ENSP00000320176	HCLS1	4.10E-05	0.119	-	-
ENSP00000318486	CDK5R1	2.77E-05	0.119	-	-
ENSP00000337128	EDN3	2.67E-05	0.119	-	-
ENSP00000398278	PSORS1C1	2.64E-05	0.119	-	-
ENSP00000221973	LIM2	2.21E-05	0.119	-	-
ENSP00000364336	TBXA2R	2.19E-05	0.119	-	-
ENSP00000372991	LTA	2.05E-05	0.119	-	-
ENSP00000264376	CRYGD	1.58E-05	0.119	-	-
ENSP00000253719	NAPSA	1.09E-05	0.119	-	-
ENSP00000310275	BANF1	3.86E-05	0.12	-	-
ENSP00000240093	FZD3	1.90E-05	0.12	-	-
ENSP00000358795	NEURL	1.41E-05	0.12	-	-
ENSP00000385610	MEX3C	1.36E-05	0.12	-	-
ENSP00000342830	RDX	3.38E-05	0.121	-	-
ENSP00000341785	ADIPOR1	1.65E-05	0.121	-	-
ENSP00000351589	HIST1H2AI	1.35E-05	0.121	-	-
ENSP00000329920	C19orf59	1.26E-05	0.121	-	-
ENSP00000275493	EGFR	1.34E-04	0.122	-	-
ENSP00000297436	DEFA6	2.29E-05	0.122	-	-
ENSP00000367609	PABPN1L	1.65E-05	0.122	-	-

ENSP00000293288	BAX	4.78E-05	0.123	-	-
ENSP00000272937	HES6	1.69E-05	0.123	-	-
ENSP00000352798	COL18A1	3.22E-05	0.124	-	-
ENSP00000337949	HTR7	1.95E-05	0.124	-	-
ENSP00000408395	RBFOX3	1.84E-05	0.124	-	-
ENSP00000227638	PANX1	1.41E-05	0.124	-	-
ENSP00000330307	HIST1H2AK	1.35E-05	0.124	-	-
ENSP00000301873	LTBP3	1.09E-05	0.124	-	-
ENSP00000227155	CD82	2.94E-05	0.125	-	-
ENSP00000371798	FSCN1	2.89E-05	0.125	-	-
ENSP00000329890	DEFA5	2.65E-05	0.125	-	-
ENSP00000307706	MT1E	1.54E-05	0.125	-	-
ENSP00000260630	CYP1B1	5.10E-05	0.126	-	-
ENSP00000381293	NSF	4.75E-05	0.126	-	-
ENSP00000373106	RNF39	2.74E-05	0.126	-	-
ENSP00000373700	ALK	2.68E-05	0.126	-	-
ENSP00000263317	NOX4	1.75E-05	0.126	-	-
ENSP00000356146	SCAF8	2.61E-05	0.127	-	-
ENSP00000354929	NOTCH2NL	5.78E-05	0.128	-	-
ENSP00000250457	EGLN3	2.03E-05	0.128	-	-
ENSP00000245479	SOX9	5.11E-05	0.129	-	-
ENSP00000262965	TCF3	4.78E-05	0.129	-	-

ENSP00000360683	PTPN1	3.84E-05	0.129	-	-
ENSP00000359787	IFI44L	2.23E-05	0.129	-	-
ENSP00000351605	FZD6	2.13E-05	0.129	-	-
ENSP00000327116	EHD3	1.30E-05	0.129	-	-
ENSP00000217740	RNF125	1.03E-05	0.129	-	-
ENSP00000263025	MAPK3	9.11E-05	0.13	-	-
ENSP00000358525	NGF	8.69E-05	0.13	-	-
ENSP00000266000	DAXX	2.56E-05	0.13	-	-
ENSP00000250018	TPH1	2.51E-05	0.13	-	-
ENSP00000401369	ASB10	1.38E-05	0.13	-	-
ENSP00000363433	GPRIN2	1.21E-05	0.13	-	-
ENSP00000222254	PIK3R2	4.30E-05	0.131	-	-
ENSP00000354859	DRD2	4.23E-05	0.131	-	-
ENSP00000268607	MAP1LC3B	2.92E-05	0.131	-	-
ENSP00000361818	SDC4	2.92E-05	0.131	-	-
ENSP00000389813	ACAD10	1.69E-05	0.131	-	-
ENSP00000339933	PKLR	4.42E-05	0.132	-	-
ENSP00000350844	PAX5	3.68E-05	0.132	-	-
ENSP00000321326	F2R	3.58E-05	0.132	-	-
ENSP00000375035	IGHV5-51	1.99E-05	0.132	-	-
ENSP00000308452	KRT17	1.96E-05	0.132	-	-
ENSP00000348283	WWP2	1.85E-05	0.132	-	-

ENSP00000225983	HDAC5	3.51E-05	0.133	-	-
ENSP00000262613	SLC9A3R1	3.09E-05	0.133	-	-
ENSP00000242338	CNTFR	1.77E-05	0.133	-	-
ENSP00000338358	SERPINB6	1.43E-05	0.133	-	-
ENSP00000381486	NOTO	1.04E-05	0.133	-	-
ENSP00000313731	PLCD3	2.95E-05	0.134	-	-
ENSP00000342392	MESP2	1.78E-05	0.134	-	-
ENSP00000320447	NR2C2	1.46E-05	0.134	-	-
ENSP00000220764	DECR1	6.46E-05	0.135	-	-
ENSP00000321845	SEC24C	2.87E-05	0.135	-	-
ENSP00000362570	FNDC5	1.96E-05	0.135	-	-
ENSP00000365103	DAOA	1.32E-05	0.135	-	-
ENSP00000355133	NOS1AP	1.28E-05	0.135	-	-
ENSP00000354032	BSCL2	1.23E-05	0.135	-	-
ENSP00000333203	SERPINA5	2.09E-05	0.136	-	-
ENSP00000299502	SERPINB2	1.34E-05	0.136	-	-
ENSP00000249598	GDF2	1.27E-05	0.136	-	-
ENSP00000308541	F2	6.54E-05	0.137	-	-
ENSP00000383411	SAMSN1	1.75E-05	0.137	-	-
ENSP00000236938	FCRLA	1.31E-05	0.137	-	-
ENSP00000300209	METTL21B	1.31E-05	0.137	-	-
ENSP00000360635	DPP7	1.04E-05	0.137	-	-

ENSP00000287820	PPARG	8.12E-05	0.138	-	-
ENSP00000414303	BDNF	6.32E-05	0.138	-	-
ENSP00000245919	FOSB	2.57E-05	0.138	-	-
ENSP00000288071	DDX19B	2.45E-05	0.138	-	-
ENSP00000304501	ADORA2B	1.98E-05	0.138	-	-
ENSP00000338934	EZR	4.98E-05	0.139	-	-
ENSP00000227880	SLC15A3	3.76E-05	0.139	-	-
ENSP00000319984	CHRM2	3.08E-05	0.139	-	-
ENSP00000291554	CRYAA	2.74E-05	0.139	-	-
ENSP00000269300	PIK3R5	2.33E-05	0.139	-	-
ENSP00000256178	LYVE1	2.26E-05	0.139	-	-
ENSP00000282849	ADAMTS18	1.33E-05	0.139	-	-
ENSP00000215659	MAPK12	2.78E-05	0.14	-	-
ENSP00000284719	OLA1	2.47E-05	0.14	-	-
ENSP00000234071	PROC	2.05E-05	0.14	-	-
ENSP00000328405	DGKA	1.61E-05	0.14	-	-
ENSP00000264790	MMRN1	1.58E-05	0.14	-	-
ENSP00000379891	AP1S3	1.06E-05	0.14	-	-
ENSP00000301295	NLRP4	1.03E-05	0.14	-	-
ENSP00000172229	NGFR	3.72E-05	0.141	-	-
ENSP00000231061	SPARC	3.56E-05	0.141	-	-
ENSP00000320758	NOS1	2.97E-05	0.141	-	-

ENSP00000352802	WIPF1	1.96E-05	0.141	-	-
ENSP00000280358	TEX12	1.27E-05	0.141	-	-
ENSP00000303315	JUNB	3.50E-05	0.142	-	-
ENSP00000205948	APOH	3.42E-05	0.142	-	-
ENSP00000295095	ARHGAP15	2.70E-05	0.142	-	-
ENSP00000308610	GPD2	2.36E-05	0.142	-	-
ENSP00000384442	CDK11A	1.97E-05	0.142	-	-
ENSP00000267169	DIABLO	1.87E-05	0.142	-	-
ENSP00000256412	ADAMDEC1	1.39E-05	0.142	-	-
ENSP00000381436	VGLL3	1.11E-05	0.142	-	-
ENSP00000332326	TNFAIP2	1.09E-05	0.142	-	-
ENSP00000289902	FCER1G	3.95E-05	0.143	-	-
ENSP00000283195	RANBP2	3.01E-05	0.143	-	-
ENSP00000393007	TCF19	1.65E-05	0.143	-	-
ENSP00000256474	VHL	2.89E-05	0.144	-	-
ENSP00000345785	FZD9	1.90E-05	0.144	-	-
ENSP00000361458	TSC22D3	1.37E-05	0.144	-	-
ENSP00000339730	THBS4	1.68E-05	0.145	-	-
ENSP00000261879	APH1B	1.59E-05	0.145	-	-
ENSP00000380066	MAP4K1	1.42E-05	0.145	-	-
ENSP00000342385	PTGES	1.11E-05	0.145	-	-
ENSP00000369636	PXDC1	1.03E-05	0.145	-	-

ENSP00000317272	MET	4.01E-05	0.146	-	-
ENSP00000321853	SERPINF2	3.37E-05	0.146	-	-
ENSP00000340305	UBE2V1	1.63E-05	0.146	-	-
ENSP00000344115	CDH5	3.26E-05	0.147	-	-
ENSP00000296452	BSN	2.22E-05	0.147	-	-
ENSP00000382213	DSPP	1.77E-05	0.147	-	-
ENSP00000262366	GLIS2	1.50E-05	0.147	-	-
ENSP00000360997	ADAMTS13	1.23E-05	0.147	-	-
ENSP00000315112	RNF31	1.12E-05	0.147	-	-
ENSP00000260643	PREB	2.50E-05	0.148	-	-
ENSP00000252491	APOC1	1.53E-05	0.148	-	-
ENSP00000350369	MAFG	1.06E-05	0.148	-	-
ENSP00000340523	NMNAT3	2.42E-05	0.149	-	-
ENSP00000403447	NOTCH4	2.21E-05	0.149	-	-
ENSP00000291294	PTGIR	1.75E-05	0.149	-	-
ENSP00000301653	KRT16	1.66E-05	0.149	-	-
ENSP00000371693	TNFRSF19	1.45E-05	0.149	-	-
ENSP00000297770	CPA6	1.20E-05	0.149	-	-
ENSP00000234313	PLEK	7.59E-05	0.15	-	-
ENSP00000295731	IHH	4.42E-05	0.15	-	-
ENSP00000305372	ADRB2	3.32E-05	0.15	-	-
ENSP00000276594	PRDM14	1.61E-05	0.15	-	-

ENSP00000349629	LRBA	1.56E-05	0.15	-	-
ENSP00000160740	CIC	1.32E-05	0.15	-	-
ENSP00000347329	SEC31A	2.81E-05	0.151	-	-
ENSP00000254667	PTPRE	1.19E-05	0.151	-	-
ENSP00000312122	SEC13	3.31E-05	0.152	-	-
ENSP00000259030	RTP4	2.77E-05	0.152	-	-
ENSP00000349588	ANK2	1.60E-05	0.152	-	-
ENSP00000400010	PTPRJ	1.16E-05	0.152	-	-
ENSP00000262262	CD33	1.11E-05	0.152	-	-
ENSP00000342136	CREB3	1.03E-05	0.152	-	-
ENSP00000299402	APBB1	2.91E-05	0.153	-	-
ENSP00000417899	MECOM	2.06E-05	0.153	-	-
ENSP00000359042	PRPF38B	4.70E-05	0.154	-	-
ENSP00000315955	FOXA2	3.63E-05	0.154	-	-
ENSP00000259881	PSORS1C1	2.28E-05	0.154	-	-
ENSP00000238633	NPC2	2.06E-05	0.154	-	-
ENSP00000261007	CHRNA1	1.46E-05	0.154	-	-
ENSP00000377192	G6PD	3.45E-05	0.155	-	-
ENSP00000243924	PI3	2.74E-05	0.155	-	-
ENSP00000361382	SFTPA1	1.95E-05	0.155	-	-
ENSP00000167218	PDCD2	1.74E-05	0.155	-	-
ENSP00000249364	CALU	1.63E-05	0.155	-	-

ENSP00000301887	BATF2	1.14E-05	0.155	-	-
ENSP00000227378	HSPA8	4.40E-05	0.156	-	-
ENSP00000350928	GAD1	4.32E-05	0.156	-	-
ENSP00000294016	ADCY9	3.72E-05	0.156	-	-
ENSP00000354130	SOX10	3.13E-05	0.156	-	-
ENSP00000272163	LBR	2.57E-05	0.156	-	-
ENSP00000306490	CHRM1	2.32E-05	0.156	-	-
ENSP00000276431	TNFRSF10B	1.87E-05	0.156	-	-
ENSP00000245407	SLC22A5	1.41E-05	0.156	-	-
ENSP00000288943	DUSP2	1.33E-05	0.156	-	-
ENSP00000241014	MAPK8IP1	1.27E-05	0.156	-	-
ENSP00000275198	TAAR6	1.19E-05	0.156	-	-
ENSP00000318585	BACE1	4.78E-05	0.157	-	-
ENSP00000337797	PIDD	2.37E-05	0.157	-	-
ENSP00000398677	TRIM31	2.01E-05	0.157	-	-
ENSP00000245457	PTGER2	1.94E-05	0.157	-	-
ENSP00000367177	PCDH8	1.29E-05	0.157	-	-
ENSP00000180166	FGF20	1.14E-05	0.157	-	-
ENSP00000287934	FZD1	2.49E-05	0.158	-	-
ENSP00000053867	GRN	2.37E-05	0.158	-	-
ENSP00000264009	HSF4	1.83E-05	0.158	-	-
ENSP00000306106	JAGN1	1.12E-05	0.158	-	-

ENSP00000334564	POLR3C	3.32E-05	0.159	-	-
ENSP00000221130	GSR	3.06E-05	0.16	-	-
ENSP00000353512	PARP9	2.80E-05	0.16	-	-
ENSP00000391311	5-Sep	2.50E-05	0.16	-	-
ENSP00000248444	VIL1	2.26E-05	0.16	-	-
ENSP00000356186	LAX1	1.59E-05	0.16	-	-
ENSP00000301202	LAIR2	1.33E-05	0.16	-	-
ENSP00000410649	MAS1L	1.08E-05	0.16	-	-
ENSP00000357307	SYT11	1.91E-05	0.161	-	-
ENSP00000331504	FES	1.62E-05	0.161	-	-
ENSP00000356058	FAIM3	1.24E-05	0.161	-	-
ENSP00000326500	IL11RA	1.06E-05	0.161	-	-
ENSP00000364943	DDAH2	1.03E-05	0.161	-	-
ENSP00000269305	TP53	1.86E-04	0.162	-	-
ENSP00000257290	PDGFRA	3.31E-05	0.163	-	-
ENSP00000256759	FST	3.22E-05	0.163	-	-
ENSP00000357392	EFNA1	1.80E-05	0.163	-	-
ENSP00000233997	AZU1	1.67E-05	0.163	-	-
ENSP00000370867	TGM3	1.56E-05	0.163	-	-
ENSP00000366248	CORT	1.46E-05	0.163	-	-
ENSP00000369317	KRT6A	1.33E-05	0.163	-	-
ENSP00000362777	ATOH7	1.29E-05	0.163	-	-

ENSP00000331766	CLECL1	1.19E-05	0.163	-	-
ENSP00000317271	MCFD2	1.12E-05	0.163	-	-
ENSP00000302413	TPST1	1.00E-05	0.163	-	-
ENSP00000363827	HSPG2	4.51E-05	0.164	-	-
ENSP00000359424	CHUK	3.89E-05	0.164	-	-
ENSP00000278175	ADM	2.54E-05	0.164	-	-
ENSP00000262435	SMURF2	2.14E-05	0.164	-	-
ENSP00000295256	HPGDS	1.82E-05	0.164	-	-
ENSP00000264072	FCER2	1.48E-05	0.164	-	-
ENSP00000261537	MIB1	1.26E-05	0.164	-	-
ENSP00000363826	FZD8	2.29E-05	0.165	-	-
ENSP00000405041	POU5F1	5.14E-05	0.166	-	-
ENSP00000345083	MAP2K3	2.49E-05	0.166	-	-
ENSP00000371372	ATP12A	2.02E-05	0.166	-	-
ENSP00000340983	LRRC25	1.88E-05	0.166	-	-
ENSP00000352565	HAND2	1.65E-05	0.166	-	-
ENSP00000260264	POU2F3	1.27E-05	0.166	-	-
ENSP00000322804	MTSS1	1.01E-05	0.166	-	-
ENSP00000353344	ETS2	2.08E-05	0.167	-	-
ENSP00000229030	FZD10	1.98E-05	0.167	-	-
ENSP00000259726	CDSN	1.97E-05	0.167	-	-
ENSP00000329213	SIVA1	1.13E-05	0.167	-	-

ENSP00000262445	MAP2K4	3.40E-05	0.168	-	-
ENSP00000261023	ITGAV	3.16E-05	0.168	-	-
ENSP00000365131	BAG6	2.31E-05	0.168	-	-
ENSP00000309771	P2RY6	2.15E-05	0.168	-	-
ENSP00000262865	BPI	1.38E-05	0.168	-	-
ENSP00000270223	DMWD	1.29E-05	0.168	-	-
ENSP00000291971	NLRP8	1.18E-05	0.168	-	-
ENSP00000305714	BMP1	2.49E-05	0.169	-	-
ENSP00000363360	INIP	1.85E-05	0.169	-	-
ENSP00000377401	SLC35B2	1.26E-05	0.169	-	-
ENSP00000274532	TIMD4	1.01E-05	0.169	-	-
ENSP00000362092	RRAGC	1.00E-05	0.169	-	-
ENSP00000391685	TRIM31	2.02E-05	0.17	-	-
ENSP00000327214	ATP13A2	1.98E-05	0.17	-	-
ENSP00000279488	DUSP6	1.69E-05	0.171	-	-
ENSP00000387768	HLA-DQA2	2.05E-05	0.172	-	-
ENSP00000276571	CRH	3.58E-05	0.173	-	-
ENSP00000259915	POU5F1	2.73E-05	0.173	-	-
ENSP00000311469	GSTM1	2.58E-05	0.173	-	-
ENSP00000361311	TMEM53	1.84E-05	0.173	-	-
ENSP00000319610	NPAS3	1.52E-05	0.173	-	-
ENSP00000339813	TPST2	1.03E-05	0.173	-	-

ENSP00000348635	AMICA1	3.16E-05	0.174	-	-
ENSP00000278282	SCGB1A1	2.42E-05	0.174	-	-
ENSP00000266041	ITIH4	1.82E-05	0.174	-	-
ENSP00000411397	MARK3	1.61E-05	0.174	-	-
ENSP00000406229	FAT1	1.54E-05	0.174	-	-
ENSP00000245912	TNFSF14	1.37E-05	0.174	-	-
ENSP00000269439	RNF165	1.21E-05	0.174	-	-
ENSP00000264634	WNT5A	2.16E-05	0.175	-	-
ENSP00000312664	CASP2	1.58E-05	0.175	-	-
ENSP00000412283	IER3	1.52E-05	0.175	-	-
ENSP00000347184	HTT	3.37E-05	0.176	-	-
ENSP00000314774	HES7	1.91E-05	0.176	-	-
ENSP00000313084	EGFLAM	1.74E-05	0.176	-	-
ENSP00000260723	BTBD16	1.54E-05	0.176	-	-
ENSP00000349428	PTBP1	2.72E-05	0.177	-	-
ENSP00000252242	KRT5	2.41E-05	0.177	-	-
ENSP00000301420	KLK1	1.63E-05	0.177	-	-
ENSP00000303575	NUDT9	1.32E-05	0.177	-	-
ENSP00000262866	SLA2	1.14E-05	0.177	-	-
ENSP00000318822	BID	1.12E-05	0.177	-	-
ENSP00000301411	NTF4	1.04E-05	0.177	-	-
ENSP00000219255	PARD6A	2.62E-05	0.178	-	-

ENSP00000268097	HEXA	2.33E-05	0.178	-	-
ENSP00000366593	TMEM201	1.59E-05	0.178	-	-
ENSP00000349041	NRARP	1.29E-05	0.178	-	-
ENSP00000271915	KCNN3	1.04E-05	0.178	-	-
ENSP00000417300	TRBC2	4.48E-05	0.179	-	-
ENSP00000374985	IGHG4	1.75E-05	0.179	-	-
ENSP00000362014	DNM1	2.42E-05	0.18	-	-
ENSP00000261822	BCL7A	1.25E-05	0.18	-	-
ENSP00000363708	BMPR2	3.11E-05	0.181	-	-
ENSP00000283147	BMP6	1.97E-05	0.181	-	-
ENSP00000314480	UNCX	1.29E-05	0.181	-	-
ENSP00000248996	GNAZ	2.30E-05	0.182	-	-
ENSP00000277508	PAEP	1.01E-05	0.182	-	-
ENSP00000394134	C6orf100	1.85E-05	0.183	-	-
ENSP00000396675	C6orf100	1.85E-05	0.183	-	-
ENSP00000306662	ADRA1B	1.77E-05	0.183	-	-
ENSP00000262160	SMAD2	6.45E-05	0.184	-	-
ENSP00000301633	BIRC5	5.02E-05	0.184	-	-
ENSP00000345206	RBPJ	4.08E-05	0.184	-	-
ENSP00000415793	MUC5B	3.89E-05	0.184	-	-
ENSP00000352708	TRAPPC2	2.49E-05	0.184	-	-
ENSP00000306279	UMOD	2.21E-05	0.184	-	-

ENSP00000394700	SYT16	1.93E-05	0.184	-	-
ENSP00000337340	MED19	1.60E-05	0.184	-	-
ENSP00000357348	HEY2	1.33E-05	0.184	-	-
ENSP00000354158	ERC1	1.96E-05	0.185	-	-
ENSP00000342828	NKX2-3	1.79E-05	0.185	-	-
ENSP00000381333	CRHR1	1.63E-05	0.185	-	-
ENSP00000344223	MBTPS1	1.04E-05	0.185	-	-
ENSP00000332973	SMAD3	5.51E-05	0.186	-	-
ENSP00000321106	TAC1	4.56E-05	0.186	-	-
ENSP00000360687	PTGDS	2.31E-05	0.186	-	-
ENSP00000244930	SCGB2A1	1.26E-05	0.186	-	-
ENSP00000341342	KLHL6	1.09E-05	0.186	-	-
ENSP00000219548	STUB1	3.47E-05	0.187	-	-
ENSP00000367570	PHF11	2.07E-05	0.187	-	-
ENSP00000264658	FBXL20	1.55E-05	0.187	-	-
ENSP00000355010	IGSF1	1.28E-05	0.187	-	-
ENSP00000358596	DUSP5	1.14E-05	0.187	-	-
ENSP00000308219	RGMB	1.14E-05	0.187	-	-
ENSP00000263100	A1BG	1.08E-05	0.187	-	-
ENSP00000382362	PHB2	4.81E-05	0.188	-	-
ENSP00000404623	DAXX	1.99E-05	0.188	-	-
ENSP00000300456	SLC27A4	1.94E-05	0.188	-	-

ENSP00000250066	USP6	1.33E-05	0.188	-	-
ENSP00000353508	MAP2	2.57E-05	0.189	-	-
ENSP00000381648	PIAS2	1.91E-05	0.189	-	-
ENSP00000355629	COG2	1.57E-05	0.189	-	-
ENSP00000085068	ISOC2	1.48E-05	0.189	-	-
ENSP00000280154	PDCD4	1.38E-05	0.189	-	-
ENSP00000347836	UBE2H	3.74E-05	0.19	-	-
ENSP00000401317	TUBB	2.00E-05	0.19	-	-
ENSP00000164024	CELSR3	1.65E-05	0.19	-	-
ENSP00000387091	ZNF365	1.45E-05	0.19	-	-
ENSP00000362555	RNF19B	1.38E-05	0.19	-	-
ENSP00000319141	CYBRD1	1.19E-05	0.19	-	-
ENSP00000274026	CCNA2	4.19E-05	0.191	-	-
ENSP00000363787	RING1	2.20E-05	0.191	-	-
ENSP00000377380	ARHGAP9	2.10E-05	0.191	-	-
ENSP00000306461	SPRR1B	1.98E-05	0.191	-	-
ENSP00000339004	FOXG1	1.73E-05	0.191	-	-
ENSP00000375622	LAIR1	1.27E-05	0.191	-	-
ENSP00000329554	HIST1H3I	1.26E-05	0.191	-	-
ENSP00000265175	SEC24B	3.51E-05	0.192	-	-
ENSP00000248923	GGT1	3.48E-05	0.192	-	-
ENSP00000363397	UGCG	2.49E-05	0.192	-	-

ENSP00000264674	MECOM	2.05E-05	0.192	-	-
ENSP00000327824	IFNLR1	1.47E-05	0.192	-	-
ENSP00000331746	CALCA	3.89E-05	0.193	-	-
ENSP00000359539	GOT1	3.21E-05	0.193	-	-
ENSP00000357643	MKI67	2.63E-05	0.193	-	-
ENSP00000292896	HBE1	2.25E-05	0.193	-	-
ENSP00000222266	PSENEN	1.83E-05	0.193	-	-
ENSP00000374989	IGHA1	2.37E-05	0.194	-	-
ENSP00000410635	C6orf100	1.87E-05	0.194	-	-
ENSP00000302486	MAP2K1	3.14E-05	0.195	-	-
ENSP00000361878	CAP1	2.73E-05	0.195	-	-
ENSP00000353073	CLN3	2.13E-05	0.195	-	-
ENSP00000375009	IGHV3-9	2.09E-05	0.195	-	-
ENSP00000365747	PQBP1	1.59E-05	0.195	-	-
ENSP00000358423	RRAGD	1.00E-05	0.195	-	-
ENSP00000319377	NLRP12	1.89E-05	0.196	-	-
ENSP00000287437	NSMCE2	1.26E-05	0.196	-	-
ENSP00000369981	SH3GL2	2.78E-05	0.197	-	-
ENSP00000295108	NEUROD1	2.49E-05	0.197	-	-
ENSP00000339001	TUBB	1.98E-05	0.197	-	-
ENSP00000261769	CDH1	8.50E-05	0.198	-	-
ENSP00000300161	YWHAB	4.77E-05	0.198	-	-

ENSP00000360798	EPS15	4.05E-05	0.198	-	-
ENSP00000222381	PON1	2.37E-05	0.198	-	-
ENSP00000219334	SMPD3	2.14E-05	0.198	-	-
ENSP00000379612	TAX1BP1	1.60E-05	0.198	-	-
ENSP00000311733	CMKLR1	1.56E-05	0.198	-	-
ENSP00000360968	CYP4X1	3.86E-05	0.199	-	-
ENSP00000370376	DUT	2.64E-05	0.199	-	-
ENSP00000296677	F2RL1	2.43E-05	0.199	-	-
ENSP00000359019	PITX3	1.89E-05	0.199	-	-
ENSP00000258787	MYO1G	1.05E-05	0.199	-	-
ENSP00000262510	NLRC5	1.00E-05	0.199	-	-
ENSP00000294304	LRP5	2.79E-05	0.2	-	-
ENSP00000222573	ITGB8	2.02E-05	0.2	-	-
ENSP00000349359	SASH3	1.94E-05	0.2	-	-
ENSP00000356789	ATP1B1	1.22E-05	0.2	-	-
ENSP00000209540	OR1I1	2.05E-05	0.201	-	-
ENSP00000350914	BDH1	1.53E-05	0.201	-	-
ENSP00000221132	TNFRSF10A	1.28E-05	0.201	-	-
ENSP00000379204	BMP7	2.93E-05	0.202	-	-
ENSP00000361845	ZMPSTE24	1.91E-05	0.202	-	-
ENSP00000365514	ZIC2	1.51E-05	0.202	-	-
ENSP00000339399	CRYZ	3.08E-05	0.203	-	-

ENSP00000371393	PGM2	1.65E-05	0.203	-	-
ENSP00000374981	IGHA2	1.61E-05	0.203	-	-
ENSP00000377344	DDX60	1.61E-05	0.203	-	-
ENSP00000308733	GPR151	1.14E-05	0.203	-	-
ENSP00000338207	LMO1	1.66E-05	0.204	-	-
ENSP00000310219	HSPA6	1.34E-05	0.204	-	-
ENSP00000336927	ALDOA	2.66E-05	0.205	-	-
ENSP00000362948	MED18	1.77E-05	0.205	-	-
ENSP00000222304	HAMP	1.68E-05	0.205	-	-
ENSP00000364709	F10	2.95E-05	0.206	-	-
ENSP00000307445	LTB4R	1.78E-05	0.206	-	-
ENSP00000370256	FOXC1	1.37E-05	0.206	-	-
ENSP00000295206	EN1	2.01E-05	0.207	-	-
ENSP00000257963	ACVR1B	1.73E-05	0.207	-	-
ENSP00000298556	HPRT1	2.65E-05	0.208	-	-
ENSP00000376848	PRIMA1	1.17E-05	0.208	-	-
ENSP00000254801	IGJ	1.03E-05	0.208	-	-
ENSP00000307046	SDC2	4.15E-05	0.209	-	-
ENSP00000340409	SMPD1	2.23E-05	0.209	-	-
ENSP00000302251	GBX2	1.51E-05	0.209	-	-
ENSP00000363390	TRIM63	1.40E-05	0.209	-	-
ENSP00000377112	SLC12A8	1.08E-05	0.209	-	-

ENSP00000374777	IGKC	4.29E-05	0.21	-	-
ENSP00000262623	ATP4A	1.90E-05	0.21	-	-
ENSP00000342235	ERBB4	4.29E-05	0.211	-	-
ENSP00000302216	ATOH1	1.67E-05	0.211	-	-
ENSP00000322542	GTF2I	1.93E-05	0.212	-	-
ENSP00000304467	THOP1	1.89E-05	0.212	-	-
ENSP00000264668	MTRR	1.18E-05	0.212	-	-
ENSP00000265192	PAIP2	1.01E-05	0.212	-	-
ENSP00000225577	RPS6KB1	3.19E-05	0.213	-	-
ENSP00000360025	GADD45A	2.70E-05	0.213	-	-
ENSP00000410715	SFRP4	2.58E-05	0.213	-	-
ENSP00000355751	THBS2	2.24E-05	0.213	-	-
ENSP00000301607	EVPL	1.34E-05	0.213	-	-
ENSP00000322276	VNN2	1.27E-05	0.213	-	-
ENSP00000301761	SDHAF2	1.22E-05	0.213	-	-
ENSP00000256999	FOLH1	1.18E-05	0.213	-	-
ENSP00000257189	DSG3	1.13E-05	0.213	-	-
ENSP00000318057	EGR3	1.58E-05	0.214	-	-
ENSP00000377372	GAP43	1.58E-05	0.214	-	-
ENSP00000254722	SERPINF1	1.54E-05	0.214	-	-
ENSP00000392328	ZBTB12	1.11E-05	0.214	-	-
ENSP00000246891	CSN1S1	7.62E-05	0.215	-	-

ENSP00000391681	POU5F1	3.13E-05	0.215	-	-
ENSP00000256935	DOCK2	1.42E-05	0.215	-	-
ENSP00000353582	NRP2	1.38E-05	0.215	-	-
ENSP00000414777	ZBTB12	1.11E-05	0.215	-	-
ENSP00000005340	DVL2	3.67E-05	0.216	-	-
ENSP00000249373	SMO	2.47E-05	0.216	-	-
ENSP00000338272	HEY1	1.69E-05	0.216	-	-
ENSP00000219599	CRYM	1.56E-05	0.216	-	-
ENSP00000339906	PAX4	1.48E-05	0.216	-	-
ENSP00000292599	MAML1	1.06E-05	0.216	-	-
ENSP00000346032	ANXA2	3.00E-05	0.217	-	-
ENSP00000219150	CORO1A	2.76E-05	0.217	-	-
ENSP00000413720	CDKN1C	2.42E-05	0.217	-	-
ENSP00000357040	VANGL2	2.09E-05	0.217	-	-
ENSP00000306099	FGB	1.81E-05	0.217	-	-
ENSP00000305924	PPP4R4	1.26E-05	0.217	-	-
ENSP00000228606	CYP27B1	1.23E-05	0.217	-	-
ENSP00000258774	HUS1	1.10E-05	0.217	-	-
ENSP00000383746	FCAMR	1.01E-05	0.217	-	-
ENSP00000402060	PVR	1.31E-05	0.218	-	-
ENSP00000254235	ADCY7	3.06E-05	0.219	-	-
ENSP00000261798	CSNK1A1	2.88E-05	0.219	-	-

ENSP00000265333	VDAC1	2.22E-05	0.219	-	-
ENSP00000368755	MUC12	2.22E-05	0.219	-	-
ENSP00000338157	ZBTB16	2.10E-05	0.22	-	-
ENSP00000220509	VPS18	1.59E-05	0.22	-	-
ENSP00000259470	CTSL2	1.34E-05	0.22	-	-
ENSP00000242261	TWIST1	1.32E-05	0.22	-	-
ENSP00000262493	GNAO1	2.86E-05	0.221	-	-
ENSP00000343313	ATG5	2.15E-05	0.221	-	-
ENSP00000350425	APOA4	1.30E-05	0.221	-	-
ENSP00000299022	LIPC	2.67E-05	0.222	-	-
ENSP00000362924	GSN	2.62E-05	0.222	-	-
ENSP00000337014	HFE2	1.61E-05	0.222	-	-
ENSP00000360689	TNKS2	1.18E-05	0.222	-	-
ENSP00000201586	SULT2B1	4.03E-05	0.223	-	-
ENSP00000358617	PHTF1	2.51E-05	0.223	-	-
ENSP00000358674	UBL4A	1.67E-05	0.223	-	-
ENSP00000303252	ZNF804A	1.35E-05	0.223	-	-
ENSP00000326119	PMAIP1	1.25E-05	0.223	-	-
ENSP00000401435	VPS53	1.44E-05	0.224	-	-
ENSP00000360519	RBP4	1.32E-05	0.224	-	-
ENSP00000360247	CYP2J2	3.79E-05	0.225	-	-
ENSP00000168712	FGF4	2.78E-05	0.225	-	-

ENSP00000316338	BAIAP2	1.78E-05	0.225	-	-
ENSP00000363071	DES	3.91E-05	0.226	-	-
ENSP00000310305	P2RY2	2.05E-05	0.226	-	-
ENSP00000257818	LMO2	2.04E-05	0.226	-	-
ENSP00000362717	LHX2	1.90E-05	0.226	-	-
ENSP00000358105	APH1A	1.26E-05	0.226	-	-
ENSP00000295728	CRYBA2	1.15E-05	0.226	-	-
ENSP00000321334	LPA	2.21E-05	0.227	-	-
ENSP00000167586	KRT14	1.78E-05	0.228	-	-
ENSP00000284292	NRGN	1.24E-05	0.228	-	-
ENSP00000374558	KIAA0226L	1.21E-05	0.228	-	-
ENSP00000335657	CCK	3.07E-05	0.229	-	-
ENSP00000328181	NOG	3.06E-05	0.229	-	-
ENSP00000404817	TRGC1	2.10E-05	0.229	-	-
ENSP00000323967	SMARCE1	2.08E-05	0.229	-	-
ENSP00000257555	HNF1A	1.73E-05	0.229	-	-
ENSP00000222598	DLX5	1.69E-05	0.229	-	-
ENSP00000295367	SPRR3	1.69E-05	0.229	-	-
ENSP00000256495	BHLHE40	1.63E-05	0.229	-	-
ENSP00000355785	LEFTY2	1.39E-05	0.229	-	-
ENSP00000312702	DEFB114	1.06E-05	0.229	-	-
ENSP00000363489	GDF5	1.64E-05	0.23	-	-

ENSP00000366395	SYVN1	2.37E-05	0.231	-	-
ENSP00000221566	SGTA	1.85E-05	0.231	-	-
ENSP00000181796	FAM107B	1.41E-05	0.231	-	-
ENSP00000222792	CHN2	1.14E-05	0.231	-	-
ENSP00000309503	YWHAZ	5.83E-05	0.232	-	-
ENSP00000308928	CYLD	3.44E-05	0.232	-	-
ENSP00000267082	ITGB7	2.96E-05	0.232	-	-
ENSP00000303522	TACR1	1.90E-05	0.232	-	-
ENSP00000365280	ID1	1.65E-05	0.232	-	-
ENSP00000264345	HERC3	1.14E-05	0.232	-	-
ENSP00000333837	MRPL12	2.87E-05	0.233	-	-
ENSP00000245932	VASP	2.78E-05	0.233	-	-
ENSP00000296084	RYK	1.84E-05	0.233	-	-
ENSP00000270142	SOD1	5.43E-05	0.234	-	-
ENSP00000291525	TFF3	1.30E-05	0.234	-	-
ENSP00000320548	DNAJC4	1.16E-05	0.234	-	-
ENSP00000350359	CREB5	1.04E-05	0.234	-	-
ENSP00000228307	PXN	3.68E-05	0.235	-	-
ENSP00000005257	RALA	3.46E-05	0.235	-	-
ENSP00000315011	EDNRA	3.28E-05	0.235	-	-
ENSP00000211287	MAPK13	3.39E-05	0.236	-	-
ENSP00000332816	PTK2B	2.58E-05	0.236	-	-

ENSP00000276925	CDKN2B	1.77E-05	0.236	-	-
ENSP00000282111	TCF7L1	1.57E-05	0.236	-	-
ENSP00000297316	SOX17	1.53E-05	0.236	-	-
ENSP00000252593	BST2	1.47E-05	0.236	-	-
ENSP00000328858	PYCR1	1.14E-05	0.236	-	-
ENSP00000263867	CAPG	1.07E-05	0.236	-	-
ENSP00000265165	LEF1	3.14E-05	0.237	-	-
ENSP00000300055	PLIN1	2.44E-05	0.237	-	-
ENSP00000368924	TFAP2A	1.76E-05	0.237	-	-
ENSP00000000442	ESRRA	1.68E-05	0.237	-	-
ENSP00000297689	NFIL3	1.44E-05	0.237	-	-
ENSP00000354718	AKAP13	1.38E-05	0.237	-	-
ENSP00000354458	C8A	1.34E-05	0.237	-	-
ENSP00000284984	ADAMTS1	1.24E-05	0.237	-	-
ENSP00000262367	CREBBP	7.84E-05	0.238	-	-
ENSP00000398736	CYR61	1.52E-05	0.238	-	-
ENSP00000217131	CTSZ	1.10E-05	0.238	-	-
ENSP00000374455	SQSTM1	3.14E-05	0.239	-	-
ENSP00000305422	CEBPB	2.91E-05	0.239	-	-
ENSP00000289575	SLCO2B1	2.07E-05	0.239	-	-
ENSP00000234739	BCL9	1.25E-05	0.239	-	-
ENSP00000302625	AXIN2	2.66E-05	0.24	-	-

ENSP00000295685	ARPC2	2.15E-05	0.24	-	-
ENSP00000263640	ACVR1	1.90E-05	0.24	-	-
ENSP00000364902	POFUT1	1.60E-05	0.24	-	-
ENSP00000354774	SPRYD7	1.05E-05	0.24	-	-
ENSP00000267978	MAN2C1	1.24E-05	0.241	-	-
ENSP00000312126	ADCY4	4.13E-05	0.242	-	-
ENSP00000223366	GCK	2.08E-05	0.242	-	-
ENSP00000263552	TBXAS1	1.67E-05	0.242	-	-
ENSP00000273320	ZKSCAN7	2.41E-05	0.243	-	-
ENSP00000331871	RHD	1.29E-05	0.243	-	-
ENSP00000304169	PITX2	2.01E-05	0.244	-	-
ENSP00000359783	IFI44	1.75E-05	0.244	-	-
ENSP00000310785	MRPS22	1.10E-05	0.244	-	-
ENSP00000356975	ADAMTS4	1.05E-05	0.244	-	-
ENSP00000252809	GDF15	1.42E-05	0.245	-	-
ENSP00000307080	EGLN2	1.36E-05	0.245	-	-
ENSP00000378394	PSAP	1.49E-05	0.246	-	-
ENSP00000371554	SOD3	1.31E-05	0.246	-	-
ENSP00000252519	ACE2	1.24E-05	0.246	-	-
ENSP00000296849	NKD2	1.17E-05	0.246	-	-
ENSP00000303211	ACHE	2.00E-05	0.247	-	-
ENSP00000295619	PROK2	1.72E-05	0.247	-	-

ENSP00000352252	HIST1H3J	1.30E-05	0.247	-	-
ENSP00000327650	VPS33B	1.05E-05	0.247	-	-
ENSP00000344193	RNASE1	3.68E-05	0.248	-	-
ENSP00000228938	MGP	4.08E-05	0.249	-	-
ENSP00000329127	PRKCH	1.78E-05	0.249	-	-
ENSP00000408012	CCHCR1	1.45E-05	0.249	-	-
ENSP00000294702	GFI1	1.29E-05	0.249	-	-
ENSP00000261817	PSMD9	1.23E-05	0.249	-	-
ENSP00000311579	TNKS	1.10E-05	0.249	-	-
ENSP00000355759	PARP1	5.40E-05	0.25	-	-
ENSP00000265986	IDE	3.88E-05	0.25	-	-
ENSP00000318916	MARCO	1.57E-05	0.25	-	-
ENSP00000263277	EHD2	1.13E-05	0.25	-	-
ENSP00000274813	MUT	2.97E-05	0.251	-	-
ENSP00000304207	MUC4	2.54E-05	0.251	-	-
ENSP00000231121	HAND1	1.04E-05	0.251	-	-
ENSP00000257192	DSG1	1.55E-05	0.252	-	-
ENSP00000309474	PSMD1	2.25E-05	0.253	-	-
ENSP00000252891	NUMBL	1.18E-05	0.253	-	-
ENSP00000374856	IGLC2	1.11E-05	0.253	-	-
ENSP00000246062	MKKS	2.10E-05	0.254	-	-
ENSP00000243077	LRP1	1.20E-05	0.254	-	-

ENSP00000271331	PROK1	1.09E-05	0.254	-	-
ENSP00000366267	GPC5	2.60E-05	0.255	-	-
ENSP00000206765	TGM1	1.86E-05	0.255	-	-
ENSP00000216037	XBP1	1.83E-05	0.255	-	-
ENSP00000178640	MAP2K5	1.26E-05	0.255	-	-
ENSP00000240328	TBX2	1.13E-05	0.255	-	-
ENSP00000380280	FGFR1	3.55E-05	0.256	-	-
ENSP00000281708	FBXW7	2.83E-05	0.256	-	-
ENSP00000268164	ST8SIA2	1.04E-05	0.256	-	-
ENSP00000200676	CETP	1.15E-05	0.257	-	-
ENSP00000410294	FGFR2	3.55E-05	0.258	-	-
ENSP00000359563	CDR1	2.61E-05	0.258	-	-
ENSP00000259938	CLPS	1.29E-05	0.258	-	-
ENSP00000270517	ECSIT	1.17E-05	0.258	-	-
ENSP00000386212	PSMB11	1.12E-05	0.258	-	-
ENSP00000345681	GATA2	2.93E-05	0.259	-	-
ENSP00000396774	MUC20	2.22E-05	0.259	-	-
ENSP00000324834	MUC3A	2.17E-05	0.259	-	-
ENSP00000312029	UCP2	1.54E-05	0.259	-	-
ENSP00000357905	DMBT1	1.22E-05	0.259	-	-
ENSP00000245206	GOT2	4.29E-05	0.26	-	-
ENSP00000347495	WARS	3.20E-05	0.261	-	-

ENSP00000339845	DROSHA	2.16E-05	0.261	-	-
ENSP00000410071	TUBB	1.96E-05	0.261	-	-
ENSP00000310771	GRB7	1.49E-05	0.261	-	-
ENSP00000351789	PELI1	1.15E-05	0.261	-	-
ENSP00000344166	TMEM189-UBE2V1	1.14E-05	0.261	-	-
ENSP00000337697	DCX	2.30E-05	0.262	-	-
ENSP00000341625	CYB5A	1.67E-05	0.262	-	-
ENSP00000317619	LACC1	1.23E-05	0.262	-	-
ENSP00000372126	DEFA1	1.06E-05	0.262	-	-
ENSP00000321810	TRIT1	1.76E-05	0.263	-	-
ENSP00000352425	WASF1	1.20E-05	0.264	-	-
ENSP00000237014	TTR	3.12E-05	0.265	-	-
ENSP00000252505	ALLC	1.63E-05	0.265	-	-
ENSP00000335074	GHRL	1.50E-05	0.265	-	-
ENSP00000294413	RHCE	1.22E-05	0.265	-	-
ENSP00000415151	CBFB	1.05E-05	0.265	-	-
ENSP00000364094	ITGB1	4.05E-05	0.266	-	-
ENSP00000325120	PGR	3.52E-05	0.266	-	-
ENSP00000375018	IGHV3-23	2.83E-05	0.266	-	-
ENSP00000335311	EDNRB	2.31E-05	0.266	-	-
ENSP00000225893	HNF1B	2.08E-05	0.266	-	-
ENSP00000289779	F11R	1.75E-05	0.266	-	-

ENSP00000266991	DHH	1.30E-05	0.266	-	-
ENSP00000210313	PSMD5	1.20E-05	0.266	-	-
ENSP00000355124	KRT19	1.52E-05	0.267	-	-
ENSP00000338461	CYB5R3	1.09E-05	0.267	-	-
ENSP00000262995	GAB1	1.03E-05	0.267	-	-
ENSP00000263253	EP300	7.13E-05	0.268	-	-
ENSP00000310244	RASGRP1	1.66E-05	0.268	-	-
ENSP00000318089	LPP	1.28E-05	0.268	-	-
ENSP00000411096	ABCA13	1.23E-05	0.268	-	-
ENSP00000011619	RANBP9	1.17E-05	0.268	-	-
ENSP00000007969	LRRC23	1.47E-05	0.269	-	-
ENSP00000332454	RIPK4	1.40E-05	0.269	-	-
ENSP00000353581	HIST1H3E	1.29E-05	0.269	-	-
ENSP00000344413	PSG5	1.02E-05	0.269	-	-
ENSP00000419361	ADCY5	3.12E-05	0.27	-	-
ENSP00000335384	ZPBP2	1.02E-05	0.271	-	-
ENSP00000269886	SH3GL1	4.52E-05	0.272	-	-
ENSP00000204961	EFNB1	2.40E-05	0.272	-	-
ENSP00000356992	ARHGAP30	1.70E-05	0.272	-	-
ENSP00000358698	WNT2B	1.45E-05	0.272	-	-
ENSP00000369217	ARIH1	1.14E-05	0.272	-	-
ENSP00000358511	CNR1	2.38E-05	0.273	-	-

ENSP00000252490	APOC4	1.32E-05	0.273	-	-
ENSP00000311405	ADCY6	3.18E-05	0.274	-	-
ENSP00000328777	EFNA5	1.32E-05	0.274	-	-
ENSP00000307006	IRX2	1.03E-05	0.274	-	-
ENSP00000228476	DAO	1.39E-05	0.275	-	-
ENSP00000366999	HIST1H3D	1.19E-05	0.275	-	-
ENSP00000323264	MARVELD2	1.11E-05	0.275	-	-
ENSP00000293373	NCKAP1L	1.90E-05	0.276	-	-
ENSP00000356070	MAPKAPK2	1.38E-05	0.276	-	-
ENSP00000376776	DBH	1.06E-05	0.276	-	-
ENSP00000360372	CYP2C19	3.96E-05	0.277	-	-
ENSP00000286355	ADCY8	3.11E-05	0.277	-	-
ENSP00000324270	OXTR	2.54E-05	0.277	-	-
ENSP00000259632	DCTN3	2.02E-05	0.277	-	-
ENSP00000373347	SRGAP3	1.55E-05	0.277	-	-
ENSP00000299847	CHRFAM7A	1.26E-05	0.277	-	-
ENSP00000311095	CYP4A11	1.66E-05	0.278	-	-
ENSP00000380444	MYO9B	1.44E-05	0.278	-	-
ENSP00000349076	ATN1	4.10E-05	0.279	-	-
ENSP00000354586	GLI2	2.56E-05	0.279	-	-
ENSP00000415769	ITIH3	2.31E-05	0.279	-	-
ENSP00000261304	GALC	1.38E-05	0.279	-	-

ENSP00000283249	ITGB6	1.68E-05	0.28	-	-
ENSP00000293308	KRT8	1.59E-05	0.28	-	-
ENSP00000358160	HIST1H3H	1.14E-05	0.28	-	-
ENSP00000364578	NELFE	1.10E-05	0.28	-	-
ENSP00000334490	ZDHHC8	1.48E-05	0.281	-	-
ENSP00000268383	CDR2	2.35E-05	0.283	-	-
ENSP00000354398	CYTH1	1.18E-05	0.283	-	-
ENSP00000219091	ZNF205	1.06E-05	0.283	-	-
ENSP00000337915	CYP3A4	4.91E-05	0.284	-	-
ENSP00000000412	M6PR	1.72E-05	0.284	-	-
ENSP00000307305	DDIT4	1.35E-05	0.284	-	-
ENSP00000340671	DDIT3	1.99E-05	0.285	-	-
ENSP00000300035	KIAA0101	2.53E-05	0.286	-	-
ENSP00000312235	MUC13	2.22E-05	0.286	-	-
ENSP00000258052	SMPD2	1.28E-05	0.286	-	-
ENSP00000372136	DEFA1B	1.08E-05	0.286	-	-
ENSP00000313070	PPIP5K2	1.74E-05	0.287	-	-
ENSP00000418823	FTO	1.43E-05	0.287	-	-
ENSP00000247306	CTAG2	1.31E-05	0.287	-	-
ENSP00000289952	SLC39A14	1.03E-05	0.287	-	-
ENSP00000361423	ABL1	5.13E-05	0.288	-	-
ENSP00000255465	CCNA1	2.96E-05	0.288	-	-

ENSP00000264381	BCHE	1.66E-05	0.288	-	-
ENSP00000383690	MASP2	1.43E-05	0.288	-	-
ENSP00000296946	T	1.41E-05	0.288	-	-
ENSP00000268057	BBS4	1.36E-05	0.288	-	-
ENSP00000306991	HAS2	1.05E-05	0.288	-	-
ENSP00000244007	PLCG1	4.21E-05	0.289	-	-
ENSP00000276079	NONO	2.34E-05	0.289	-	-
ENSP00000361562	CTSA	1.81E-05	0.289	-	-
ENSP00000352852	MDK	1.68E-05	0.289	-	-
ENSP00000311489	SPTBN2	1.62E-05	0.289	-	-
ENSP00000384192	RASGRP3	1.24E-05	0.289	-	-
ENSP00000358866	FLNA	3.10E-05	0.29	-	-
ENSP00000216727	PABPN1	2.04E-05	0.29	-	-
ENSP00000380019	ZNF384	1.70E-05	0.29	-	-
ENSP00000324633	DEFB103B	1.29E-05	0.29	-	-
ENSP00000337816	MMP25	1.03E-05	0.29	-	-
ENSP00000387699	CREB1	5.66E-05	0.291	-	-
ENSP00000340820	MAPT	3.32E-05	0.291	-	-
ENSP00000390018	C6orf10	1.51E-05	0.291	-	-
ENSP00000369055	B4GALT1	1.36E-05	0.291	-	-
ENSP00000224337	BLNK	1.09E-05	0.291	-	-
ENSP00000331514	ACTG1	5.56E-05	0.292	-	-

ENSP00000265715	SLC26A4	1.81E-05	0.292	-	-
ENSP00000253801	G6PC	1.22E-05	0.292	-	-
ENSP00000380523	CAPSL	1.04E-05	0.292	-	-
ENSP00000415183	MUC2	2.85E-05	0.293	-	-
ENSP00000293549	WNT1	2.41E-05	0.293	-	-
ENSP00000315383	ASPRV1	1.16E-05	0.293	-	-
ENSP00000312436	NFE2	1.07E-05	0.293	-	-
ENSP00000304283	RAC3	6.18E-05	0.294	-	-
ENSP00000352721	DNM2	3.34E-05	0.294	-	-
ENSP00000338345	SNCA	2.84E-05	0.294	-	-
ENSP00000253934	TMEM204	2.29E-05	0.294	-	-
ENSP00000385721	GGT2	1.70E-05	0.294	-	-
ENSP00000252456	CNN1	1.05E-05	0.294	-	-
ENSP00000259365	TMOD1	1.00E-05	0.294	-	-
ENSP00000358335	MAP3K7	2.95E-05	0.295	-	-
ENSP00000269299	ASGR1	2.02E-05	0.295	-	-
ENSP00000394863	PPT1	1.52E-05	0.295	-	-
ENSP00000329374	SERPINA7	1.46E-05	0.295	-	-
ENSP00000257934	ESPL1	2.37E-05	0.296	-	-
ENSP00000354486	GOLGA4	1.82E-05	0.296	-	-
ENSP00000331608	IRX3	1.25E-05	0.296	-	-
ENSP00000356094	AVPR1B	1.01E-05	0.296	-	-

ENSP00000387662	GCG	5.94E-05	0.297	-	-
ENSP00000301141	CYP2A6	3.87E-05	0.297	-	-
ENSP00000302111	MGMT	2.07E-05	0.297	-	-
ENSP00000264568	BMPR1B	2.03E-05	0.297	-	-
ENSP00000360916	VAV2	2.58E-05	0.298	-	-
ENSP00000397297	NTF3	1.47E-05	0.298	-	-
ENSP00000252244	KRT1	1.39E-05	0.298	-	-
ENSP00000225174	PPIF	1.09E-05	0.298	-	-
ENSP00000364270	XPA	2.06E-05	0.299	-	-
ENSP00000278968	TAGLN	1.84E-05	0.299	-	-
ENSP00000215727	SERPIND1	1.82E-05	0.299	-	-
ENSP00000318982	EPSTI1	1.46E-05	0.299	-	-
ENSP00000355361	CD47	1.38E-05	0.299	-	-
ENSP00000290039	CACHD1	1.21E-05	0.299	-	-
ENSP00000369843	EPHX2	2.20E-05	0.3	-	-
ENSP00000231749	ZMYND10	1.62E-05	0.3	-	-
ENSP00000305824	KCNS3	1.28E-05	0.3	-	-
ENSP00000295408	MERTK	1.19E-05	0.3	-	-
ENSP00000228510	MVK	1.17E-05	0.3	-	-
ENSP00000369647	AVP	2.15E-05	0.301	-	-
ENSP00000342087	FHIT	1.87E-05	0.301	-	-
ENSP00000401397	DDR1	1.13E-05	0.301	-	-

ENSP00000251849	RAF1	2.51E-05	0.303	-	-
ENSP00000233242	APOB	4.29E-05	0.305	-	-
ENSP00000339467	RHOG	2.60E-05	0.305	-	-
ENSP00000386896	ITGA6	1.92E-05	0.305	-	-
ENSP00000350199	AP1B1	1.43E-05	0.305	-	-
ENSP00000331358	GAST	2.45E-05	0.306	-	-
ENSP00000347169	NUMB	2.37E-05	0.306	-	-
ENSP00000303424	PTGDR	1.49E-05	0.306	-	-
ENSP00000329357	SP1	4.58E-05	0.307	-	-
ENSP00000315757	LCP1	2.12E-05	0.307	-	-
ENSP00000282928	ZIC1	1.24E-05	0.307	-	-
ENSP00000296777	CARTPT	1.22E-05	0.307	-	-
ENSP00000328928	HOXB4	1.12E-05	0.307	-	-
ENSP00000333769	BSG	2.02E-05	0.308	-	-
ENSP00000327758	NKX2-5	1.83E-05	0.308	-	-
ENSP00000352257	XRCC6	2.91E-05	0.309	-	-
ENSP00000356436	PLA2G4A	2.30E-05	0.309	-	-
ENSP00000357130	SPTA1	2.25E-05	0.309	-	-
ENSP00000249396	SIRT2	2.11E-05	0.309	-	-
ENSP00000215794	USP18	1.45E-05	0.309	-	-
ENSP00000360502	PDE6C	1.38E-05	0.309	-	-
ENSP00000327704	PSMF1	1.24E-05	0.309	-	-

ENSP00000377197	HYDIN	1.22E-05	0.309	-	-
ENSP00000392147	PPP6C	2.10E-05	0.31	-	-
ENSP00000325527	FBN1	2.63E-05	0.311	-	-
ENSP00000239243	MSX2	1.95E-05	0.311	-	-
ENSP00000357484	GOPC	1.48E-05	0.311	-	-
ENSP00000345512	SEMA6A	1.04E-05	0.311	-	-
ENSP00000272164	WNT9A	1.00E-05	0.311	-	-
ENSP00000294258	ZFPL1	1.05E-05	0.312	-	-
ENSP00000348784	IGBP1	2.34E-05	0.313	-	-
ENSP00000349085	MAGEA1	2.15E-05	0.313	-	-
ENSP00000285930	AKR1B1	1.92E-05	0.313	-	-
ENSP00000255192	BHMT2	1.59E-05	0.313	-	-
ENSP00000355069	PAX2	3.86E-05	0.315	-	-
ENSP00000316772	HMHA1	2.00E-05	0.315	-	-
ENSP00000349577	PRODH	1.97E-05	0.315	-	-
ENSP00000365682	TLE1	1.70E-05	0.315	-	-
ENSP00000357879	PSMD4	3.03E-05	0.316	-	-
ENSP00000303634	LRP8	1.50E-05	0.316	-	-
ENSP00000221419	HNRNPL	1.42E-05	0.316	-	-
ENSP00000222982	CYP3A5	3.93E-05	0.317	-	-
ENSP00000346300	CRKL	2.66E-05	0.317	-	-
ENSP00000322460	SCN4B	1.15E-05	0.317	-	-

ENSP00000221486	RNASEH2A	4.53E-05	0.318	-	-
ENSP00000229277	ENO2	4.22E-05	0.318	-	-
ENSP00000274376	RASA1	2.70E-05	0.318	-	-
ENSP00000363868	ABCA1	2.69E-05	0.318	-	-
ENSP00000302716	MUC17	2.24E-05	0.318	-	-
ENSP00000340691	EIF4EBP1	1.94E-05	0.318	-	-
ENSP00000346155	UCKL1	1.41E-05	0.318	-	-
ENSP00000354621	SMURF1	2.16E-05	0.319	-	-
ENSP00000279386	TBX6	1.18E-05	0.319	-	-
ENSP00000419923	KLF6	1.17E-05	0.319	-	-
ENSP00000328789	AP1S2	1.11E-05	0.319	-	-
ENSP00000290015	WNT9B	1.08E-05	0.32	-	-
ENSP00000322270	LPHN2	1.01E-05	0.32	-	-
ENSP00000356357	LHX9	1.68E-05	0.321	-	-
ENSP00000354478	DLX1	1.32E-05	0.321	-	-
ENSP00000314441	METTL1	1.96E-05	0.322	-	-
ENSP00000366306	SPRY2	1.74E-05	0.322	-	-
ENSP00000244137	PEPD	1.51E-05	0.322	-	-
ENSP00000332293	ASCL2	1.21E-05	0.322	-	-
ENSP00000349467	CALM1	1.08E-04	0.323	-	-
ENSP00000341838	TNNI3	2.13E-05	0.323	-	-
ENSP00000368966	TRPC3	1.35E-05	0.323	-	-

ENSP00000296271	RHO	4.60E-05	0.324	-	-
ENSP00000267803	DUOXA1	1.66E-05	0.324	-	-
ENSP00000290552	IRX6	1.02E-05	0.324	-	-
ENSP00000367959	HTR2A	1.82E-05	0.325	-	-
ENSP00000359290	DR1	1.66E-05	0.325	-	-
ENSP00000321835	ETV4	1.31E-05	0.325	-	-
ENSP00000369292	SAMD9	1.13E-05	0.325	-	-
ENSP00000227471	UNC93B1	1.13E-05	0.325	-	-
ENSP00000249647	SNAP23	1.96E-05	0.326	-	-
ENSP00000260950	MSTN	1.60E-05	0.326	-	-
ENSP00000235329	MFN2	1.43E-05	0.326	-	-
ENSP00000263735	EPCAM	1.18E-05	0.326	-	-
ENSP00000354532	PNP	1.11E-05	0.326	-	-
ENSP00000314151	KLK3	2.75E-05	0.327	-	-
ENSP00000245323	EFNB2	2.00E-05	0.327	-	-
ENSP00000312673	GH1	1.79E-05	0.327	-	-
ENSP00000265164	CASP6	1.09E-05	0.327	-	-
ENSP00000355231	BECN1	2.20E-05	0.328	-	-
ENSP00000244661	HIST1H3B	1.35E-05	0.328	-	-
ENSP00000297323	ADCY1	3.10E-05	0.329	-	-
ENSP00000361512	PRPS1	1.24E-05	0.329	-	-
ENSP00000263574	APLP2	1.05E-05	0.329	-	-

ENSP00000251038	ZC3H14	4.42E-05	0.33	-	-
ENSP00000282441	YAP1	1.71E-05	0.33	-	-
ENSP00000282957	CPB1	1.25E-05	0.33	-	-
ENSP00000222382	CYP3A43	3.76E-05	0.331	-	-
ENSP00000171887	TNS1	2.10E-05	0.331	-	-
ENSP00000320768	RCAN1	1.14E-05	0.331	-	-
ENSP00000268171	FURIN	4.01E-05	0.332	-	-
ENSP00000240423	NCAPH	1.94E-05	0.332	-	-
ENSP00000345708	KCNJ11	1.90E-05	0.332	-	-
ENSP00000300060	ANPEP	1.46E-05	0.332	-	-
ENSP00000365651	NPPB	1.13E-05	0.332	-	-
ENSP00000362314	EIF4EBP2	1.02E-05	0.332	-	-
ENSP00000352119	HIST1H2AG	1.01E-05	0.333	-	-
ENSP00000388996	AP1M1	1.67E-05	0.334	-	-
ENSP00000349486	SCRIB	1.51E-05	0.335	-	-
ENSP00000268957	TOB1	1.33E-05	0.335	-	-
ENSP00000292035	MED27	1.09E-05	0.335	-	-
ENSP00000371471	RSAD2	1.74E-05	0.336	-	-
ENSP00000376322	HOXD13	1.23E-05	0.336	-	-
ENSP00000330218	SOX1	1.09E-05	0.336	-	-
ENSP00000261712	PSMD11	1.62E-05	0.337	-	-
ENSP00000360761	AGPAT2	1.33E-05	0.337	-	-

ENSP00000339007	GRB2	9.24E-05	0.338	-	-
ENSP00000354522	TOP1	3.28E-05	0.338	-	-
ENSP00000171111	KEAP1	1.50E-05	0.338	-	-
ENSP00000382342	ABCC1	1.49E-05	0.338	-	-
ENSP00000331602	PRKCD	2.78E-05	0.339	-	-
ENSP00000303727	CHRNA7	1.12E-05	0.339	-	-
ENSP00000355536	MTR	2.24E-05	0.34	-	-
ENSP00000394085	JMJD6	1.11E-05	0.34	-	-
ENSP00000357206	NES	3.34E-05	0.341	-	-
ENSP00000380308	DYNC1I2	2.66E-05	0.341	-	-
ENSP00000374205	TBR1	1.29E-05	0.341	-	-
ENSP00000369442	KL	1.29E-05	0.342	-	-
ENSP00000302728	GUSB	1.22E-05	0.342	-	-
ENSP00000216194	ADSL	2.97E-05	0.343	-	-
ENSP00000288135	KIT	2.16E-05	0.343	-	-
ENSP00000317566	LCORL	1.55E-05	0.344	-	-
ENSP00000301790	HRASLS5	1.58E-05	0.345	-	-
ENSP00000384554	CLDN5	2.26E-05	0.346	-	-
ENSP00000222308	FKBP8	1.11E-05	0.346	-	-
ENSP00000338348	UBE2G2	1.66E-05	0.347	-	-
ENSP00000342626	EYA1	1.56E-05	0.347	-	-
ENSP00000307491	WDR48	1.17E-05	0.347	-	-

ENSP00000381066	MAP2K7	1.39E-05	0.348	-	-
ENSP00000350720	SMARCA4	4.31E-05	0.349	-	-
ENSP00000361554	TIE1	2.01E-05	0.349	-	-
ENSP00000342557	IL4I1	1.33E-05	0.349	-	-
ENSP00000306330	YWHAG	4.17E-05	0.35	-	-
ENSP00000349437	IGF2R	2.04E-05	0.35	-	-
ENSP00000382697	ROCK1	1.51E-05	0.35	-	-
ENSP00000254066	RARA	3.26E-05	0.351	-	-
ENSP00000372170	MSX1	2.27E-05	0.351	-	-
ENSP00000314508	GBA	1.61E-05	0.351	-	-
ENSP00000248673	ZFP36	1.59E-05	0.351	-	-
ENSP00000265372	CREM	2.04E-05	0.352	-	-
ENSP00000264039	GPC1	1.33E-05	0.352	-	-
ENSP00000251047	LMAN1	1.30E-05	0.352	-	-
ENSP00000302021	MUC7	1.29E-05	0.352	-	-
ENSP00000241337	GSTM2	1.04E-05	0.352	-	-
ENSP00000310998	NAGPA	2.30E-05	0.353	-	-
ENSP00000380252	NFE2L2	1.85E-05	0.353	-	-
ENSP00000338864	RASGRP2	1.26E-05	0.353	-	-
ENSP00000264010	CTCF	2.31E-05	0.354	-	-
ENSP00000412292	DEPDC1	1.71E-05	0.354	-	-
ENSP00000317580	NEUROG1	1.59E-05	0.354	-	-

ENSP00000339527	FOXO3	1.16E-05	0.354	-	-
ENSP00000349393	LIG4	1.80E-05	0.355	-	-
ENSP00000280346	DLAT	1.79E-05	0.355	-	-
ENSP00000352561	CALCR	1.68E-05	0.355	-	-
ENSP00000350911	CTSE	1.64E-05	0.355	-	-
ENSP00000337838	RTN4	1.64E-05	0.355	-	-
ENSP00000309591	PRKACA	3.58E-05	0.356	-	-
ENSP00000359854	GPC3	2.14E-05	0.356	-	-
ENSP00000355167	HORMAD1	1.58E-05	0.356	-	-
ENSP00000296161	DTX3L	1.16E-05	0.356	-	-
ENSP00000232003	HRG	1.25E-05	0.357	-	-
ENSP00000264059	EFHD1	1.18E-05	0.357	-	-
ENSP00000356832	SGK1	1.91E-05	0.359	-	-
ENSP00000307432	SUCLG2	1.58E-05	0.359	-	-
ENSP00000391998	ARTN	1.46E-05	0.36	-	-
ENSP00000301732	ABCA3	1.36E-05	0.36	-	-
ENSP00000385722	INTS1	1.14E-05	0.361	-	-
ENSP00000333266	UBA7	1.01E-05	0.361	-	-
ENSP00000229264	GNB3	1.67E-05	0.362	-	-
ENSP00000285238	ABCC3	1.33E-05	0.362	-	-
ENSP00000333994	HBB	1.89E-05	0.363	-	-
ENSP00000228644	MYF5	1.75E-05	0.363	-	-

ENSP00000362508	UGT1A4	1.10E-05	0.363	-	-
ENSP00000262018	SGCA	1.04E-05	0.363	-	-
ENSP00000281923	MGAT5	2.06E-05	0.364	-	-
ENSP00000269141	CDH2	3.14E-05	0.365	-	-
ENSP00000261170	GUCY2C	2.26E-05	0.365	-	-
ENSP00000235382	RGS2	1.74E-05	0.365	-	-
ENSP00000264741	ITGA9	1.58E-05	0.365	-	-
ENSP00000248935	GSTT1	1.18E-05	0.365	-	-
ENSP00000230053	B3GAT2	1.01E-05	0.365	-	-
ENSP00000355153	CDKN2A	4.96E-05	0.366	-	-
ENSP00000365811	SPAG6	2.37E-05	0.366	-	-
ENSP00000262133	RBL2	1.37E-05	0.366	-	-
ENSP00000369810	VPS16	1.14E-05	0.366	-	-
ENSP00000300935	RAB8A	2.36E-05	0.367	-	-
ENSP00000292807	AP2M1	2.78E-05	0.368	-	-
ENSP00000357669	NPR1	2.27E-05	0.368	-	-
ENSP00000250244	AP1M2	1.29E-05	0.368	-	-
ENSP00000257904	CDK4	5.04E-05	0.369	-	-
ENSP00000369816	SHBG	2.00E-05	0.369	-	-
ENSP00000355747	PSEN2	1.73E-05	0.369	-	-
ENSP00000261523	RORA	1.47E-05	0.369	-	-
ENSP00000343325	PKN1	2.72E-05	0.37	-	-

ENSP00000286621	ADK	1.30E-05	0.37	-	-
ENSP00000280362	PTS	1.96E-05	0.371	-	-
ENSP00000249499	HOXD9	1.32E-05	0.371	-	-
ENSP00000344609	BTG3	1.15E-05	0.371	-	-
ENSP00000418532	UGT1A3	1.06E-05	0.371	-	-
ENSP00000362549	UGT1A8	1.04E-05	0.371	-	-
ENSP00000313681	SPHK1	1.52E-05	0.372	-	-
ENSP00000290953	AGRP	1.13E-05	0.372	-	-
ENSP00000301585	ICT1	3.20E-05	0.373	-	-
ENSP00000396439	RING1	1.56E-05	0.373	-	-
ENSP00000384211	PSME4	1.32E-05	0.373	-	-
ENSP00000320493	TRIP10	1.31E-05	0.373	-	-
ENSP00000358043	ECM1	1.28E-05	0.373	-	-
ENSP00000413234	AP2A2	1.12E-05	0.373	-	-
ENSP00000284384	PRKCA	4.24E-05	0.374	-	-
ENSP00000284523	WNT3A	2.86E-05	0.374	-	-
ENSP00000360290	PRKAA2	1.32E-05	0.374	-	-
ENSP00000366460	PLXDC2	1.10E-05	0.374	-	-
ENSP00000363763	EPHB2	1.78E-05	0.375	-	-
ENSP00000361669	ESX1	1.55E-05	0.375	-	-
ENSP00000338235	MTDH	1.22E-05	0.375	-	-
ENSP00000339191	CAV1	3.25E-05	0.376	-	-

ENSP00000417257	FNDC3A	2.94E-05	0.376	-	-
ENSP00000302830	MAEA	1.94E-05	0.376	-	-
ENSP00000241124	GJB6	1.39E-05	0.376	-	-
ENSP00000263038	PHYH	1.09E-05	0.376	-	-
ENSP00000347088	LARGE	1.46E-05	0.378	-	-
ENSP00000377148	AP1G1	1.46E-05	0.378	-	-
ENSP00000394794	PTPN13	1.14E-05	0.378	-	-
ENSP00000223140	NOBOX	1.03E-05	0.379	-	-
ENSP00000377941	ACTN1	2.29E-05	0.381	-	-
ENSP00000335153	HSP90AA1	4.30E-05	0.382	-	-
ENSP00000257860	PRPH	1.25E-05	0.382	-	-
ENSP00000309572	TERT	2.58E-05	0.383	-	-
ENSP00000357838	OAT	1.85E-05	0.383	-	-
ENSP00000361824	SPTAN1	2.01E-05	0.384	-	-
ENSP00000300177	GREM1	1.35E-05	0.384	-	-
ENSP00000368552	SIRT5	1.31E-05	0.384	-	-
ENSP00000352408	BABAM1	1.46E-05	0.385	-	-
ENSP00000417281	MDM2	4.48E-05	0.386	-	-
ENSP00000262850	RRAGB	1.51E-05	0.386	-	-
ENSP00000217402	CHMP4B	1.32E-05	0.386	-	-
ENSP00000326366	PSEN1	2.58E-05	0.387	-	-
ENSP00000249750	ALDH1A2	1.49E-05	0.387	-	-

ENSP00000263182	BBOX1	3.15E-05	0.39	-	-
ENSP00000369654	HBD	1.48E-05	0.39	-	-
ENSP00000359285	CHRNA4	1.27E-05	0.39	-	-
ENSP00000349959	RICTOR	2.21E-05	0.392	-	-
ENSP00000368169	DVL1	2.98E-05	0.393	-	-
ENSP00000252699	ACTN4	2.53E-05	0.394	-	-
ENSP00000322898	EBF1	1.79E-05	0.394	-	-
ENSP00000332247	ATP6V0A2	1.25E-05	0.394	-	-
ENSP00000258106	EMX1	1.15E-05	0.394	-	-
ENSP00000343838	UGT1A10	1.04E-05	0.394	-	-
ENSP00000353013	CLEC14A	1.02E-05	0.394	-	-
ENSP00000262958	GNA15	2.23E-05	0.396	-	-
ENSP00000294312	FGF19	2.02E-05	0.396	-	-
ENSP00000239462	TNN	1.61E-05	0.396	-	-
ENSP00000355173	ATG9A	1.06E-05	0.396	-	-
ENSP00000237612	ABCG2	2.36E-05	0.397	-	-
ENSP00000257566	TBX3	1.24E-05	0.397	-	-
ENSP00000254657	PER2	1.12E-05	0.397	-	-
ENSP00000234701	CLCA1	1.08E-05	0.397	-	-
ENSP00000365807	GNA14	1.31E-05	0.398	-	-
ENSP00000362525	UGT1A7	1.06E-05	0.398	-	-
ENSP00000222248	SLC5A5	2.37E-05	0.399	-	-

ENSP00000281821	EPHA4	1.45E-05	0.399	-	-
ENSP00000373487	KRT18	1.09E-05	0.399	-	-
ENSP00000341382	SMC4	2.35E-05	0.4	-	-
ENSP00000314499	GAK	1.39E-05	0.4	-	-
ENSP00000342007	CYP1A2	2.16E-05	0.401	-	-
ENSP00000277865	GLUD1	1.65E-05	0.401	-	-
ENSP00000306129	DRD5	1.62E-05	0.401	-	-
ENSP00000344460	CBS	2.12E-05	0.402	-	-
ENSP00000261303	PSMC1	1.76E-05	0.402	-	-
ENSP00000345096	IMPDH1	1.38E-05	0.402	-	-
ENSP00000408411	LAMP2	2.21E-05	0.404	-	-
ENSP00000380178	UBE2G1	1.65E-05	0.404	-	-
ENSP00000234296	ORC2	1.45E-05	0.404	-	-
ENSP00000356999	USF1	1.38E-05	0.404	-	-
ENSP00000356000	PLXNA2	1.02E-05	0.404	-	-
ENSP00000286548	GNAQ	2.01E-05	0.405	-	-
ENSP00000008527	CRY1	1.09E-05	0.406	-	-
ENSP00000338082	HBG2	1.04E-05	0.406	-	-
ENSP00000296641	F2RL2	1.29E-05	0.407	-	-
ENSP00000362513	UGT1A5	1.08E-05	0.407	-	-
ENSP00000295902	PRICKLE2	1.07E-05	0.407	-	-
ENSP00000219313	PSMD7	1.68E-05	0.408	-	-

ENSP00000302967	HDAC3	4.89E-05	0.409	-	-
ENSP00000346768	UGT1A9	1.07E-05	0.409	-	-
ENSP00000316176	UBE2N	2.84E-05	0.41	-	-
ENSP00000247182	SIX1	1.07E-05	0.411	-	-
ENSP00000301774	BEST1	1.04E-05	0.411	-	-
ENSP00000372654	AQP4	1.09E-05	0.412	-	-
ENSP00000222271	COMP	3.16E-05	0.413	-	-
ENSP00000355195	ASB12	1.23E-05	0.413	-	-
ENSP00000348461	RAC1	7.45E-05	0.414	-	-
ENSP00000386165	CEBPD	2.03E-05	0.414	-	-
ENSP00000350616	DDC	1.32E-05	0.414	-	-
ENSP00000326371	FOXC2	1.30E-05	0.415	-	-
ENSP00000346437	ATG7	1.20E-05	0.415	-	-
ENSP00000265012	GCNT2	1.08E-05	0.415	-	-
ENSP00000340510	PPL	1.04E-05	0.415	-	-
ENSP00000390948	CYFIP2	1.01E-05	0.415	-	-
ENSP00000363019	UBE2D1	1.84E-05	0.416	-	-
ENSP00000261733	ALDH2	1.47E-05	0.416	-	-
ENSP00000362768	RBL1	1.07E-05	0.416	-	-
ENSP00000262320	AXIN1	2.91E-05	0.417	-	-
ENSP00000349595	ATP2A1	1.61E-05	0.417	-	-
ENSP00000264717	GCKR	1.17E-05	0.417	-	-

ENSP00000352401	RPE	1.35E-05	0.418	-	-
ENSP00000297347	MED30	1.01E-05	0.418	-	-
ENSP00000265709	ANK1	4.00E-05	0.419	-	-
ENSP00000265723	ABCB4	1.22E-05	0.419	-	-
ENSP00000323516	UTS2R	1.56E-05	0.42	-	-
ENSP00000261740	TRPV4	1.15E-05	0.42	-	-
ENSP00000279227	FERMT3	1.10E-05	0.42	-	-
ENSP00000363851	EDA2R	1.78E-05	0.421	-	-
ENSP00000350894	SERPINH1	1.15E-05	0.421	-	-
ENSP00000389140	DCC	1.16E-05	0.422	-	-
ENSP00000217386	OXT	1.05E-05	0.422	-	-
ENSP00000262187	RHEB	1.17E-05	0.424	-	-
ENSP00000370023	HADHA	1.02E-05	0.424	-	-
ENSP00000297261	SHH	5.91E-05	0.426	-	-
ENSP00000278616	ATM	4.64E-05	0.426	-	-
ENSP00000375730	RPL13A	1.63E-05	0.426	-	-
ENSP00000325863	MRE11A	2.61E-05	0.427	-	-
ENSP00000265362	SEMA3A	1.24E-05	0.429	-	-
ENSP00000352839	NPSR1	2.11E-05	0.43	-	-
ENSP00000260970	PPIG	1.73E-05	0.43	-	-
ENSP00000283928	JAZF1	1.23E-05	0.43	-	-
ENSP00000349465	PICK1	1.23E-05	0.43	-	-

ENSP00000370330	ERBB2IP	1.12E-05	0.43	-	-
ENSP00000263904	STAM2	1.02E-05	0.43	-	-
ENSP00000265729	SRI	1.34E-05	0.431	-	-
ENSP00000324560	ULK1	1.13E-05	0.431	-	-
ENSP00000331201	HGS	2.42E-05	0.432	-	-
ENSP00000323588	SOX2	2.13E-05	0.432	-	-
ENSP00000221476	CKM	1.48E-05	0.432	-	-
ENSP00000349887	ASNA1	1.45E-05	0.432	-	-
ENSP00000240139	PPP3CC	1.14E-05	0.432	-	-
ENSP00000395473	MAN2B1	1.10E-05	0.432	-	-
ENSP00000287295	AIFM1	2.50E-05	0.433	-	-
ENSP00000054668	UTS2	1.94E-05	0.433	-	-
ENSP00000343819	OTX2	1.75E-05	0.433	-	-
ENSP00000263816	LRP2	1.00E-05	0.433	-	-
ENSP00000363680	EDA	3.34E-05	0.434	-	-
ENSP00000157812	PSMC4	2.02E-05	0.434	-	-
ENSP00000304845	UGT1A1	1.27E-05	0.434	-	-
ENSP00000354238	ENPP1	1.07E-05	0.434	-	-
ENSP00000257879	ITGA7	1.46E-05	0.435	-	-
ENSP00000226760	WFS1	1.09E-05	0.437	-	-
ENSP00000371138	FKBP1A	1.76E-05	0.438	-	-
ENSP00000173898	TRO	1.87E-05	0.44	-	-

ENSP00000347942	RET	3.55E-05	0.441	-	-
ENSP00000352336	PLCG2	1.95E-05	0.441	-	-
ENSP00000301740	SRRM2	1.51E-05	0.441	-	-
ENSP00000336666	AP1S1	1.14E-05	0.441	-	-
ENSP00000339801	IDS	2.66E-05	0.442	-	-
ENSP00000348307	SIRPA	2.17E-05	0.442	-	-
ENSP00000316779	BIN1	1.18E-05	0.442	-	-
ENSP00000346389	MEF2A	1.15E-05	0.442	-	-
ENSP00000311121	PSMA8	1.07E-05	0.442	-	-
ENSP00000262340	RPE65	1.04E-05	0.442	-	-
ENSP00000330349	DDX41	1.02E-05	0.442	-	-
ENSP00000331791	TBX1	1.37E-05	0.443	-	-
ENSP00000344479	NR4A2	1.34E-05	0.443	-	-
ENSP00000374998	IGHD	1.51E-05	0.444	-	-
ENSP00000252660	MAS1	2.24E-05	0.445	-	-
ENSP00000361418	IPO13	1.59E-05	0.445	-	-
ENSP00000368020	KIF3A	1.55E-05	0.445	-	-
ENSP00000340684	MAOA	1.12E-05	0.445	-	-
ENSP00000287936	HMGCR	1.82E-05	0.446	-	-
ENSP00000349705	TOP3B	1.20E-05	0.446	-	-
ENSP00000332353	PTCH1	2.55E-05	0.447	-	-
ENSP00000295113	FRZB	2.43E-05	0.447	-	-

ENSP00000348069	SREBF1	1.80E-05	0.447	-	-
ENSP00000307859	SLC22A18	1.22E-05	0.447	-	-
ENSP00000229307	NANOG	3.30E-05	0.449	-	-
ENSP00000298510	PRDX3	1.41E-05	0.449	-	-
ENSP00000262238	YY1	2.84E-05	0.45	-	-
ENSP00000248114	GFER	2.37E-05	0.45	-	-
ENSP00000260682	CYP2C9	1.61E-05	0.45	-	-
ENSP00000203786	ARHGAP4	1.16E-05	0.45	-	-
ENSP00000338629	RERE	1.73E-05	0.451	-	-
ENSP00000325526	WNT11	1.20E-05	0.451	-	-
ENSP00000233336	TTL	1.01E-05	0.451	-	-
ENSP00000262948	MAP2K2	1.46E-05	0.452	-	-
ENSP00000283179	HNRNPU	1.40E-05	0.452	-	-
ENSP00000243457	KCNJ2	1.04E-05	0.452	-	-
ENSP00000292644	PSMC2	3.24E-05	0.453	-	-
ENSP00000281043	MYCN	1.49E-05	0.453	-	-
ENSP00000322323	FAM20C	1.03E-05	0.453	-	-
ENSP00000316054	DVL3	1.87E-05	0.454	-	-
ENSP00000268613	CDH13	1.13E-05	0.455	-	-
ENSP00000310006	NR1D2	1.02E-05	0.455	-	-
ENSP00000269260	ARRB2	9.09E-05	0.456	-	-
ENSP00000315859	RNPS1	2.01E-05	0.456	-	-

ENSP00000363641	TXN	3.38E-05	0.457	-	-
ENSP00000257915	TFCP2	1.01E-05	0.457	-	-
ENSP00000401632	GSTT1	1.27E-05	0.458	-	-
ENSP00000332565	SULT4A1	1.94E-05	0.459	-	-
ENSP00000311449	RAB6A	1.43E-05	0.459	-	-
ENSP00000262735	PPARA	3.41E-05	0.46	-	-
ENSP00000383623	MLLT4	1.58E-05	0.46	-	-
ENSP00000181383	CPB2	1.40E-05	0.46	-	-
ENSP00000311005	MAPK7	1.13E-05	0.46	-	-
ENSP00000352673	ELF3	2.53E-05	0.461	-	-
ENSP00000247970	PIN1	2.44E-05	0.461	-	-
ENSP00000295797	PRKCI	1.26E-05	0.461	-	-
ENSP00000354877	ULK2	1.05E-05	0.461	-	-
ENSP00000365891	WAS	2.39E-05	0.464	-	-
ENSP00000278568	PAK1	2.27E-05	0.464	-	-
ENSP00000352324	ATXN3	1.44E-05	0.465	-	-
ENSP00000381282	VIMP	1.13E-05	0.465	-	-
ENSP00000366746	STAM	1.04E-05	0.466	-	-
ENSP00000372191	SIRT3	1.01E-05	0.466	-	-
ENSP00000312987	HNF4A	3.05E-05	0.467	-	-
ENSP00000346294	S100A4	1.43E-05	0.467	-	-
ENSP00000264187	NID1	1.18E-05	0.467	-	-

ENSP00000297375	EN2	1.69E-05	0.468	-	-
ENSP00000260947	BARD1	1.37E-05	0.468	-	-
ENSP00000347041	FMOD	1.98E-05	0.469	-	-
ENSP00000367309	MAOB	1.05E-05	0.469	-	-
ENSP00000317145	GDNF	2.10E-05	0.471	-	-
ENSP00000324856	STK11	1.67E-05	0.471	-	-
ENSP00000269216	GATA6	1.69E-05	0.472	-	-
ENSP00000264639	PSMD3	1.67E-05	0.472	-	-
ENSP00000257248	GIF	1.34E-05	0.472	-	-
ENSP00000317578	ADRBK2	5.37E-05	0.473	-	-
ENSP00000054666	VAMP3	1.75E-05	0.473	-	-
ENSP00000382064	DTNA	1.00E-05	0.473	-	-
ENSP00000307863	U2AF2	2.16E-05	0.474	-	-
ENSP00000364979	COL4A1	1.44E-05	0.474	-	-
ENSP00000368174	MCM8	5.07E-05	0.475	-	-
ENSP00000362298	SGPL1	1.17E-05	0.475	-	-
ENSP00000281537	TJP1	3.12E-05	0.476	-	-
ENSP00000317714	STX4	1.20E-05	0.476	-	-
ENSP00000293422	MYL6	1.09E-05	0.476	-	-
ENSP00000340879	RAD1	1.09E-05	0.476	-	-
ENSP00000348827	THRB	1.32E-05	0.478	-	-
ENSP00000271308	PSMA5	1.98E-05	0.479	-	-

ENSP00000296930	NPM1	3.34E-05	0.48	-	-
ENSP00000345530	NEDD4	1.98E-05	0.48	-	-
ENSP00000256104	FABP4	1.45E-05	0.481	-	-
ENSP00000400365	LAMA2	1.22E-05	0.481	-	-
ENSP00000319501	UGDH	1.13E-05	0.481	-	-
ENSP00000265022	DGKG	1.08E-05	0.482	-	-
ENSP00000306124	PRKCE	2.01E-05	0.483	-	-
ENSP00000298110	GPR101	1.18E-05	0.483	-	-
ENSP00000282549	OTX1	1.09E-05	0.483	-	-
ENSP00000309457	VPS41	1.09E-05	0.483	-	-
ENSP00000334115	OR10T2	1.05E-05	0.483	-	-
ENSP00000305424	OR1F1	1.06E-05	0.484	-	-
ENSP00000003084	CFTR	3.81E-05	0.485	-	-
ENSP00000369050	CYP1A1	2.08E-05	0.485	-	-
ENSP00000298032	ARMC3	1.13E-05	0.486	-	-
ENSP00000354511	COMT	2.19E-05	0.487	-	-
ENSP00000347717	DHCR7	1.19E-05	0.487	-	-
ENSP00000260653	SIX3	1.09E-05	0.487	-	-
ENSP00000342307	FOXM1	2.28E-05	0.488	-	-
ENSP00000302100	LGALS4	1.98E-05	0.488	-	-
ENSP00000332118	EPHB3	1.22E-05	0.488	-	-
ENSP00000374467	ABCC8	1.80E-05	0.489	-	-

ENSP00000361151	CEL	1.11E-05	0.489	-	-
ENSP00000261900	CCNT1	1.01E-05	0.489	-	-
ENSP00000358022	MCL1	3.28E-05	0.49	-	-
ENSP00000351486	NTRK1	1.06E-05	0.49	-	-
ENSP00000265094	FBXW11	1.20E-05	0.491	-	-
ENSP00000298552	TSC1	1.06E-05	0.491	-	-
ENSP00000328547	DNMT3B	4.19E-05	0.492	-	-
ENSP00000368798	MBTPS2	1.67E-05	0.492	-	-
ENSP00000344666	NF2	1.89E-05	0.493	-	-
ENSP00000217800	POLI	1.50E-05	0.493	-	-
ENSP00000396127	RAN	5.57E-05	0.494	-	-
ENSP00000249071	RAC2	4.48E-05	0.494	-	-
ENSP00000263043	ICK	2.89E-05	0.494	-	-
ENSP00000381504	PRPS2	1.13E-05	0.494	-	-
ENSP00000272065	ACP1	1.17E-05	0.495	-	-
ENSP00000320935	SLC2A4	1.29E-05	0.496	-	-
ENSP00000338413	UBA1	1.26E-05	0.496	-	-
ENSP00000200181	ITGB4	1.16E-05	0.496	-	-
ENSP00000295550	COL6A3	1.03E-05	0.496	-	-
ENSP00000279242	MRPL49	2.38E-05	0.497	-	-
ENSP00000248975	YWHAH	1.80E-05	0.497	-	-
ENSP00000369677	EMILIN1	1.71E-05	0.497	-	-

ENSP00000043402	RTN4R	1.44E-05	0.497	-	-
ENSP00000321636	TOP3A	1.61E-05	0.498	-	-
ENSP00000314414	AP2B1	1.33E-05	0.498	-	-
ENSP00000310928	PPARD	1.23E-05	0.498	-	-
ENSP00000321744	HIST1H2BC	1.27E-05	0.499	-	-
ENSP00000260795	FGFR3	1.66E-05	0.5	-	-
ENSP00000262854	HUWE1	1.69E-05	0.501	-	-
ENSP00000311165	AQP1	1.38E-05	0.501	-	-
ENSP00000319096	RAP2B	1.12E-05	0.501	-	-
ENSP00000299421	ILK	1.73E-05	0.502	-	-
ENSP00000336790	ATF4	2.04E-05	0.507	-	-
ENSP00000230658	ISL1	1.94E-05	0.507	-	-
ENSP00000270349	SLC6A3	2.38E-05	0.508	-	-
ENSP00000301843	CTTN	1.83E-05	0.508	-	-
ENSP00000287156	UBE2L6	1.34E-05	0.508	-	-
ENSP00000366244	ARAF	1.23E-05	0.509	-	-
ENSP00000247138	SLC35A2	1.46E-05	0.51	-	-
ENSP00000263864	VAMP8	1.06E-05	0.51	-	-
ENSP00000314343	MED29	1.18E-05	0.511	-	-
ENSP00000317955	EEA1	2.10E-05	0.512	-	-
ENSP00000259457	PSMB7	1.54E-05	0.513	-	-
ENSP00000343741	ATR	2.02E-05	0.514	-	-

ENSP00000243222	COL10A1	1.50E-05	0.514	-	-
ENSP00000295600	MITF	1.34E-05	0.514	-	-
ENSP00000262193	PSMB1	1.90E-05	0.515	-	-
ENSP00000345494	PLSCR1	1.15E-05	0.516	-	-
ENSP00000264710	RAB10	1.38E-05	0.517	-	-
ENSP00000265734	CDK6	1.64E-05	0.52	-	-
ENSP00000264709	DNMT3A	1.22E-05	0.52	-	-
ENSP00000240652	IAPP	1.10E-05	0.52	-	-
ENSP00000252945	CYP2E1	1.64E-05	0.521	-	-
ENSP00000295956	FLNB	1.10E-05	0.522	-	-
ENSP00000278833	ROM1	2.09E-05	0.523	-	-
ENSP00000258080	HTRA2	1.33E-05	0.523	-	-
ENSP00000396937	PSMD13	1.48E-05	0.524	-	-
ENSP00000253339	LATS1	1.07E-05	0.525	-	-
ENSP00000285379	CA2	3.65E-05	0.527	-	-
ENSP00000217958	PSMD10	2.15E-05	0.527	-	-
ENSP00000379616	MYH11	1.94E-05	0.527	-	-
ENSP00000358155	HIST2H2AA4	1.62E-05	0.527	-	-
ENSP00000355537	ACTN2	2.73E-05	0.528	-	-
ENSP00000334003	INTU	2.23E-05	0.529	-	-
ENSP00000352516	DNMT1	2.79E-05	0.53	-	-
ENSP00000341189	PTK2	1.88E-05	0.53	-	-

ENSP00000310129	PSMD2	1.86E-05	0.53	-	-
ENSP00000270225	SAE1	1.80E-05	0.53	-	-
ENSP00000320940	NCOA1	1.66E-05	0.53	-	-
ENSP00000416293	SLC2A1	1.48E-05	0.53	-	-
ENSP00000242576	UNG	1.43E-05	0.531	-	-
ENSP00000266744	ASCL1	1.27E-05	0.531	-	-
ENSP00000254810	H3F3B	4.93E-05	0.532	-	-
ENSP00000362334	PSMB2	1.63E-05	0.532	-	-
ENSP00000367207	MYC	7.25E-05	0.533	-	-
ENSP00000388340	CLINT1	1.64E-05	0.534	-	-
ENSP00000351926	AP2A1	1.35E-05	0.534	-	-
ENSP00000386259	NEB	1.32E-05	0.534	-	-
ENSP00000401445	ERN1	1.36E-05	0.535	-	-
ENSP00000362401	CYSLTR1	1.17E-05	0.535	-	-
ENSP00000338606	SMUG1	1.07E-05	0.535	-	-
ENSP00000321797	FGF8	1.38E-05	0.536	-	-
ENSP00000350275	HIST1H3A	1.26E-05	0.536	-	-
ENSP00000381105	NPS	1.14E-05	0.537	-	-
ENSP00000378890	GCH1	1.03E-05	0.537	-	-
ENSP00000260402	PLCB2	2.03E-05	0.538	-	-
ENSP00000368350	TPT1	1.59E-05	0.539	-	-
ENSP00000364475	FBP1	1.50E-05	0.539	-	-

ENSP00000331040	OLIG2	1.35E-05	0.54	-	-
ENSP00000272227	PDIA6	1.13E-05	0.54	-	-
ENSP00000262186	KCNH2	1.63E-05	0.541	-	-
ENSP00000323439	SMC6	1.08E-05	0.542	-	-
ENSP00000393887	AHSG	2.17E-05	0.543	-	-
ENSP00000372547	SRY	2.21E-05	0.544	-	-
ENSP00000352835	MB	1.14E-05	0.544	-	-
ENSP00000226413	GNRHR	1.12E-05	0.544	-	-
ENSP00000237596	PKD2	1.09E-05	0.544	-	-
ENSP00000291295	CALM3	7.91E-05	0.546	-	-
ENSP00000335306	DPPA4	1.50E-05	0.547	-	-
ENSP00000299767	HSP90B1	1.33E-05	0.548	-	-
ENSP00000350937	TES	2.51E-05	0.549	-	-
ENSP00000348108	KHDRBS3	1.30E-05	0.549	-	-
ENSP00000375921	PAX3	1.71E-05	0.55	-	-
ENSP00000367992	ESD	1.43E-05	0.55	-	-
ENSP00000354677	GPX7	1.29E-05	0.551	-	-
ENSP00000272298	CALM2	8.06E-05	0.552	-	-
ENSP00000396219	MEF2C	1.59E-05	0.552	-	-
ENSP00000318631	HSD17B6	2.99E-05	0.553	-	-
ENSP00000358158	HIST2H2AA3	1.63E-05	0.553	-	-
ENSP00000311113	JUP	2.08E-05	0.555	-	-

ENSP00000317985	ROCK2	1.10E-05	0.555	-	-
ENSP00000362690	NR5A1	1.56E-05	0.556	-	-
ENSP00000234111	ODC1	1.19E-05	0.556	-	-
ENSP00000328251	MINA	1.13E-05	0.556	-	-
ENSP00000310448	SART1	1.00E-05	0.556	-	-
ENSP00000252115	POLDIP3	1.17E-05	0.557	-	-
ENSP00000297848	COL14A1	2.36E-05	0.558	-	-
ENSP00000317659	GUK1	2.07E-05	0.558	-	-
ENSP00000266718	LUM	1.89E-05	0.558	-	-
ENSP00000336702	EIF5A	1.44E-05	0.56	-	-
ENSP00000402084	WEE1	1.10E-05	0.56	-	-
ENSP00000331817	ALYREF	2.60E-05	0.562	-	-
ENSP00000324897	UBE2I	3.25E-05	0.564	-	-
ENSP00000314067	PAK2	1.49E-05	0.566	-	-
ENSP00000250416	PARP2	1.36E-05	0.566	-	-
ENSP00000392330	BCAP31	1.28E-05	0.566	-	-
ENSP00000337103	CHAT	1.27E-05	0.566	-	-
ENSP00000352312	DNAH8	7.19E-05	0.568	-	-
ENSP00000298852	PSMC3	2.78E-05	0.568	-	-
ENSP00000349960	ACTB	4.75E-05	0.569	-	-
ENSP00000304408	COL3A1	2.17E-05	0.569	-	-
ENSP00000313420	PRKDC	2.12E-05	0.569	-	-

ENSP00000344456	CTNNB1	6.19E-05	0.57	-	-
ENSP00000290541	PSMB4	2.64E-05	0.57	-	-
ENSP00000303452	TRH	1.61E-05	0.574	-	-
ENSP00000308944	HLTF	1.05E-05	0.574	-	-
ENSP00000359778	ELTD1	1.90E-05	0.576	-	-
ENSP00000204604	CHRD	1.12E-05	0.576	-	-
ENSP00000292907	COX7A1	1.35E-05	0.577	-	-
ENSP00000291901	TNNT1	1.08E-05	0.577	-	-
ENSP00000288266	APPL1	4.32E-05	0.578	-	-
ENSP00000248272	GAN	1.19E-05	0.578	-	-
ENSP00000303500	PAH	1.02E-05	0.58	-	-
ENSP00000359719	PRKACB	1.18E-05	0.581	-	-
ENSP00000366347	NKX2-2	1.07E-05	0.581	-	-
ENSP00000264335	YWHAE	2.45E-05	0.583	-	-
ENSP00000310572	PSMC5	2.19E-05	0.585	-	-
ENSP00000282486	MBNL1	1.99E-05	0.585	-	-
ENSP00000228945	ARHGDIB	1.02E-05	0.585	-	-
ENSP00000259512	DERL1	1.00E-05	0.585	-	-
ENSP00000278823	MTA2	1.74E-05	0.586	-	-
ENSP00000256958	SLCO1B1	1.06E-05	0.588	-	-
ENSP00000267163	RB1	3.52E-05	0.59	-	-
ENSP00000363092	PRKG1	1.29E-05	0.59	-	-

ENSP00000260433	CYP19A1	1.15E-05	0.59	-	-
ENSP00000353608	DSC3	1.07E-05	0.59	-	-
ENSP00000219476	TSC2	1.60E-05	0.592	-	-
ENSP00000250448	FOXA1	1.03E-05	0.592	-	-
ENSP00000305603	FUT3	1.82E-05	0.595	-	-
ENSP00000367623	HSPA14	1.03E-05	0.595	-	-
ENSP00000297518	CDK5	2.83E-05	0.599	-	-
ENSP00000333950	FMN1	1.30E-05	0.599	-	-
ENSP00000361141	OPN4	1.01E-05	0.599	-	-
ENSP00000338072	AVPR2	3.04E-05	0.6	-	-
ENSP00000267996	TPM1	2.15E-05	0.6	-	-
ENSP00000359478	ABCC2	1.27E-05	0.6	-	-
ENSP00000266376	CACNA1C	1.27E-05	0.6	-	-
ENSP00000355560	TBCE	1.49E-05	0.601	-	-
ENSP00000331327	WT1	1.47E-05	0.601	-	-
ENSP00000255380	CHRM3	1.02E-05	0.602	-	-
ENSP00000264552	UBE2S	2.06E-05	0.603	-	-
ENSP00000332151	DSE	1.50E-05	0.606	-	-
ENSP00000359206	BTRC	1.58E-05	0.608	-	-
ENSP00000350336	DSCR8	1.34E-05	0.608	-	-
ENSP00000291552	U2AF1	1.03E-05	0.608	-	-
ENSP00000253363	RBM39	1.01E-05	0.608	-	-

ENSP00000262304	PKD1	1.01E-05	0.608	-	-
ENSP00000355778	H3F3A	4.88E-05	0.611	-	-
ENSP00000254108	FUS	1.71E-05	0.611	-	-
ENSP00000379865	ADHFE1	1.55E-05	0.611	-	-
ENSP00000078429	GNA11	1.36E-05	0.611	-	-
ENSP00000384026	HMGA2	1.48E-05	0.612	-	-
ENSP00000263360	EED	2.99E-05	0.613	-	-
ENSP00000314214	VAMP2	1.33E-05	0.613	-	-
ENSP00000306606	ADH1B	1.08E-05	0.613	-	-
ENSP00000363779	IKBKAP	1.69E-05	0.615	-	-
ENSP00000278715	HMBS	1.39E-05	0.615	-	-
ENSP00000295901	PSMD6	1.72E-05	0.616	-	-
ENSP00000212015	SIRT1	4.17E-05	0.618	-	-
ENSP00000320171	PKM	3.19E-05	0.618	-	-
ENSP00000366013	GNB2L1	2.88E-05	0.618	-	-
ENSP00000302051	ECEL1	1.25E-05	0.618	-	-
ENSP00000383199	NEDD4L	1.17E-05	0.619	-	-
ENSP00000270586	PSMB6	2.07E-05	0.62	-	-
ENSP00000306920	GLB1	1.47E-05	0.62	-	-
ENSP00000262306	TCEB2	1.26E-05	0.62	-	-
ENSP00000359211	DPYD	1.11E-05	0.621	-	-
ENSP00000296456	APEH	1.40E-05	0.622	-	-

ENSP00000225964	COL1A1	2.39E-05	0.623	-	-
ENSP00000211998	VCL	2.09E-05	0.623	-	-
ENSP00000344254	FXYD5	1.33E-05	0.623	-	-
ENSP00000360248	ENTPD1	1.22E-05	0.623	-	-
ENSP00000262056	EIF4B	1.16E-05	0.623	-	-
ENSP00000344871	MYO1F	2.64E-05	0.624	-	-
ENSP00000225426	PSMB3	1.77E-05	0.624	-	-
ENSP00000400175	RHOA	1.76E-05	0.624	-	-
ENSP00000368401	PAX6	1.63E-05	0.624	-	-
ENSP00000326804	CUL1	2.56E-05	0.625	-	-
ENSP00000242480	EGR2	1.26E-05	0.625	-	-
ENSP00000215574	CDC34	1.18E-05	0.625	-	-
ENSP00000250003	MYOD1	3.64E-05	0.626	-	-
ENSP00000245441	NIN	2.00E-05	0.626	-	-
ENSP00000256010	NTS	3.72E-05	0.627	-	-
ENSP00000396308	DHFR	1.96E-05	0.627	-	-
ENSP00000367787	LIG3	1.19E-05	0.627	-	-
ENSP00000252971	MNX1	2.69E-05	0.628	-	-
ENSP00000261427	UBE2K	2.54E-05	0.628	-	-
ENSP00000244769	ATXN1	1.97E-05	0.628	-	-
ENSP00000263270	AP2S1	1.47E-05	0.628	-	-
ENSP00000243050	NR4A1	1.05E-05	0.628	-	-

ENSP00000392423	RELN	1.31E-05	0.629	-	-
ENSP00000347251	GPS1	1.19E-05	0.629	-	-
ENSP00000337261	ARHGEF1	1.08E-05	0.629	-	-
ENSP00000362888	ADO	1.06E-05	0.629	-	-
ENSP00000262843	MID2	1.02E-05	0.63	-	-
ENSP00000334458	GATA4	2.30E-05	0.631	-	-
ENSP00000405798	CDK16	1.36E-05	0.631	-	-
ENSP00000354995	ABCG1	1.01E-05	0.632	-	-
ENSP00000290399	SIM2	1.02E-05	0.633	-	-
ENSP00000350132	PLN	1.06E-05	0.635	-	-
ENSP00000238081	YWHAQ	2.96E-05	0.636	-	-
ENSP00000264731	TP63	2.29E-05	0.636	-	-
ENSP00000369335	FBXO18	1.25E-05	0.636	-	-
ENSP00000355296	LDB3	1.12E-05	0.637	-	-
ENSP00000367545	TP73	1.37E-05	0.64	-	-
ENSP00000264162	LCT	1.75E-05	0.643	-	-
ENSP00000362649	HDAC1	5.71E-05	0.644	-	-
ENSP00000369643	GRPR	1.04E-05	0.644	-	-
ENSP00000384675	SOS1	1.90E-05	0.645	-	-
ENSP00000341940	CAV3	1.25E-05	0.645	-	-
ENSP00000263754	KAT2B	3.12E-05	0.648	-	-
ENSP00000248553	HSPB1	1.13E-05	0.648	-	-

ENSP00000173229	NTN1	1.09E-05	0.649	-	-
ENSP00000370083	SMN1	1.08E-05	0.649	-	-
ENSP00000355325	PSMB5	2.03E-05	0.65	-	-
ENSP00000312435	DAG1	1.24E-05	0.651	-	-
ENSP00000246554	COX6B1	1.07E-05	0.652	-	-
ENSP00000355512	OPN3	1.57E-05	0.655	-	-
ENSP00000351490	MAX	1.47E-05	0.656	-	-
ENSP00000044462	PSMA4	2.72E-05	0.658	-	-
ENSP00000368646	PRDX4	1.27E-05	0.658	-	-
ENSP00000238738	RHOQ	1.24E-05	0.658	-	-
ENSP00000343745	DICER1	1.97E-05	0.66	-	-
ENSP00000262294	TRIM37	1.62E-05	0.661	-	-
ENSP00000358857	EMD	1.58E-05	0.661	-	-
ENSP00000261707	SLC6A4	2.04E-05	0.663	-	-
ENSP00000225698	C1QBP	3.96E-05	0.664	-	-
ENSP00000262746	PRDX1	1.58E-05	0.664	-	-
ENSP00000222115	HAS1	1.29E-05	0.665	-	-
ENSP00000230671	SLC6A7	2.69E-05	0.666	-	-
ENSP00000416320	NTM	1.01E-05	0.666	-	-
ENSP00000155840	KCNQ1	1.72E-05	0.667	-	-
ENSP00000356918	STX7	1.21E-05	0.668	-	-
ENSP00000305355	PRKCB	1.40E-05	0.669	-	-

ENSP00000392466	LDB1	1.12E-05	0.669	-	-
ENSP00000378735	DNAJC6	1.05E-05	0.669	-	-
ENSP00000399968	NCOA2	2.19E-05	0.67	-	-
ENSP00000368824	CNKSR2	1.09E-05	0.67	-	-
ENSP00000332194	HIST2H2AC	1.65E-05	0.672	-	-
ENSP00000216254	ACO2	1.41E-05	0.672	-	-
ENSP00000358770	SLK	1.40E-05	0.672	-	-
ENSP00000266304	TEF	1.31E-05	0.674	-	-
ENSP00000367891	LGALS7	1.26E-05	0.674	-	-
ENSP00000244289	LIPE	1.20E-05	0.674	-	-
ENSP00000174621	TRPV1	1.06E-05	0.675	-	-
ENSP00000396843	NFIC	1.01E-05	0.675	-	-
ENSP00000401371	TIA1	1.90E-05	0.677	-	-
ENSP00000361047	ALG13	1.20E-05	0.677	-	-
ENSP00000417587	PEG10	1.05E-05	0.678	-	-
ENSP00000381045	UBE3A	1.92E-05	0.679	-	-
ENSP00000216181	MYH9	1.30E-05	0.681	-	-
ENSP00000266481	DNM1L	1.80E-05	0.682	-	-
ENSP00000370119	SMN2	1.12E-05	0.682	-	-
ENSP00000298139	WRN	1.52E-05	0.684	-	-
ENSP00000225402	AATF	3.04E-05	0.685	-	-
ENSP00000362481	UPRT	1.39E-05	0.685	-	-

ENSP00000346879	NKX2-1	1.12E-05	0.685	-	-
ENSP00000357047	MED23	1.10E-05	0.686	-	-
ENSP00000237527	GHRH	1.80E-05	0.69	-	-
ENSP00000362413	PGK1	2.83E-05	0.691	-	-
ENSP00000261245	MNAT1	1.18E-05	0.691	-	-
ENSP00000367034	HIST1H4C	1.97E-05	0.692	-	-
ENSP00000279230	PLCB3	1.34E-05	0.692	-	-
ENSP00000352929	CSNK1E	1.02E-05	0.692	-	-
ENSP00000355645	ACTA1	2.32E-05	0.694	-	-
ENSP00000268712	NCOR1	1.86E-05	0.695	-	-
ENSP00000368253	NR0B1	1.20E-05	0.695	-	-
ENSP00000217964	TBL1X	1.13E-05	0.695	-	-
ENSP00000332643	NDN	1.02E-05	0.696	-	-
ENSP00000359532	NTSR1	2.28E-05	0.697	-	-
ENSP00000347232	BLM	1.12E-05	0.697	-	-
ENSP00000238112	CPSF3	2.29E-05	0.698	-	-
ENSP00000257430	APC	1.34E-05	0.698	-	-
ENSP00000260985	IDH1	1.22E-05	0.699	-	-
ENSP00000363880	CUL2	1.14E-05	0.705	-	-
ENSP00000227667	APOC3	1.49E-05	0.706	-	-
ENSP00000263233	SYP	2.37E-05	0.708	-	-
ENSP00000362335	SAR1A	1.20E-05	0.708	-	-

ENSP00000354554	MT-CYB	1.33E-05	0.709	-	-
ENSP00000297268	COL1A2	1.88E-05	0.71	-	-
ENSP00000265354	SRF	2.41E-05	0.715	-	-
ENSP00000370571	TH	2.37E-05	0.716	-	-
ENSP00000347168	HIST1H4J	1.71E-05	0.717	-	-
ENSP00000358153	HIST2H4B	1.70E-05	0.718	-	-
ENSP00000266970	CDK2	4.17E-05	0.721	-	-
ENSP00000343282	HIST1H4D	1.71E-05	0.721	-	-
ENSP00000261479	PSMA6	1.64E-05	0.721	-	-
ENSP00000305958	STIP1	1.03E-05	0.721	-	-
ENSP00000350159	HIST1H4K	1.71E-05	0.723	-	-
ENSP00000346316	HIST1H4I	1.71E-05	0.723	-	-
ENSP00000382791	GRIK1	2.97E-05	0.724	-	-
ENSP00000253727	NR1H2	2.58E-05	0.724	-	-
ENSP00000351410	PRKAR1A	1.48E-05	0.725	-	-
ENSP00000216479	AHSA1	1.11E-05	0.725	-	-
ENSP00000229812	STK38	1.42E-05	0.726	-	-
ENSP00000348573	AKAP9	1.05E-05	0.726	-	-
ENSP00000327545	NPTXR	1.05E-05	0.727	-	-
ENSP00000265335	RAD50	1.52E-05	0.729	-	-
ENSP00000296412	ADH5	1.10E-05	0.729	-	-
ENSP00000353624	HIST1H4E	1.71E-05	0.731	-	-

ENSP00000220849	EIF3E	1.27E-05	0.731	-	-
ENSP00000369456	ITPA	1.28E-05	0.733	-	-
ENSP00000256383	EIF2S1	2.08E-05	0.734	-	-
ENSP00000358162	HIST2H4A	1.71E-05	0.738	-	-
ENSP00000324173	HSPA5	1.65E-05	0.739	-	-
ENSP00000334267	C5orf38	1.06E-05	0.741	-	-
ENSP00000266427	ETV6	2.22E-05	0.742	-	-
ENSP00000324464	CSNK1D	1.01E-05	0.742	-	-
ENSP00000362824	OGT	1.55E-05	0.743	-	-
ENSP00000337761	RAB27A	1.65E-05	0.745	-	-
ENSP00000358301	ADRB1	1.48E-05	0.746	-	-
ENSP00000297185	HSPA9	2.43E-05	0.749	-	-
ENSP00000290378	ACTC1	1.23E-05	0.749	-	-
ENSP00000373298	COLQ	1.89E-05	0.75	-	-
ENSP00000292476	CPSF4	1.33E-05	0.75	-	-
ENSP00000311997	NEFH	1.15E-05	0.75	-	-
ENSP00000269321	ARHGDIA	1.18E-05	0.751	-	-
ENSP00000366581	HIST1H4B	1.71E-05	0.752	-	-
ENSP00000339090	RBMX2	1.05E-05	0.753	-	-
ENSP00000349722	UBE2D3	1.03E-05	0.753	-	-
ENSP00000265070	GOLPH3	1.44E-05	0.757	-	-
ENSP00000231790	MLH1	1.49E-05	0.758	-	-

ENSP00000348442	PSMD12	1.74E-05	0.759	-	-
ENSP00000340278	PARK7	1.66E-05	0.759	-	-
ENSP00000261366	LMNB1	1.41E-05	0.76	-	-
ENSP00000252338	FAM155B	1.47E-05	0.761	-	-
ENSP00000257770	NT5E	2.27E-05	0.762	-	-
ENSP00000350767	HIST4H4	1.77E-05	0.763	-	-
ENSP00000280892	EIF4E	1.58E-05	0.763	-	-
ENSP00000164139	PYGM	1.80E-05	0.768	-	-
ENSP00000368438	PCNA	6.76E-05	0.77	-	-
ENSP00000248572	GNGT1	1.09E-05	0.771	-	-
ENSP00000348634	MYH6	1.17E-05	0.773	-	-
ENSP00000325875	HSP90AB1	1.15E-05	0.773	-	-
ENSP00000314458	CDC42	4.62E-05	0.774	-	-
ENSP00000320147	EZH2	1.75E-05	0.775	-	-
ENSP00000319690	HNRNPC	1.09E-05	0.775	-	-
ENSP00000216962	PYGB	1.45E-05	0.776	-	-
ENSP00000236959	ATIC	2.58E-05	0.778	-	-
ENSP00000262809	ELL	1.74E-05	0.778	-	-
ENSP00000363559	EIF6	1.59E-05	0.779	-	-
ENSP00000256682	ARF3	1.18E-05	0.781	-	-
ENSP00000401802	PSMC6	3.40E-05	0.782	-	-
ENSP00000383558	GCGR	1.28E-05	0.782	-	-

ENSP00000216392	PYGL	1.46E-05	0.783	-	-
ENSP00000359423	MTM1	1.11E-05	0.784	-	-
ENSP00000348258	HIST1H4L	1.75E-05	0.785	-	-
ENSP00000215071	PSMD8	1.72E-05	0.785	-	-
ENSP00000225792	DDX5	1.47E-05	0.786	-	-
ENSP00000264926	RAD18	1.13E-05	0.787	-	-
ENSP00000215904	PDXP	1.32E-05	0.79	-	-
ENSP00000322439	TUFM	1.34E-05	0.791	-	-
ENSP00000320604	FAXDC2	1.16E-05	0.791	-	-
ENSP00000369400	NHS	1.26E-05	0.792	-	-
ENSP00000262033	PTGES3	1.42E-05	0.794	-	-
ENSP00000264265	LXN	1.23E-05	0.794	-	-
ENSP00000369497	BRCA2	1.35E-05	0.795	-	-
ENSP00000220562	EXTL3	1.14E-05	0.795	-	-
ENSP00000209875	CBX5	1.22E-05	0.797	-	-
ENSP00000244711	MEA1	1.19E-05	0.804	-	-
ENSP00000300574	CRK	2.05E-05	0.805	-	-
ENSP00000217961	STS	1.88E-05	0.805	-	-
ENSP00000348984	SON	1.41E-05	0.807	-	-
ENSP00000265773	SMARCA2	1.82E-05	0.809	-	-
ENSP00000299293	FRS2	1.29E-05	0.81	-	-
ENSP00000330343	SUMO3	1.10E-05	0.81	-	-

ENSP00000359345	RPL5	2.16E-05	0.811	-	-
ENSP00000323155	DAND5	1.49E-05	0.811	-	-
ENSP00000361626	YBX1	1.14E-05	0.811	-	-
ENSP00000352980	HIST1H4A	1.77E-05	0.816	-	-
ENSP00000351284	RAD52	1.34E-05	0.817	-	-
ENSP00000265814	RUNX1T1	2.05E-05	0.819	-	-
ENSP00000245552	NT5C	1.20E-05	0.819	-	-
ENSP00000339992	MYB	2.06E-05	0.822	-	-
ENSP00000304592	FASN	1.29E-05	0.822	-	-
ENSP00000350283	BRCA1	3.91E-05	0.823	-	-
ENSP00000353622	SIN3A	1.72E-05	0.825	-	-
ENSP00000215567	TECR	1.39E-05	0.825	-	-
ENSP00000356433	UST	1.18E-05	0.826	-	-
ENSP00000381717	UBE2D2	1.09E-05	0.826	-	-
ENSP00000361777	SET	2.31E-05	0.831	-	-
ENSP00000360621	ORC1	1.44E-05	0.831	-	-
ENSP00000217971	PGRMC1	1.04E-05	0.831	-	-
ENSP00000222812	STX1A	1.03E-05	0.831	-	-
ENSP00000225430	RPL19	1.02E-05	0.835	-	-
ENSP00000351665	CLIP1	1.21E-05	0.841	-	-
ENSP00000366974	HIST1H4F	1.74E-05	0.842	-	-
ENSP00000217244	CSNK2A1	1.75E-05	0.843	-	-

ENSP00000269122	CLTC	1.39E-05	0.844	-	-
ENSP00000279593	GRIN2B	1.39E-05	0.845	-	-
ENSP00000419692	RXRA	1.34E-05	0.845	-	-
ENSP00000350249	POT1	1.00E-05	0.846	-	-
ENSP00000289352	HIST1H4H	1.75E-05	0.849	-	-
ENSP00000342374	SNRPD2	1.59E-05	0.849	-	-
ENSP00000376076	SUMO1	2.90E-05	0.85	-	-
ENSP00000351015	NF1	1.20E-05	0.85	-	-
ENSP00000298316	ARF6	1.02E-05	0.851	-	-
ENSP00000249042	TST	1.14E-05	0.857	-	-
ENSP00000039007	OTC	1.49E-05	0.858	-	-
ENSP00000310226	RAB1B	1.18E-05	0.858	-	-
ENSP00000262506	CSNK2A2	1.03E-05	0.858	-	-
ENSP00000376886	NME1-NME2	1.01E-05	0.859	-	-
ENSP00000363193	MED12	1.30E-05	0.86	-	-
ENSP00000231487	SKP1	2.68E-05	0.861	-	-
ENSP00000362592	RBBP4	1.15E-05	0.863	-	-
ENSP00000365877	SUV39H1	1.29E-05	0.865	-	-
ENSP00000297338	RAD21	1.46E-05	0.866	-	-
ENSP00000360532	CDC5L	2.10E-05	0.867	-	-
ENSP00000243253	SEC61A1	1.10E-05	0.867	-	-
ENSP00000360171	TENM1	1.09E-05	0.867	-	-

ENSP00000220959	UBR5	1.58E-05	0.868	-	-
ENSP00000263593	SIAE	1.20E-05	0.869	-	-
ENSP00000240922	NAA50	1.98E-05	0.87	-	-
ENSP00000252674	MLLT1	1.38E-05	0.875	-	-
ENSP00000227868	PDHX	1.17E-05	0.879	-	-
ENSP00000360154	OCRL	1.03E-05	0.879	-	-
ENSP00000334051	GNAL	1.32E-05	0.881	-	-
ENSP00000367408	CASK	1.53E-05	0.882	-	-
ENSP00000216455	PSMA3	1.94E-05	0.883	-	-
ENSP00000272317	RPS27A	1.47E-05	0.883	-	-
ENSP00000322159	ATG4C	1.15E-05	0.884	-	-
ENSP00000369427	RBBP7	1.53E-05	0.885	-	-
ENSP00000377446	SUCLG1	1.11E-05	0.887	-	-
ENSP00000362441	ATRX	1.04E-05	0.887	-	-
ENSP00000233893	HSPE1	1.30E-05	0.888	-	-
ENSP00000346566	CKAP5	2.63E-05	0.89	-	-
ENSP00000013034	NME1	1.87E-05	0.89	-	-
ENSP00000344936	PTTG1	1.09E-05	0.89	-	-
ENSP00000312288	HADH	1.85E-05	0.896	-	-
ENSP00000348577	RANGAP1	1.04E-05	0.896	-	-
ENSP00000350512	COPS5	2.42E-05	0.9	-	-
ENSP00000326261	SRRM1	1.09E-05	0.9	-	-

ENSP00000276072	TAF1	1.02E-05	0.901	-	-
ENSP00000276062	NDUFB11	1.16E-05	0.902	-	-
ENSP00000290158	KPNB1	1.06E-05	0.902	-	-
ENSP00000302896	RPS9	1.19E-05	0.905	-	-
ENSP00000381331	HDAC2	2.99E-05	0.906	-	-
ENSP00000216225	RBX1	1.17E-05	0.909	-	-
ENSP00000302955	RRM2	1.58E-05	0.911	-	-
ENSP00000369757	RPS6	1.64E-05	0.914	-	-
ENSP00000284811	TCEB1	1.25E-05	0.918	-	-
ENSP00000274255	SKP2	1.48E-05	0.922	-	-
ENSP00000265990	BTAF1	1.18E-05	0.924	-	-
ENSP00000419851	GMPS	2.05E-05	0.925	-	-
ENSP00000362576	YARS	1.04E-05	0.925	-	-
ENSP00000319169	PRMT5	1.20E-05	0.927	-	-
ENSP00000348762	LSS	1.10E-05	0.928	-	-
ENSP00000267868	RAD51	2.02E-05	0.931	-	-
ENSP00000260731	KIF11	1.62E-05	0.932	-	-
ENSP00000230354	TBP	1.48E-05	0.934	-	-
ENSP00000365572	NME2	1.03E-05	0.936	-	-
ENSP00000413572	NLE1	1.25E-05	0.939	-	-
ENSP00000316879	EIF4G1	1.11E-05	0.939	-	-
ENSP00000216911	AURKA	1.78E-05	0.941	-	-

ENSP00000286428	VBP1	1.41E-05	0.941	-	-
ENSP00000381031	EWSR1	1.27E-05	0.942	-	-
ENSP00000356448	TPR	1.07E-05	0.942	-	-
ENSP00000328236	KNTC1	2.21E-05	0.944	-	-
ENSP00000380414	DEK	1.10E-05	0.945	-	-
ENSP00000246548	UBA2	1.29E-05	0.946	-	-
ENSP00000386541	PSMD14	2.13E-05	0.947	-	-
ENSP00000258962	SRSF1	1.51E-05	0.947	-	-
ENSP00000262030	ATP5B	1.10E-05	0.948	-	-
ENSP00000321656	CDC25C	1.09E-05	0.949	-	-
ENSP00000344818	UBC	3.77E-04	0.954	-	-
ENSP00000278916	CHEK1	2.01E-05	0.954	-	-
ENSP00000359531	GTF2B	1.16E-05	0.954	-	-
ENSP00000245539	MRPS7	1.03E-05	0.955	-	-
ENSP00000395535	MECP2	1.27E-05	0.956	-	-
ENSP00000231572	RARS	1.15E-05	0.956	-	-
ENSP00000411532	TOP2A	1.56E-05	0.96	-	-
ENSP00000307940	EEF2	1.13E-05	0.96	-	-
ENSP00000223321	PSMA2	1.98E-05	0.962	-	-
ENSP00000303706	CDC25A	1.14E-05	0.962	-	-
ENSP00000365439	HNRNPK	1.16E-05	0.963	-	-
ENSP00000418447	PPP2CA	1.59E-05	0.964	-	-

ENSP00000261207	PPP1R12A	1.24E-05	0.965	-	-
ENSP00000327070	MDH2	1.20E-05	0.967	-	-
ENSP00000301764	DDB1	1.11E-05	0.967	-	-
ENSP00000275603	CCT6A	1.26E-05	0.968	-	-
ENSP00000315644	TYMS	1.57E-05	0.969	-	-
ENSP00000305480	FEN1	1.11E-05	0.969	-	-
ENSP00000364794	IARS	1.40E-05	0.97	-	-
ENSP00000397552	ACTL6A	1.26E-05	0.977	-	-
ENSP00000308405	H2AFV	1.06E-05	0.977	-	-
ENSP00000300738	RRM1	1.12E-05	0.981	-	-
ENSP00000363676	RPL11	1.09E-05	0.981	-	-
ENSP00000258091	CCT7	1.26E-05	0.982	-	-
ENSP00000234420	MSH6	1.26E-05	0.984	-	-
ENSP00000356425	UCHL5	1.11E-05	0.984	-	-
ENSP00000331310	MORF4L1	1.79E-05	0.986	-	-
ENSP00000357858	BUB3	1.38E-05	0.986	-	-
ENSP00000357244	CCT3	1.08E-05	0.986	-	-
ENSP00000007516	NDUFAB1	1.05E-05	0.987	-	-
ENSP00000256442	CCNB1	1.93E-05	0.988	-	-
ENSP00000385269	ELAVL1	3.51E-05	0.989	-	-
ENSP00000367276	CKAP2	2.27E-05	0.989	-	-
ENSP00000336741	DHX15	1.22E-05	0.989	-	-

ENSP00000364310	H2AFX	1.01E-05	0.989	-	-
ENSP00000382595	PAICS	1.22E-05	0.991	-	-
ENSP00000317334	TCP1	1.36E-05	0.992	-	-
ENSP00000296509	MAD2L1	1.23E-05	0.992	-	-
ENSP00000358563	DKC1	1.09E-05	0.992	-	-
ENSP00000314949	POLR2A	1.01E-05	0.992	-	-
ENSP00000299300	CCT2	1.42E-05	0.993	-	-
ENSP00000296417	H2AFZ	1.40E-05	0.993	-	-
ENSP00000326031	PPP1CA	1.18E-05	0.993	-	-
ENSP00000405965	SUMO2	2.15E-05	0.994	-	-
ENSP00000233146	MSH2	1.42E-05	0.994	-	-
ENSP00000296577	ABCE1	1.31E-05	0.994	-	-
ENSP00000300093	PLK1	1.14E-05	0.996	-	-
