# Supplementary material for: Determination of Genes Related to Uveitis by Utilization of the Random Walk with Restart Algorithm on a Protein–Protein Interaction Network
Source: Int J Mol Sci. 2017 May 13;18(5):1045. doi: 10.3390/ijms18051045 (PMC5454957; doi:10.3390/ijms18051045)
Supplement: Supplementary file 1 [file ijms-18-01045-s001.zip › Supp-II.pdf]

Supplementary Table II. The performance of the GBA-based method yielded by the five-fold cross-validation

Index of part	*k*	1	2	3	4	5	6	7	8	9
1	Recall	0.207	0.276	0.310	0.345	0.414	0.414	0.448	0.448	0.448
	Precision	0.061	0.046	0.036	0.032	0.032	0.027	0.025	0.023	0.021
	F1-measure	0.094	0.078	0.064	0.058	0.059	0.051	0.048	0.044	0.040
2	Recall	0.207	0.241	0.276	0.276	0.310	0.345	0.345	0.414	0.414
	Precision	0.059	0.038	0.032	0.024	0.023	0.022	0.019	0.020	0.018
	F1-measure	0.092	0.066	0.057	0.045	0.042	0.041	0.036	0.038	0.035
3	Recall	0.103	0.241	0.345	0.414	0.448	0.483	0.552	0.552	0.586
	Precision	0.031	0.040	0.039	0.036	0.032	0.030	0.030	0.027	0.026
	F1-measure	0.047	0.069	0.069	0.066	0.060	0.057	0.057	0.051	0.049
4	Recall	0.172	0.276	0.310	0.345	0.379	0.414	0.448	0.448	0.448
	Precision	0.052	0.045	0.035	0.031	0.028	0.026	0.025	0.022	0.020
	F1-measure	0.079	0.078	0.063	0.056	0.052	0.049	0.047	0.042	0.039
5	Recall	0.200	0.367	0.500	0.533	0.533	0.533	0.533	0.533	0.567
	Precision	0.064	0.062	0.061	0.052	0.043	0.037	0.032	0.029	0.028
	F1-measure	0.097	0.106	0.109	0.094	0.079	0.068	0.061	0.055	0.052
Index of part	*k*	20	30	40	50	60	70	80	90	100
1	Recall	0.517	0.621	0.724	0.724	0.724	0.793	0.793	0.793	0.828
	Precision	0.012	0.011	0.010	0.009	0.008	0.008	0.007	0.006	0.006
	F1-measure	0.024	0.022	0.021	0.018	0.015	0.015	0.014	0.013	0.013

2	Recall	0.621	0.621	0.655	0.724	0.793	0.897	0.897	0.897	0.897
	Precision	0.014	0.011	0.009	0.009	0.008	0.008	0.008	0.007	0.007
	F1-measure	0.028	0.021	0.018	0.017	0.017	0.017	0.015	0.014	0.013
3	Recall	0.759	0.793	0.828	0.828	0.828	0.828	0.862	0.862	0.862
	Precision	0.017	0.014	0.011	0.010	0.009	0.008	0.007	0.007	0.006
	F1-measure	0.034	0.027	0.023	0.019	0.017	0.015	0.014	0.013	0.013
4	Recall	0.655	0.690	0.828	0.828	0.862	0.862	0.862	0.862	0.862
	Precision	0.015	0.012	0.012	0.010	0.009	0.008	0.007	0.007	0.006
	F1-measure	0.030	0.024	0.023	0.020	0.018	0.016	0.015	0.014	0.013
5	Recall	0.633	0.633	0.633	0.700	0.700	0.733	0.767	0.800	0.800
	Precision	0.016	0.012	0.010	0.009	0.008	0.007	0.007	0.007	0.006
	F1-measure	0.031	0.023	0.019	0.018	0.016	0.015	0.014	0.013	0.013
