# Supplementary material for: Determination of Genes Related to Uveitis by Utilization of the Random Walk with Restart Algorithm on a Protein–Protein Interaction Network
Source: Int J Mol Sci. 2017 May 13;18(5):1045. doi: 10.3390/ijms18051045 (PMC5454957; doi:10.3390/ijms18051045)
Supplement: Supplementary file 1 [file ijms-18-01045-s001.zip › Supp-III.pdf]

Supplementary Table III. Uveitis-related genes and their Ensembl IDs

Gene symbol	Ensembl ID
C2	ENSP00000372853<br>ENSP00000405800<br>ENSP00000402278<br>ENSP00000364660<br>ENSP00000392835<br>ENSP00000407961<br>ENSP00000299367
CD4	ENSP00000011653
CFB	ENSP00000416561<br>ENSP00000410815
CFH	ENSP00000356399
CRP	ENSP00000255030
DMD	ENSP00000354923
F5	ENSP00000356771
IL2	ENSP00000226730
IL6	ENSP00000258743
IL7	ENSP00000263851
IL8	ENSP00000306512
MIF	ENSP00000215754
MIP	ENSP00000257979
TNF	ENSP00000398698<br>ENSP00000389265<br>ENSP00000389492<br>ENSP00000392858<br>ENSP00000389490<br>ENSP00000410668<br>ENSP00000372988<br>ENSP00000365290
ACTG2	ENSP00000295137
CARD15	-
CARD9	ENSP00000360797
CASP1	ENSP00000410076
CASP3	ENSP00000311032
CCL2	ENSP00000225831
CCL5	ENSP00000293272
CCR1	ENSP00000296140
CCR2	ENSP00000292301
CCR3	ENSP00000350003
CCR4	ENSP00000332659

CCR5	ENSP00000292303
CCR6	ENSP00000339393
CCR7	ENSP00000246657
CD25	-
CD27	ENSP00000266557
CD274	ENSP00000370989
CD28	ENSP00000324890
CD30	-
CD40	ENSP00000361359
CD45	-
CD57	-
CD69	ENSP00000228434
CD74	ENSP00000009530
CD86	ENSP00000332049
CNKSR3	ENSP00000356182
CTLA4	ENSP00000303939
CTSC	ENSP00000227266
CTSH	ENSP00000220166
CX3CL1	ENSP00000006053
CX3CR1	ENSP00000382166
CXCL10	ENSP00000305651
CXCR3	ENSP00000362795
ECAD	-
EOMES	ENSP00000295743
ERAP1	ENSP00000296754
ERAP2	-
FCRL3	ENSP00000357167
FOXP3	ENSP00000365380
GIMAP2	ENSP00000223293
GIMAP4	ENSP00000255945
GLI1	ENSP00000228682
GPR35	ENSP00000322731
GPR37	ENSP00000306449
GPR65	ENSP00000267549
GZMH	ENSP00000216338
HLA-A	ENSP00000416233<br>ENSP00000388526<br>ENSP00000398188<br>ENSP00000373114<br>ENSP00000388724<br>ENSP00000410645<br>ENSP00000366005
HLA-B	ENSP00000399168

	ENSP00000400842
HLA-C	ENSP00000365402<br>ENSP00000372975<br>ENSP00000390282<br>ENSP00000407431<br>ENSP00000397867<br>ENSP00000383245<br>ENSP00000413992
ICAM1	ENSP00000264832
IDO1	ENSP00000253513
IFNG	ENSP00000229135
IL10	ENSP00000412237
IL12A	ENSP00000303231
IL12B	ENSP00000231228
IL12RB2	ENSP00000262345
IL17A	ENSP00000344192
IL18	ENSP00000280357
IL1R1	ENSP00000233946
IL1R2	ENSP00000330959
IL21	ENSP00000264497
IL22	ENSP00000329384
IL23	-
IL23R	ENSP00000321345
IL4R	ENSP00000170630
IL7R	ENSP00000306157
IRF5	ENSP00000349770
JAG1	ENSP00000254958
JAK2	ENSP00000371067
KLRC4	ENSP00000310216
LCE3B	ENSP00000335358
LCE3C	ENSP00000334644
LFNG	ENSP00000222725
LNPEP	ENSP00000231368
MAPK9	ENSP00000321410
MEFV	ENSP00000219596
NKAP	ENSP00000360464
NOD2	ENSP00000300589
NPEPPS	ENSP00000320324
NQO1	ENSP00000319788
PDCD1	ENSP00000335062
PSMG1	ENSP00000329915
PTPN22	ENSP00000352833
RAB27A	-

RALGPS2	ENSP00000356607
RUNX3	ENSP00000343477
SPP1	ENSP00000378517
STAT3	ENSP00000264657
STAT4	ENSP00000351255
SUMO4	ENSP00000318635
TAB2	ENSP00000286332
TBX21	ENSP00000177694
TGFB2	ENSP00000355896
TGFBR3	ENSP00000212355
TGM2	ENSP00000355330
TLR4	ENSP00000363089
TNFAIP3	ENSP00000237289
TYK2	ENSP00000264818
UBAC2	ENSP00000383911
UBE2L3	ENSP00000344259
VEGFB	ENSP00000311127
WNT5B	ENSP00000308887
ZMIZ1	ENSP00000334474
HLA-DQA1	ENSP00000401760<br>ENSP00000414360<br>ENSP00000372738<br>ENSP00000339398<br>ENSP00000409127<br>ENSP00000387892
HLA-DQB1	ENSP00000382025<br>ENSP00000382018<br>ENSP00000372734<br>ENSP00000382034
HLA-DRB1	ENSP00000353099
PDCD1LG2	-
